# Elucidation of Genetic Interactions in the Yeast GATA-Factor Network Using Bayesian Model Selection

**DOI:** 10.1371/journal.pcbi.1004784

**Published:** 2016-03-11

**Authors:** Andreas Milias-Argeitis, Ana Paula Oliveira, Luca Gerosa, Laura Falter, Uwe Sauer, John Lygeros

**Affiliations:** 1 Department of Biosystems Science and Engineering, ETH Zurich, Basel, Switzerland; 2 Institute of Molecular Systems Biology, ETH Zurich, Zurich, Switzerland; 3 Automatic Control Laboratory, ETH Zurich, Zurich, Switzerland; Ottawa University, CANADA

## Abstract

Understanding the structure and function of complex gene regulatory networks using classical genetic assays is an error-prone procedure that frequently generates ambiguous outcomes. Even some of the best-characterized gene networks contain interactions whose validity is not conclusively proven. Founded on dynamic experimental data, mechanistic mathematical models are able to offer detailed insights that would otherwise require prohibitively large numbers of genetic experiments. Here we attempt mechanistic modeling of the transcriptional network formed by the four GATA-factor proteins, a well-studied system of central importance for nitrogen-source regulation of transcription in the yeast *Saccharomyces cerevisiae*. To resolve ambiguities in the network organization, we encoded a set of five interactions hypothesized in the literature into a set of 32 mathematical models, and employed Bayesian model selection to identify the most plausible set of interactions based on dynamic gene expression data. The top-ranking model was validated on newly generated GFP reporter dynamic data and was subsequently used to gain a better understanding of how yeast cells organize their transcriptional response to dynamic changes of nitrogen sources. Our work constitutes a necessary and important step towards obtaining a holistic view of the yeast nitrogen regulation mechanisms; on the computational side, it provides a demonstration of how powerful Monte Carlo techniques can be creatively combined and used to address the great challenges of large-scale dynamical system inference.

## Author Summary

Gene regulatory networks underlie all key processes that enable a cell to maintain long-term homeostasis in a changing environment. Understanding the structure and function of complex gene networks is an experimentally difficult and error-prone procedure. Mechanistic mathematical modeling promises to alleviate these problems, as we demonstrate here for the yeast GATA-factor network, the central controller of the cellular response to nitrogen source quality. Despite years of targeted studies, the interaction pattern of this network is still not known precisely. To resolve several still-remaining ambiguities, we generated a set of alternative mathematical models, and compared them against each other using Bayesian model selection based on dynamic gene expression data. The top-ranking model was then validated on a separate, newly generated dataset. Our work thus provides new insights to the mechanism of nitrogen regulation in yeast, while at the same time overcoming some key computational inference problems for large models in systems biology.

## Introduction

Decades of research on gene regulatory networks have provided us with a wealth of knowledge on their topologies. However, even the best characterized networks contain many ambiguous interactions, discovered using a variety of experimental techniques that often cannot validate their presence conclusively. Moreover, knowledge of a “static” gene regulatory interaction pattern consisting of multiple feedback and/or feedforward loops cannot provide insight into which regulatory interactions are functionally relevant at a given time and cellular context. Dynamic mechanistic modeling informed by quantitative, time-resolved experimental data can provide discriminatory resolution and is thus an indispensable tool for understanding the structure and function of complex gene networks.

The GATA gene regulatory network in the yeast *Saccharomyces cerevisiae* is an example of a well-characterized transcriptional network that contains multiple feedback loops. This feedback has confounded the inference of regulatory interactions from experiments and led to several speculative, unverified regulatory hypotheses. The network is composed of four transcription factors (TFs) that respond to the quality of the available nitrogen source and regulate the transcriptional response of around 90 genes related to nitrogen catabolism. Specifically, the network comprises the transcriptional activators Gat1 and Gln3 and the transcriptional repressors Dal80 and Gzf3, all four of which recognize the same core motif in the promoter regions of their gene targets, including the promoters of *GAT1*, *DAL80* and *GZF3*. This cross-regulation provides tight control over the transcription of genes encoding for permeases and catabolic enzymes required for the utilization of poor nitrogen sources when more preferred sources are available. This phenomenon is generally referred to as Nitrogen Catabolite Repression (NCR) [1]. Depletion of rich nitrogen sources (e.g. glutamine) results in the relief of NCR, providing cells with the metabolic repertoire to scavenge for and utilize non-preferred nitrogen sources (e.g. proline). Yeast cells monitor the nitrogen availability by a yet unknown mechanism involving the rapamycin-sensitive TORC1 pathway, among possibly other signaling pathways, and accordingly control the NCR activity by modulating the subcellular localization of the two GATA activators [1–4]. In particular, TORC1 is known to mediate the localization of Gln3 and Gat1 through phosphorylation: during growth on poor nitrogen sources, Gln3 and Gat1 are not phosphorylated and localize in the nucleus to activate transcription, while in the presence of a good nitrogen source they are phosphorylated and remain predominantly cytoplasmic [5–8], although their phosphorylation pattern does not always correlate with their localization [9]. TORC1 inhibition with the antifungal agent rapamycin results in a nitrogen starvation phenotype that induces NCR-sensitive gene expression even in the presence of a good nitrogen source [10–12], a property frequently explored to mimic a downshift from a good to a poor nitrogen source, with concomitant relief of NCR [13].

**Fig 1:**
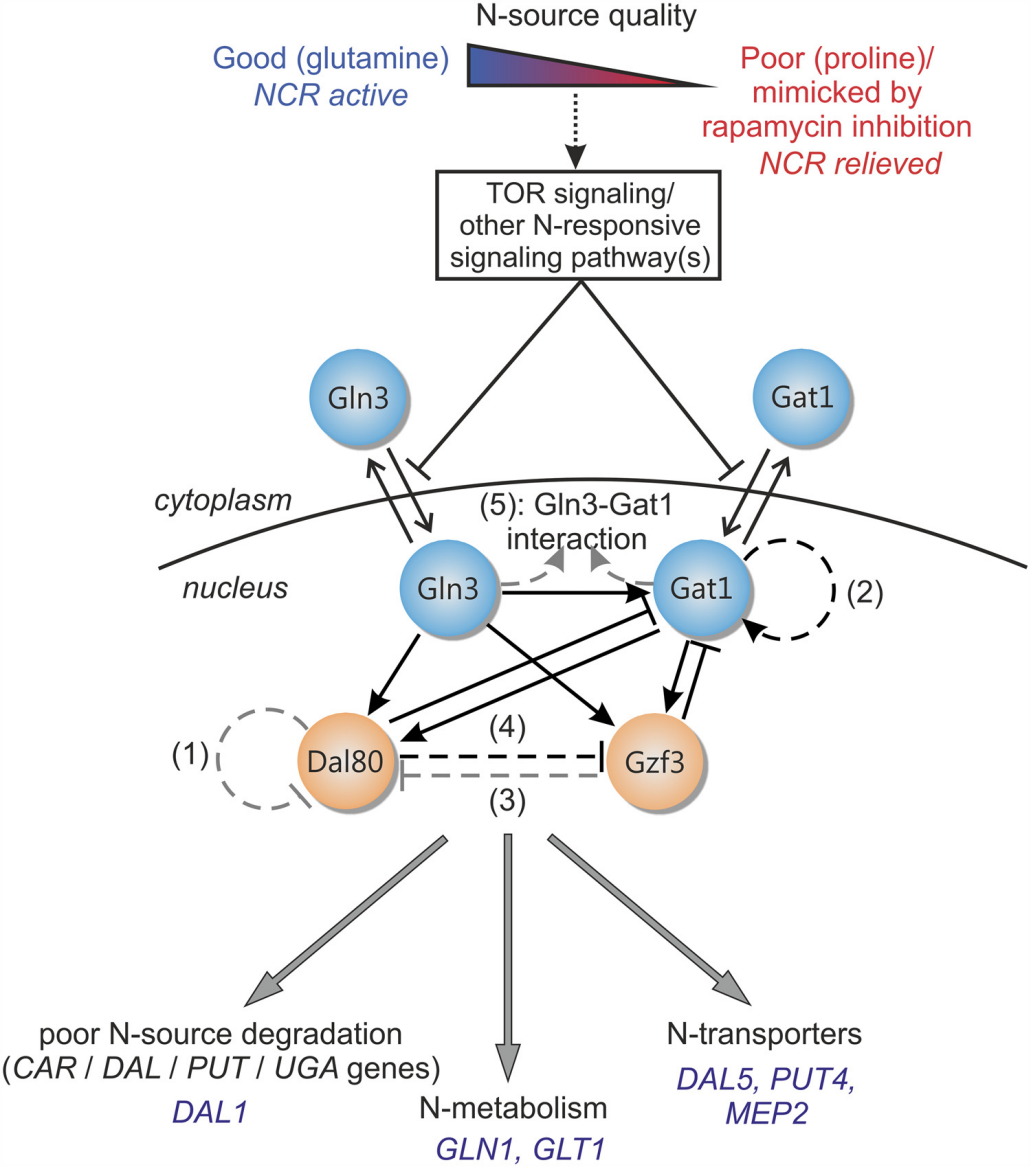
A schematic view of NCR regulation in yeast by the GATA-network. The yeast GATA gene regulatory network responds to the quality of the available nitrogen source and accordingly regulates the transcription of genes related to nitrogen catabolism, a phenomenon termed nitrogen catabolite repression (NCR). Genes subject to NCR are repressed during growth in a good nitrogen source, and derepressed during growth in a poor one, which can be mimicked by rapamycin addition. The signal is mediated via TORC1 and other nitrogen-responsive signaling pathways, thereby determining the nuclear-cytosolic localization of the two GATA activators, Gln3 and Gat1 (blue circles). The two GATA repressors, Dal80 and Gzf3 (orange circles) are only nuclear and active as dimers. The four GATA-factors cross-regulate each other’s gene expression extensively. Pointed arrows denote positive transcriptional regulation (gene activation), white T-arrows denote negative transcriptional regulation (gene repression). The established regulatory interactions are represented as solid black arrows. The five hypothesized regulatory interactions compiled from the literature are represented as dashed arrows. Numbers next to dashed arrows denote hypothesis numbers used in model labeling (see Section 1.2 of S1 Text for details). The arrows corresponding to hypothesized interactions contained in the top-ranking model are colored black. GATA-factor controlled genes mainly fall under three functional groups: genes involved in poor nitrogen source degradation, nitrogen metabolism and transport of nitrogen sources. The specific target genes considered in this study are listed in blue under these three categories.

Despite many years of targeted studies, parts of the GATA network topology remain obscure, since the complex interaction pattern complicates the interpretation of available experimental data. Various transcriptional interactions have been suggested over the years, but have remained unverified by subsequent observations. For example, results in [14–16] suggest Dal80 self-repression, yet its binding to the *DAL80* promoter remains unverified. Moreover, the available experimental data (Northern blots [14] and LacZ assays [15]) cannot preclude the possibility that the observed increase in Dal80 expression in a Δ*dal*80 background is due to indirect regulatory interactions. Similarly, the negative regulation of *DAL80* by Gzf3 has been inferred from assays (LacZ [15] and Northern blots [16] in a Δ*dal*80 background) that cannot differentiate between direct and indirect effects. Overall, a careful examination of the experimental evidence reported in the literature revealed in total five interactions whose validity cannot be unambiguously concluded. These hypothesized interactions are indicated with dashed lines on Fig 1. A detailed literature-based justification for the consideration of these interactions as hypotheses is presented in Section 1.2 of S1 Text.

To resolve such ambiguities we used Bayesian model selection combined with dynamic gene expression data. Based on an extensive literature search, we first compiled a set of five interactions that have been hypothesised in the literature, but remain unvalidated. We next encoded these biological hypotheses into alternative mathematical model structures and formulated a Bayesian model selection problem [17–21]. Exploiting special structures present in the resulting dynamical models and by creatively using Monte Carlo-based inference, a workflow to carry out inference for dynamical systems with very high-dimensional parameter spaces was developed. This allowed the systematic comparison of alternative models against each other and the selection of the best candidates based on the measured dynamic mRNA responses of target genes under a nitrogen upshift perturbation and rapamycin treatment. The top-ranking model was subsequently validated using experimental data generated in GATA factor deletion strains carrying a GFP reporter. Our results provide strong insights into the long standing open issues surrounding the transcriptional regulation of NCR. They provide strong evidence for Gat1 positive autoregulation, for Dal80 repression of *GZF3* and that the two activators do not interact on the GATA-factor promoters. On the other hand, repression of *DAL80* by Gzf3 appears not to be essential, and there is no strong support in favor of Dal80 self-repression. The top-ranking model structure was subsequently used to provide quantitative insights into network function that would be hard to obtain experimentally. With our system being among the largest and most complex considered for Bayesian model selection to date, we were also able to demonstrate how powerful Monte Carlo estimation methods can be efficiently used to address large-scale inference problems in computational biology.

## Results

### Core model formulation

To gain a better understanding of the transcriptional control of NCR by the yeast GATA gene regulatory network, we compiled a literature-based list of its components and their interactions. The established knowledge of how the GATA-factors regulate the expression of each other is depicted with solid lines on Fig 1 (a list of relevant references is provided in Section 1.1 of S1 Text), while hypothesized interactions are indicated with dashed lines and presented in detail in Section 1.2 of S1 Text.

To encode mathematically the established biological knowledge on the GATA network, as well as the hypothesized interactions, we generated a set of ordinary differential equation models that capture the evolution of all chemical species involved (mRNAs, proteins and protein complexes). The models account for mechanistic details that describe the rates of mRNA transcription, protein production, protein degradation, nuclear-cytosolic translocation and dimerization, formalized in a total of 13 dynamical states and embedding three input variables. Moreover, they take as input an external signal that reflects the quality of the nitrogen source and determines the translocation rates for the two activators. An additional, secondary input of the system is the Gln3 mRNA concentration. The state variables contained in the model describe the mRNA concentrations, the nuclear / cytosolic concentration of the activators, and the monomeric / dimeric concentration of the repressors. Further details can be found in Materials and Methods, and Section 2 of S1 Text.

**Fig 2:**
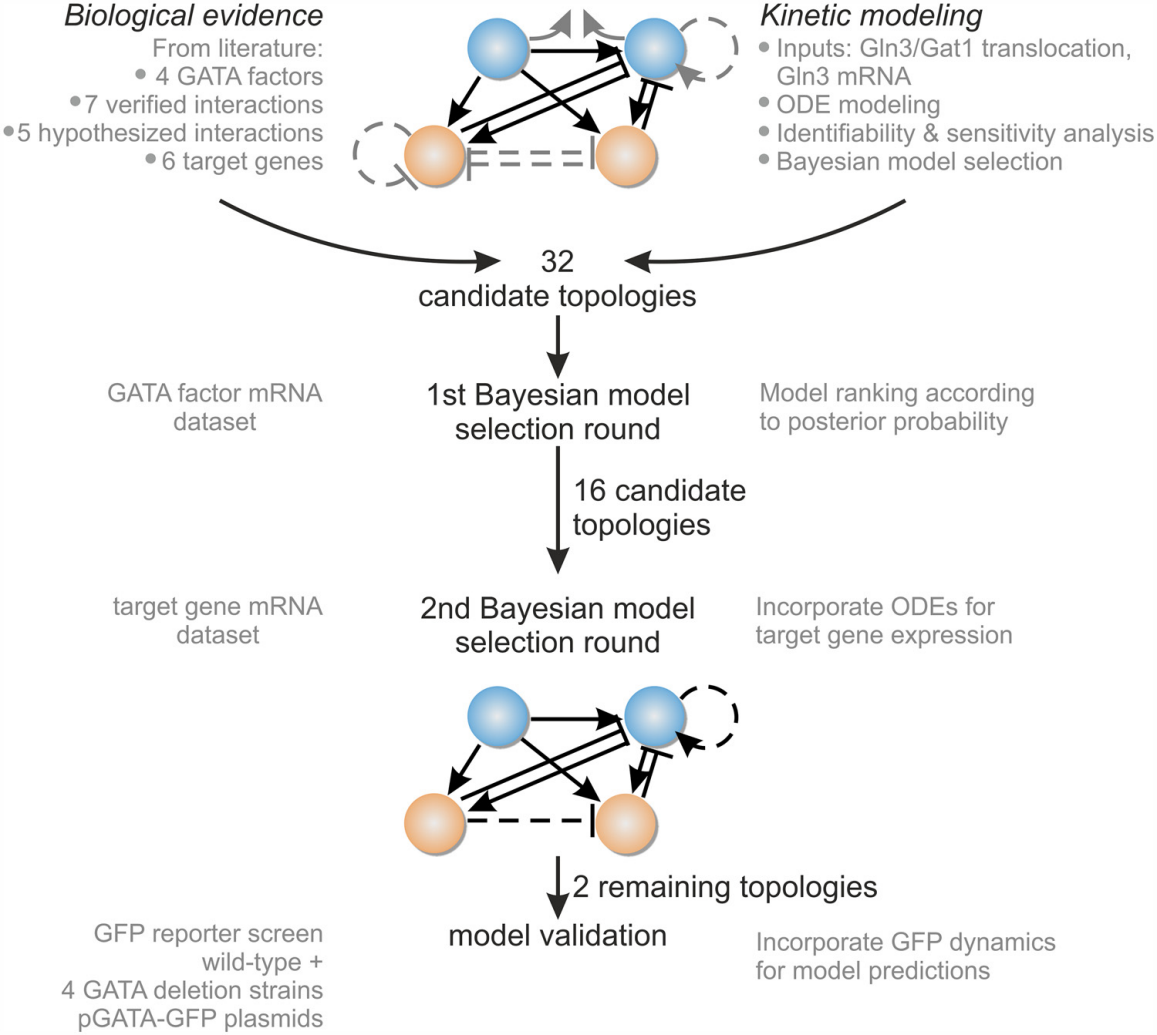
Model selection and validation workflow.

The basic model structure based solely on the well-established GATA network interactions comprises 41 parameters. To determine if any, or a combination, of the hypothesized interactions are more plausible given the experimental observations, we next encoded the five biological hypotheses into alternative mathematical model structures. Since the five hypothesized interactions are not mutually exclusive, a total of 2^5^ − 1 = 31 additional alternative model structures, $M_{k}$, were generated, each encoding a particular combination of interactions. Each model structure accounted for 41 to 50 parameters, depending on the combination of hypotheses. The structures were named as follows: starting from the *full* model ($M_{0}$) that contains *all* hypothesized interactions, we denoted each subsequent model by the interactions it is *missing*. For example, model $M_{124}$ misses the interactions suggested by hypotheses 1, 2 and 4, according to the enumeration of interactions presented on Fig 1. In order to verify the plausibility of the hypothesized interactions based on the improved predictions of an augmented model structure relative to others, we proceeded with two rounds of model selection and an experimental model validation step as summarized in Fig 2.

### First model selection round

Model selection was based on an existing dataset of mRNA abundances previously quantified for wild type yeast subject to an upshift from proline to glutamine (Pro→Gln) and to a downshift induced by rapamycin addition to glutamine-grown cells (Gln+Rap) [22].

**Fig 3:**
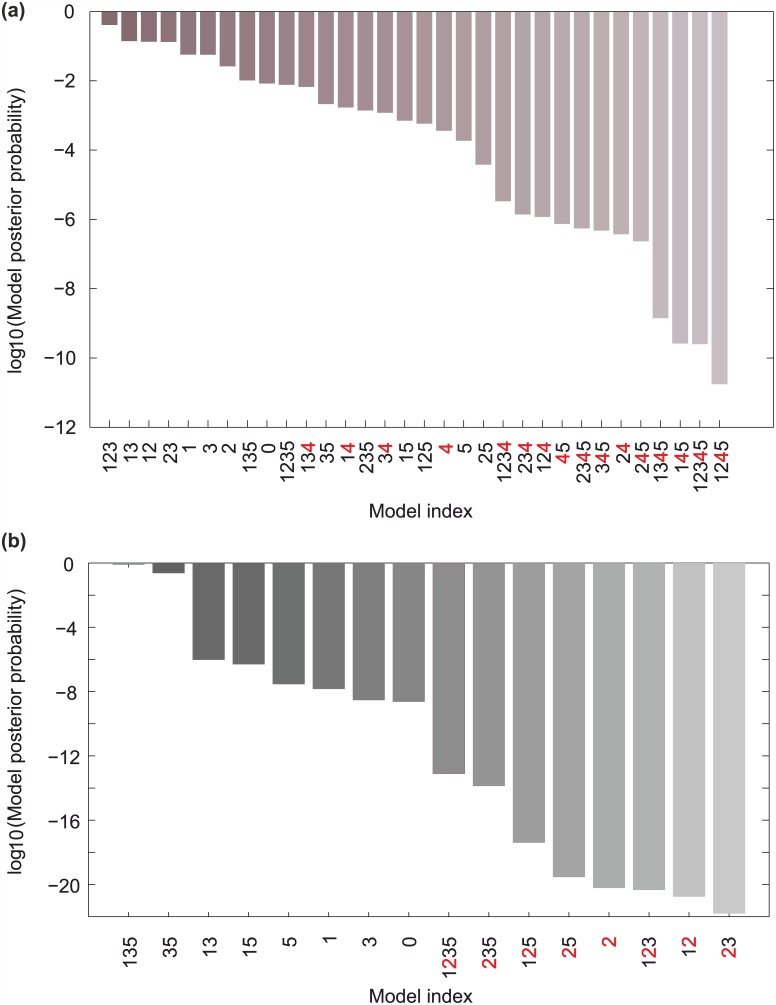
Summary of the results from the two model selection rounds. (a) Logarithm of the model posterior probabilities obtained from averaging the outcomes of three runs of the SMC algorithm using the GATA TF mRNA data, under a uniform model prior. The results of individual runs are reported in Section 5.3 of S1 Text. Models missing the interaction of hypothesis 4 are clearly ranked lower than the rest (b) Logarithm of model posterior probabilities for the 16 models considered in the second model selection round. The numbers reported were obtained for each model by averaging over all possible products of estimates of $P(M_{k}|D_{TF})$ and two estimates of $F(D_{targets},M_{k})$. Models 35 and 135 clearly stand out, as their posterior probabilities are distinctively greater than the rest, with 90% confidence intervals (computed from the data presented on Table G in S1 Text) equal to 0.23±0.11 and 0.77±0.35 respectively. Note that all models missing interaction 2 are ranked lower.

To assess which network topology among the alternatives is supported by the GATA-factor gene expression data, denoted *D*_*TF*_, we performed a first round of Bayesian model selection. According to the Bayesian approach, detailed in Section 3.1 of S1 Text, all 32 alternative model structures were initially assigned an equal level of plausibility (prior probability), $P(M_{k})$. Subsequently, the prior model probabilities were updated using the experimental data to estimate $P(D_{TF}|M_{k})$ (called the *evidence* for model $M_{k}$) to obtain the posterior model probabilities, $P(M_{k}|D_{TF})$, using Bayes’ formula: $P\left(M_{k}|D_{TF}\right)\propto P\left(D_{TF}|M_{k}\right)P\left(M_{k}\right)$. These quantities, shown on Fig 3(a), encode the plausibility of each model structure after incorporating the experimental observations.

To enable the calculation of posterior probabilities on the high-dimensional model parameter spaces, we used a Sequential Monte Carlo (SMC) sampler [23] (S1 Text, Section 3.2), which was developed based on a comparison of different advanced sampling methods [24]. Sequential Monte Carlo is a family of powerful algorithms that tackle the problem of sampling from an intractable (i.e. hard-to-sample) distribution by starting from a tractable one and moving through a sequence of artificial intermediate distributions. The algorithms include several user-defined settings that can greatly affect their performance, and successful application of these methods had never been reported for dynamical systems of size comparable to the one treated here. Our SMC sampler was able to explore efficiently the parameter spaces thanks to an adaptive sampling mechanism based on density estimation via Gaussian mixtures, which is able to overcome the common problems faced by traditional sampling approaches in high-dimensional settings. The algorithm was thus able to provide low-variance estimates (Section 5.2 and Fig. I and Fig. N in S1 Text) that enabled us to reliably rank the alternative model structures according to their posterior probabilities (Fig 3(a)). Following the interpretation of model evidence ratios given in [25] and given that all model priors are equal, a ratio of posterior probabilities greater than 100 can be interpreted as decisive support of the data in favor of one model against another. Based on these posterior probabilities no model stands out clearly from the rest: the ratio of posterior probabilities between the top-ranking and the rest of the models is not great enough to provide decisive support in its favor (Fig 3(a)).

Although the available gene expression data alone could not provide unambiguous evidence in favor of a single model structure, we observed a set of candidate models whose posterior probabilities are clearly higher from the rest. Interestingly, all these structures contain the repression of *GZF3* by Dal80 (hypothesis 4). We therefore eliminated all 16 model structures missing this interaction, and proceeded to discriminate among the remaining 16 models that account for the repression of *GZF3* by Dal80.

### Extended model formulation

An indirect way to observe the changes in the GATA-factor transcription activities, is to consider their regulatory effect on known target genes. With the aim of obtaining additional model resolution to sharpen the model selection results, we extended the core model to account for additional target genes regulated by the GATA factors and for which gene expression data is also available. Yeast GATA factors are the main regulators of around 90 genes involved in nitrogen catabolic gene expression and core nitrogen metabolism [26, 27]. Of these, we selected six targets that are known to be mainly controlled by the GATA factors during NCR—*DAL1* (allantoinase), *DAL5* (allantoin permease), *GLN1* (glutamine synthetase), *GLT1* (glutamate synthetase), *MEP2* (ammonium permease) and *PUT4* (proline permease) (Fig 1) -, and used them in the subsequent model selection process. The exact regulatory influence of each GATA factor on each target is still elusive and seems to differ depending on the structure of their promoter, such as the number and spacing of binding sites. More information about these genes and a justification for their choice is given in Section 1.3 of S1 Text.

To account for the gene expression data from these GATA targets (denoted *D*_*targets*_ and previously obtained in [22]), we expanded the initial GATA-factor model by six additional states, representing the target mRNAs. Since the precise regulation pattern (number of GATA regulators and interaction strengths) of each target is uncertain, each target equation contributes seven unknown parameters to the extended model (cf. S1 Text, Section 2.4). This leads to a significant increase in computational cost of the model selection process, as the total number of parameters rises to 92 in the case of the extended model $M_{0}^{*}$. To the best of our knowledge, no currently available Monte Carlo algorithm is able to reliably sample parameter spaces for dynamical systems of this size, a computational challenge even when compared to existing studies with thousands of variables for static Bayesian hierarchical models [28]. We have been able to circumvent this limitation by employing a novel modular sampling approach, in which we exploit the unidirectional flow of state information in the extended system. This property allowed us to decompose the total model evidence calculation into a product of several factors, each of which can be obtained with much smaller computational effort. The theoretical justification and the practical implementation of our approach are provided in Materials and Methods, and Section 4 of S1 Text respectively.

### Second model selection round

To further discriminate among the remaining 16 hypotheses, we applied a second round of Bayesian model selection to the extended model formulation. Following the modular sampling approach described in Section 4 of S1 Text, the posterior probability of the *k*-th augmented model, $M_{k}^{*}$ can be obtained from the formula

$$\begin{array}{c} P\left(M_{k}^{*}|D_{TF},D_{targets}\right)\propto P\left(M_{k}|D_{TF}\right)F\left(D_{TF},D_{targets},M_{k}\right), \end{array}$$

where *D*_*TF*_ and *D*_*targets*_ denote the TF and target gene expression datasets respectively, $M_{k}$ is the *k*-th TF model structure corresponding to a combinatorial topology of four possible interactions (hypotheses 1, 2, 3 and 5), and *F* is a multiplicative factor that can be estimated by Monte Carlo integration, as described in Section 4 of of S1 Text. Note that the posterior probability of the original model, $P(M_{k}|D_{TF})$, was already available from the first model selection round. Table I in S1 Text summarizes the estimates of the multiplicative factors *F* for the 16 model structures considered in this second round.

Putting together the estimates for $P(M_{k}|D_{TF})$ with the estimates of *F*, we obtained the model posteriors shown on Fig 3(b). We clearly observe that all structures lacking hypothesis 2 are strongly penalized, as their posterior probabilities are the lowest among all structures considered. This result suggests that Gat1 self-activation drastically changes a model’s capacity to accommodate the target gene expression data, while the TF dataset is less discriminatory by itself. Overall standing out as the most plausible models were $M_{135}$ and $M_{35}$, both missing interactions corresponding to hypotheses 3 and 5. The presence or absence of hypothesis 1 does not make a significant difference between the two models, since the posterior probabilities differ only by a small factor (3.3). This may arise from the fact that the Bayesian methodology implicitly penalizes Model $M_{35}$ relative to $M_{135}$ because of its extra free parameter. Thus, after two rounds of Bayesian model selection, the initial list of 32 candidate models was reduced to two top-ranking topologies. These two top models strongly support the role of Gat1 self-activation and of *GZF3* repression by Dal80 (hypotheses 2 and 4), and discard the relevance of *DAL80* repression by Gzf3 and Gln3-Gat1 interaction (hypotheses 3 and 5) in regulating the yeast NCR response. In the subsequent sections, the top-ranked model ($M_{135}$) will be used, due to its reduced complexity relative to $M_{35}$.

### Model validation

To validate the results of the final model selection round, we challenged model $M_{135}$ to predict the outcome of additional experiments. To this end, we designed an experiment to dynamically monitor GFP expression from GATA promoters in the absence of each of the four GATA factors during the same two shifts used for model selection (Pro→Gln and Gln+Rap). Specifically, we constructed a collection of GFP-reporter plasmids expressing the yeast Enhanced Green Fluorescent Protein (yEGFP) gene immediately downstream of the native promoter of each GATA factor (S2 Text). Each of the four plasmids and the control vector were transformed into the wild type and all four GATA single deletion mutants, yielding a total of 25 yeast strains (S2 Text). The strains were cultivated in liquid culture in microtiter plates and monitored online for biomass and GFP evolution (S1 Dataset). Glutamine and rapamycin were added to cells growing exponentially in proline or glutamine, respectively. The fluorescence and biomass measurements were background-corrected and processed following the approach described in [29] to obtain the relative concentration of GFP, as well as the time-dependent growth rate.

In parallel, we simulated the GFP response of each GATA promoter under the experimentally defined conditions, using the topology of the top-ranked model $M_{135}$ and an adjusted set of differential equations that account for the extra species involved (GFP mRNA, immature and mature GFP). Further details can be found in Section 2.5 of S1 Text.

**Fig 4:**
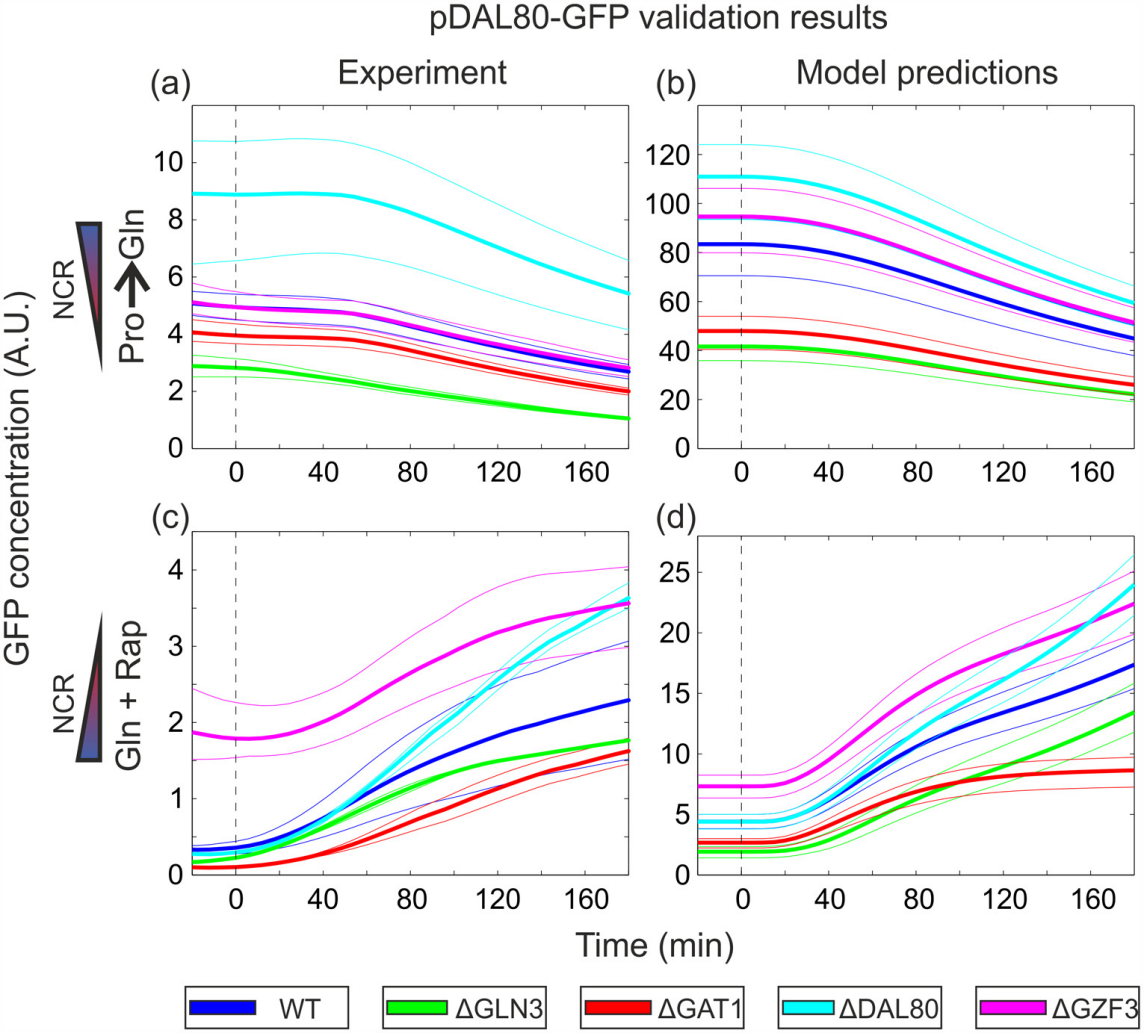
Data and model predictions for the yEGFP reporter driven by the *DAL80* promoter (pDAL80-GFP). (a) Relative GFP concentrations for the Pro→Gln shift. Upper and lower band limits: maximum and minimum experimentally observed values. Solid lines: mean of all biological replicates. (b) Model predictions of relative GFP concentrations for the Pro→Gln shift, obtained from 100 randomly sampled posterior samples for the regulation function parameters. Upper and lower band limits: 80% and 20% quantiles. (c) Relative GFP concentrations for the Gln+Rap shift. Upper and lower band limits, solid lines: same as in (a). (d) Model predictions of relative GFP concentrations for the Gln+Rap shift. Prediction bands obtained as in (b). Note: the Y-axis scale units are arbitrary and different between the figures on the left and right columns.

**Fig 5:**
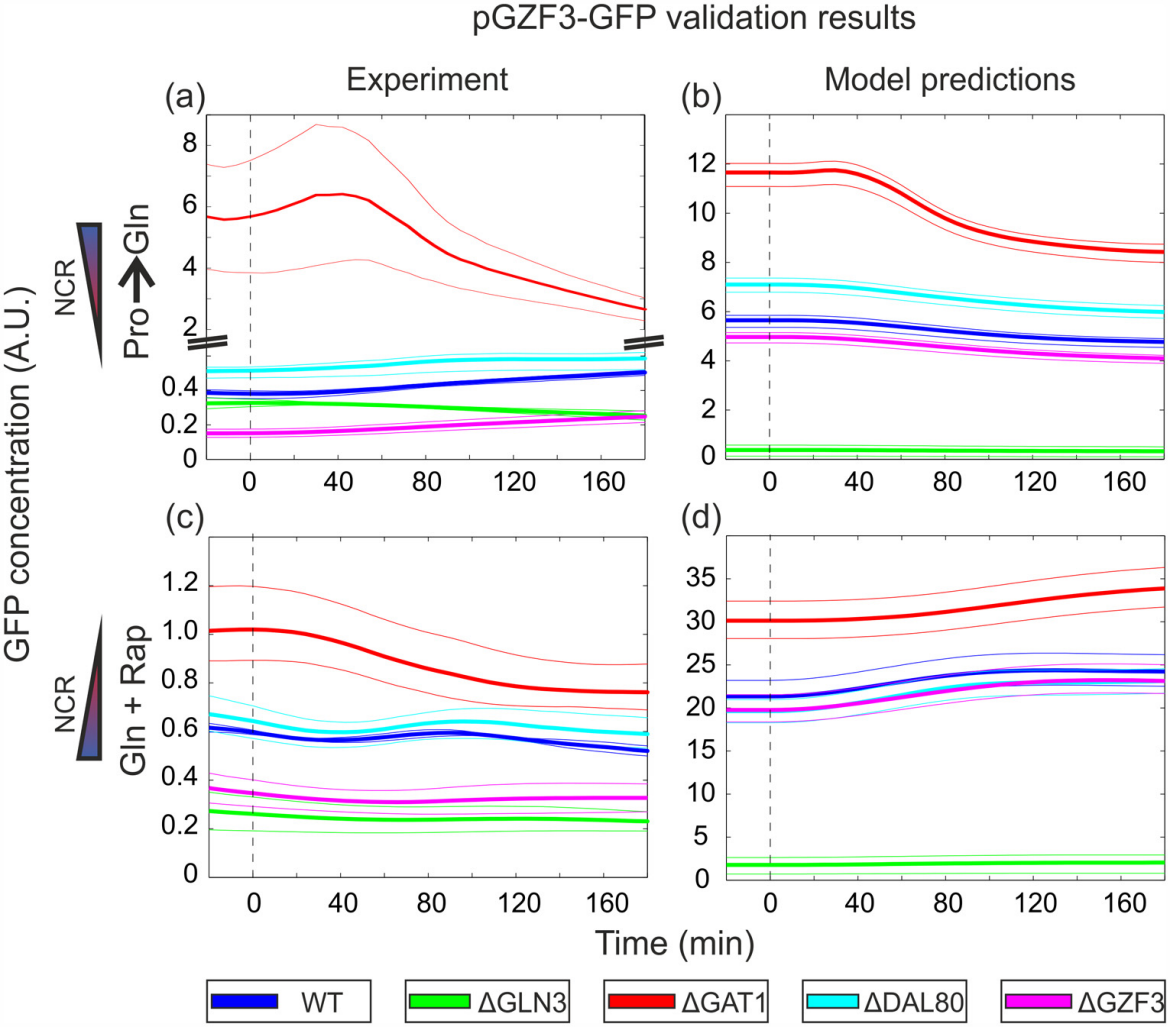
Data and model predictions for the yEGFP reporter driven by the *GZF3* promoter (pGZF3-GFP). (a) Relative GFP concentrations for the Pro→Gln shift. Upper and lower band limits: maximum and minimum experimentally observed values. Solid lines: mean of all biological replicates. Due to the extremely low GFP signal above background fluorescence, the small trends displayed by the curves may actually be artifacts of background normalization. Although the ordering of the responses of the various deletion strains is consistent across all replicates, the only reliably inferred response is that of the Δ*gat*1 strain, in which GFP appears significantly upregulated compared to wild-type. (b) Model predictions of relative GFP concentrations for the Pro→Gln shift, obtained from 100 randomly sampled posterior samples for the regulation function parameters. Upper and lower band limits: 80% and 20% quantiles. (c) Relative GFP concentrations for the Gln+Rap shift. Upper and lower band limits, solid lines: same as in (a). Similarly to (a), the small trends in the responses may be artifacts. However, the ordering of the responses of the various deletion strains is consistent across all replicates. (d) Model predictions of relative GFP concentrations for the Gln+Rap shift. Prediction bands obtained as in (b). Note: the Y-axis scale units are arbitrary and different between the figures on the left and right columns.

The outcome of the GATA-factor model $M_{135}$, augmented with the GFP reporter dynamics, was used for a qualitative comparison between the predicted GFP evolution and the experimental data. Experimental and predicted results for strains harboring the *DAL80* and the *GZF3* reporter GFP are shown in Figs 4 and 5, respectively (very similar predictions were obtained with model $M_{35}$). Strains harboring the *GLN3* reporter showed no significant changes in GFP production rate (S1 Dataset), in line with the previously described observations that *GLN3* is regulated in a NCR-independent manner. The plasmid harboring the *GAT1* reporter did not show any GFP signal for unclear technical reasons that could not be addressed, while the *GZF3* promoter signal was very close to background in most deletion strains. Overall, despite some caveats that preclude their quantitative comparison (S1 Text, Subsection 2.5.1), predictions with model $M_{135}$ match experimental outcomes well in terms of the ordering and general trend of the responses, reinforcing our model-based conclusions on the presence of the hypothesized interactions 2 and 4 depicted on Fig 1.

### Model-based insights on the GATA network function

**Fig 6:**
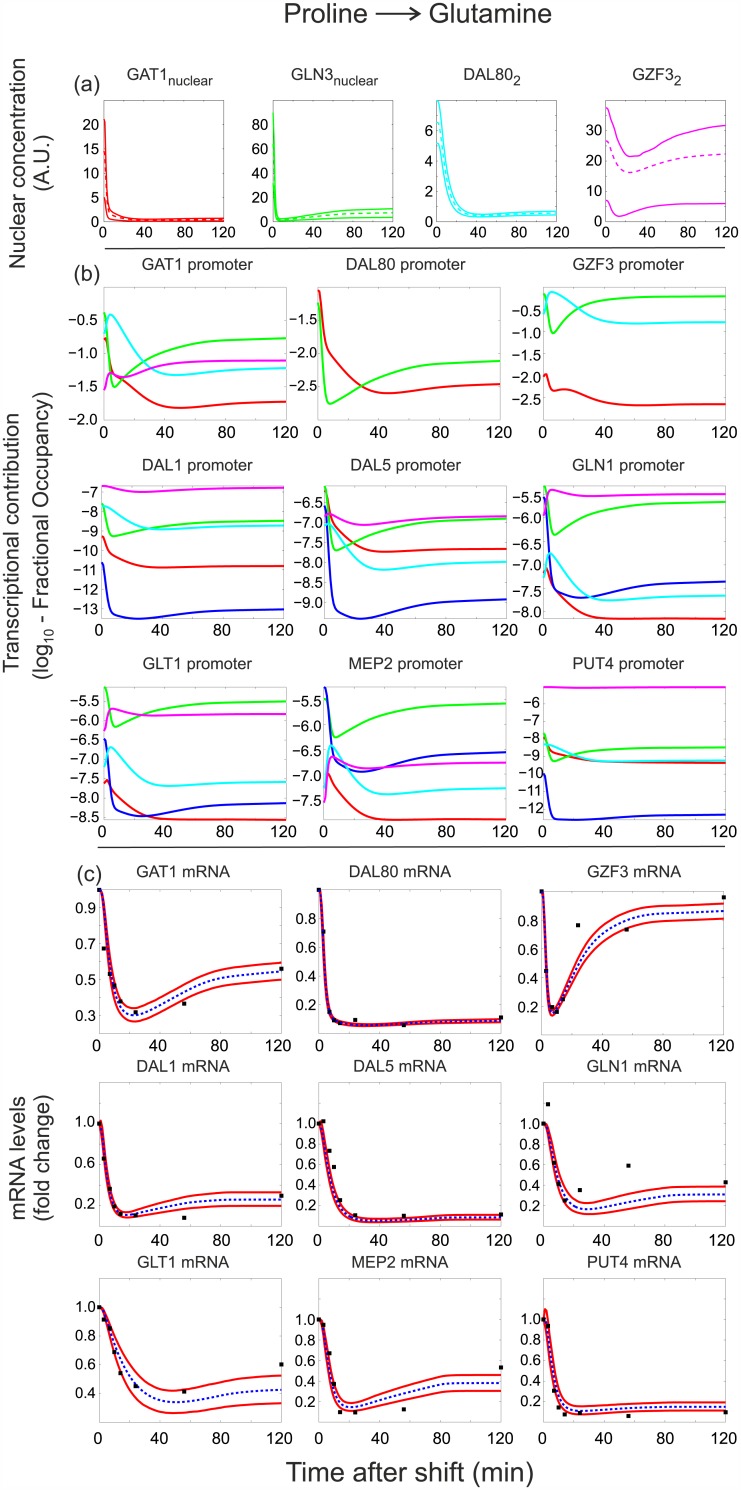
Model $M_{135}$ predictions and fits to target mRNA data (fold changes) for the Pro→Gln shift. (a) Abundance of GATA factor active forms (nuclear Gln3 and Gat1, and Dal80 and Gzf3 homodimers). In the case of Gln3 and Gat1, the observed decrease is accompanied by an increase in cytosolic concentration (not shown). The decrease of Dal80 is due to protein degradation/dilution as its expression is turned off. (b) Contribution of each GATA factor active form to the fractional occupancy of its target promoters, calculated as described in Section 2.2 of S1 Text. TFs with the greatest relative contribution to the fractional occupancy of a given target promoter at a given time are those that mainly control the expression of the corresponding target gene at that time. (c) Fits of the mRNA abundance of GATA targets. In parts (a) and (c), upper and lower continuous lines denote 90% and 10% quantiles respectively, based on the 500 parameter estimates with the highest posterior probability. In part (c), dashed lines correspond to the mean prediction and square markers denote measurements. In part (b), predictions are based on the maximum a posteriori (MAP) parameter estimate. TF contributions are color-coded according to part (a) (blue denotes the joint regulation by Gln3 and Gat1), and are given in logarithmic (base 10) scale due to their wide dynamic range.

**Fig 7:**
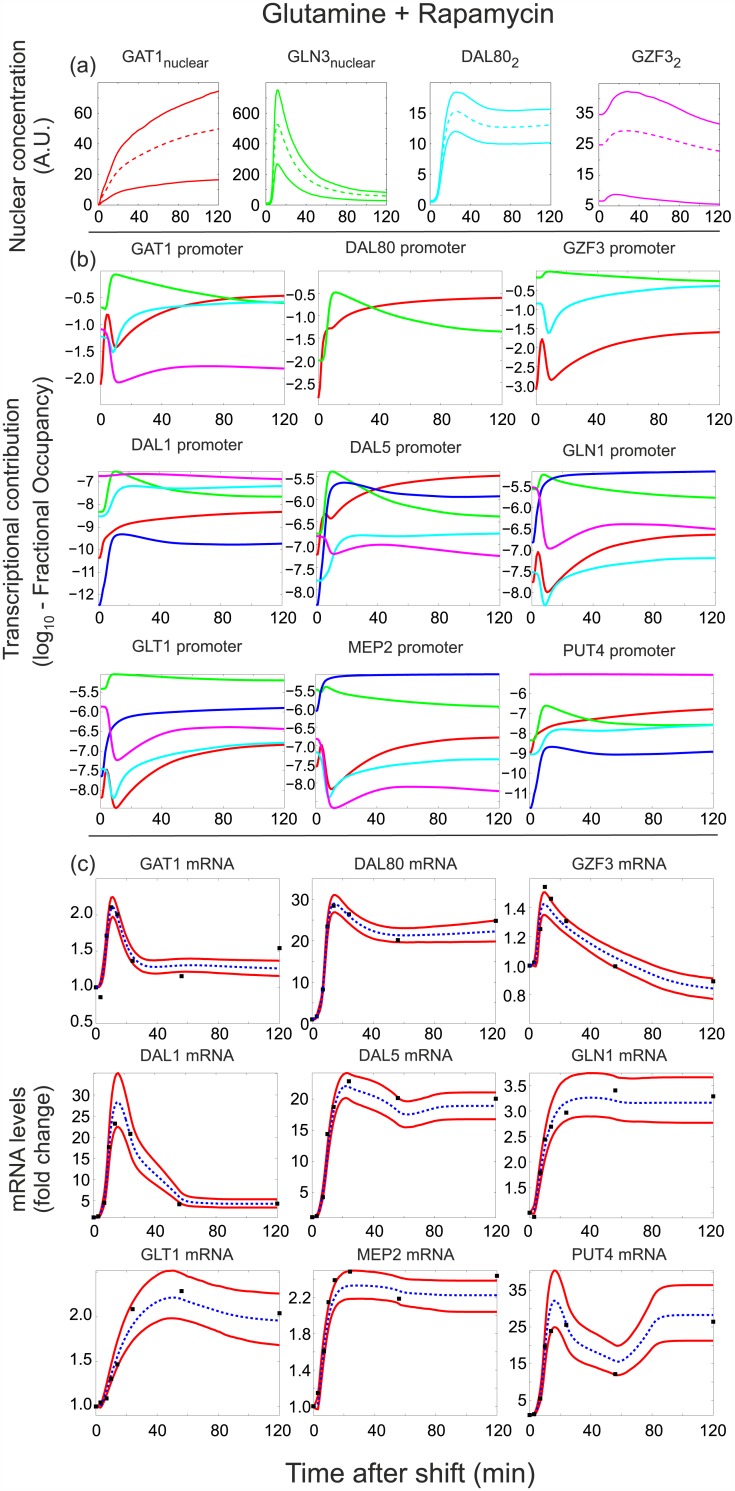
Model $M_{135}$ predictions and fits to target mRNA data (fold changes) for the Gln+Rap shift. Panel description is identical to Fig 6.

To gain further insights into open questions regarding the functioning of the GATA network, we next explored in detail the dynamic behavior of model $M_{135}$ to extract key quantitative variables that describe the dynamics of the GATA regulatory interactions during the nutritional upshift and the rapamycin-induced downshift (Figs 6 and 7).

The most obvious output of the model is its ability to describe the mRNA levels of the GATA factor targets, for which the model has been fitted during model selection. Figs 6(c) and 7(c) depict the experimental and described mRNA trajectories during the upshift and rapamycin treatment, respectively. Key open questions that remain elusive are (i) what are the dynamics of nuclear translocation/degradation of the GATA factors and how does that dictate their nuclear abundance, (ii) what is the nuclear abundance of each GATA factor and how does that dictate their TF activity, and (iii) which GATA factor is mainly responsible for the regulation of each target promoter. To address these questions, we extracted the model variables on the concentrations of nuclear and cytoplasmic GATA factor species and used them to calculate (i) the abundance of the active forms of Gat1, Gln3, Dal80 and Gzf3 (that is, nuclear Gln3 and Gat1, as well as Dal80 and Gzf3 homodimers, shown on Figs 6(a) and 7(a)), and (ii) the relative contribution of each of the four TF active forms to the regulation of the target gene expression (Figs 6(b) and 7(b)).

The inferred abundances for the active forms of Gat1, Gln3 and Dal80 show a drastic reduction within the first minutes upon glutamine addition to proline-grown yeast (Fig 6(a)). The nuclear depletion of Gat1 and Gln3, caused by their translocation to the cytosol as defined by the model, is completed within 5 minutes, while the nuclear depletion of Dal80, due to protein degradation after the shut-down of its expression, has a longer half-life of ∼15 minutes. The drastic depletion of Dal80 dimers is consistent with the fact that under nitrogen-rich conditions it is practically undetectable [30]. By contrast, nuclear abundance dynamics in the rapamycin-induced downshift reveal a clear difference between Gln3 and Gat1 (Fig 7(a)). While Gat1 increases its nuclear abundance monotonically to a saturation level after rapamycin treatment, Gln3 shows a transient overshoot to a lower steady state level. A similar trend has been observed experimentally, albeit with very coarse quantification and sparse sampling over time [9]. The abundance of Gzf3 remains practically constant in both shifts, as Gzf3 responds weakly and returns to steady-state levels after a transient change during the first 30 minutes of the shifts (Figs 6(a) and 7(a)).

To determine the relative contribution of each of the four TF active forms to the regulation of the target gene expression, we estimated the contribution of each TF to the fractional occupancy of each target gene promoter (Figs 6(b) and 7(b), Section 2.2 of S1 Text). The relative contributions of the GATA TFs to their target promoters during the upshift suggest that all target genes reduce their expression mainly because of the nuclear exit of the activators Gln3 and Gat1 (Fig 6, in particular panel b). The particular behavior of the Gzf3 mRNA (Fig 6(c)) seems however to arise from the interplay between Gln3 and the repressor Dal80: as Gln3 exits the nucleus and Dal80 remains around 15 minutes longer, *GZF3* expression transiently drops repressed by Dal80. Upon disappearance of Dal80, the repression effect disappears, and the basal expression of *GZF3* together with the small amount of nuclear Gln3 take over and restore the Gzf3 mRNA level. In contrast, the different nuclear behavior of the two activators in the rapamycin-induced downshift is reflected in the more diverse gene expression patterns of targets (Fig 7): those that are predicted by the model to be jointly regulated by Gln3 and Gat1 (e.g. *DAL80*, *DAL5*, *GLN1*, *GLT1*, *MEP2*, *PUT4*) according to the results of Fig 7(b), maintain a high expression level after the shift, while those affected mostly by Gln3 (e.g. *DAL1*, *GAT1*, *GZF3*) show a burst of expression followed by a lower steady state level. Interestingly, the latter group of genes also shows a high contribution of Dal80 in the later downregulation phase, which confirms the role of Dal80 as an important modulator of nitrogen catabolite repression relief [1]. Regardless of the condition, Figs 6(a) and 7(a) show that the role of Gzf3 in target expression seems to be that of a constant repressor, acting almost independently of the nitrogen source, possibly to assure full repression even in the presence of traces of nuclear Gln3 and Gat1 [1, 31].

## Discussion

Determination of functional gene regulatory interactions using currently available experimental techniques is still a time-consuming and non-trivial process, particularly difficult to resolve in networks containing feedback and/or feedforward loops. The yeast GATA gene regulatory network, the central transcriptional controller of nitrogen catabolite repression (NCR) in *S. cerevisiae*, is an example of a relatively well-characterized network with only four TFs but comprising several feedback/feedforward loops, which have so far hindered conclusive validation of several hypothesized interactions. In this work, we tackled the problem of identifying the most plausible interactions from existing hypotheses by applying mathematical modeling and Bayesian model selection to determine the support that experimental data lends to five yet unverified interactions within the GATA network. Overall, our model selection results provided strong evidence in favor of two of the hypothesized interactions, Gat1 self-activation and *GZF3* repression by Dal80 (hypotheses 2 and 4 on Fig 1), while further biological evidence is necessary to conclude on the requirement of Dal80 self-repression (hypothesis 1). The remaining hypotheses—*DAL80* repression by Gzf3 and Gln3-Gat1 interaction—appear dispensable according to our model, either because they are too weak to have significant impact on the measured system variables, or because they arose due to indirect regulatory effects.

Our approach relied on two rounds of Bayesian model selection applied to a system of ordinary differential equations describing the mechanistic details of transcription, translation and translocation of the members of the yeast GATA network. A basic model structure was first developed based on the current established regulatory interactions, and subsequently augmented to 32 structures corresponding to all possible topologies determined by combinations of the five hypothesized interactions. At this point we should note that our model selection approach (that is, considering the model structure corresponding to each combination of hypotheses in isolation) is equivalent to including a mass at zero in the priors of the full model that correspond to parameters that are “switched off” when certain interactions are missing and inferring the posterior parameter distribution over this complex multimodal prior. Further details are provided in Subsection 3.1.1 of S1 Text. Evaluation of the model structure that best described the dynamic mRNA data experimentally obtained in two distinct perturbations was enabled by a careful design of a computational pipeline that allowed us to efficiently handle models of great size and complexity, and which can prove to be generally useful for model-based inference problems with similar features. To overcome the great difficulties of sampling from complex, high-dimensional parameter distributions, particularly important here was the efficient design of our SMC sampler and our modular sampling approach that enabled the reduction of a high-dimensional sampling problem into two easier sub-problems. The applied Bayesian model selection procedure allowed us to identify a top-ranking model structure, $M_{135}$, that was able to reproduce the experimental data with the minimal necessary complexity, as well as to predict responses from an independent validation experiment.

The top-ranking model structure strongly supported the regulatory relevance of Gat1 self-activation and *GZF3* repression by Dal80, while the remaining three hypotheses did not substantially improve predictions relative to the basic model (Fig 3). When challenged to predict the outcome of a validation experiment comprising the GFP screening of each GATA-factor promoter activity in the absence of each of the regulators during the same two shifts, the top-ranking model performed well and qualitatively predicted the responses and sequence of events (Figs 4 and 5). Adding to its interest for model validation, the performed experiment offers a valuable dataset to systematically evaluate how each GATA-factor impacts each other’s gene expression during either an upshift or a downshift in NCR activity.

Many aspects of the functioning of the GATA network under NCR-repressive (glutamine-grown yeast) or NCR-relieved (proline-grown yeast) conditions can be confirmed simply based on the initial steady-state points of the validation experiments (initial points in Figs 4(a), 4(c) and 5(a), 5(c)), and can be better understood in light of the model structure. During exponential growth in glutamine, *DAL80* is derepressed in Δ*gzf*3, while *GZF3* is derepressed in Δ*gat*1 and repressed in Δ*gln*3. During growth in proline *DAL80* is derepressed in Δ*dal*80 and repressed in Δ*gln*3, while *GZF3* is derepressed in Δ*gat*1 and Δ*dal*80, and repressed in Δ*gzf*3. These observations generally agree with the established and here suggested regulatory interactions controlling *DAL80* and *GZF3* gene expression, as depicted on Fig 1. We noticed however that two of the observed results corresponded to hypotheses that were not validated by the top-ranking model: repression of *DAL80* by Gzf3 and self-repression of Dal80. While the latter needs further biological validation (it was part of the second-ranked model), our results suggest that the apparent repression of *DAL80* by Gzf3 is mediated through *GAT1*. Consequently, the increase of *DAL80* transcript levels in a Δ*gzf*3 strain is attributed to the relief of repression on *GAT1*, which in turn activates *DAL80*. Another apparent contradiction was the derepression of *GZF3* in Δ*gat*1, an unexpected behavior considering that Gat1 is an activator, and which contrasts with the result for Gln3, the other activator. This counterintuitive behavior is however predicted by the model (Fig 5(b) and 5(d)): Gat1 deletion leads to *DAL80* downregulation, which in turn causes an increase of Gzf3, since Dal80 is a direct inhibitor of *GZF3* expression (hypothesis 4). This contradiction further suggests that Gln3 is the main activator of *GZF3*, since only deletion of Gln3 (but not Gat1) lowers *GZF3* transcription. Also unexpected was the experimental observation that *GZF3* levels are repressed in Δ*gzf*3, an observation also explained by the model: when Gzf3 is deleted, *GAT1* expression increases and, due to the relatively weak effect of Gat1 on *GZF3*, the concomitant increase of Dal80 ultimately reduces the transcription of *GZF3*. As a final observation, we noticed from our experiments that Gzf3 mainly exerts its repressor activity specifically under NCR-repressive conditions, while it gets overshadowed by Dal80 once NCR is relieved, in agreement with previous reports from the literature [15, 32]. In fact, deletion of Dal80 did not result in a behavior different from the wildtype in glutamine-grown cells, supporting the view that *DAL80* is tightly switched off under NCR. Overall, the experimental data reflected well the current knowledge of the GATA-network in regulating NCR, and could offer several model-guided insights.

In addition to explaining experimental observations and helping to resolve the plausibility of the five hypothesized interactions, the top-ranking model structure was also explored to bring insights into the dynamics and operation of the yeast GATA network. To this end, we extracted from the model the variables that described the concentrations of nuclear/cytoplasmic TFs, and the relative contribution of each active TF to regulation of the different target gene expression (Figs 6 and 7). Our results regarding the differing nuclear localization responses of the two activators in the downshift agree with recent experimental observations suggesting that the nuclear localization of the GATA activators is likely to be regulated by two distinct pathways, of which one is more responsive to rapamycin, and the other to nitrogen source quality [33–36]. One particularly difficult question to resolve experimentally is the determination of the relative contribution of each GATA-factor to the regulation of their targets, since all GATA-factors share the same (or very similar) binding motifs on the promoter of the targets. By extracting from the model the fractional occupancy of each TF on each target gene (Figs 6(b) and 7(b)), we produced plausible predictions for the main responsible GATA-factor regulating each of the GATA targets considered in this study.

Altogether, our modeling exercise brought several insights into the function of the GATA network. First, the presence of Gat1 self-activation appears to confer greater independence from the other activator, Gln3, as suggested by the high levels of nuclear Gat1 following the rapamycin-induced downshift, when Gln3 is predominantly cytoplasmic (Fig 7(a)). Such independence seems to offer more fine tuning possibilities for yeast cells to regulate the balance between activators and repressors in the nucleus. Second, we provide strong evidence that Dal80 is indispensable to negatively regulate *GZF3*, and that this is not constitutively expressed as previously suggested by some groups [1, 31, 32], though contradicted by others [15, 16]. In fact, the experimentally measured Gzf3 mRNA clearly showed that *GZF3* is transiently regulated following the perturbations, before returning to a steady-state similar to initial levels. This transcriptional regulation however does not lead to great changes in abundance of Gfz3, rather suggesting that Gzf3 behaves like a constant repressor.

In conclusion, our work constitutes a necessary and important step in the direction of mathematical modeling of the yeast GATA gene regulatory network, a small system with a complex interaction pattern that has hampered clear interpretation of experimental observations related to NCR. Further accumulation of experimental data will enable our model to be expanded and connected with existing signaling models of the TOR pathway [37], nitrogen transport [38] and core metabolism [39], to gain a more holistic view and a better understanding of NCR.

## Materials and Methods

### Modeling assumptions

The GATA system equations (Section 2, S1 Text) are based on several assumptions supported by the literature and listed below for completeness:

#### 1. Mechanisms of GATA factor activation

we model the active forms of the repressors Dal80 and Gzf3 as homodimers [40] and of the activators Gln3 and Gat1 as monomers [31]. We did not include alternative complex compositions or protein-DNA interactions proposed in literature, such as Gat1-Gzf3 [31], Gln3-Dal80 [40], Gln3-Gzf3 [40] and Dal80-Gzf3 heterodimers [40] and Gzf3 monomer repression [32] because evidence for their functional role is scarce. Dal80 and Gzf3 are only nuclear [1], while Gln3 and Gat1 can be both nuclear (active) and cytoplasmic (both active and inactive).

**Fig 8:**
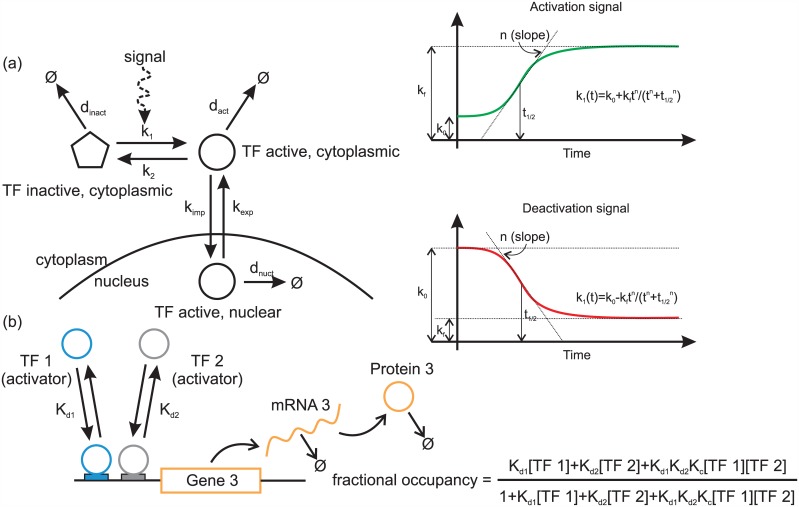
Summary of key modeling assumptions. (a) Assumed TF activation and translocation mechanism.(b) A small example of transcriptional regulation and gene expression modeling. The promoter of Gene 3 has two non-overlapping binding sites, where activators TF1 and TF2 can bind. The fractional occupancy of the promoter can be derived assuming fast equilibrium of binding and unbinding [43]. The factor *K*_*c*_ models the cooperative (when >1) or competitive (when <1) interaction of TF1 and TF2 in the case TF1 and TF2 are able to bind the promoter simultaneously [44].

The localization of Gln3 and Gat1 is controlled by cytoplasmic sequestration that in turn depends on their phosphorylation by upstream signaling pathways [41]. These (in)activation signals are considered as inputs to our system and are modeled using two individual functions that describe the monotonic, reversible activation and nuclear translocation of the two activators (Fig 8). The parameters governing the signal dynamics are learnt from the data and are the only ones to vary from one shift to the other, thus describing the external, unobservable upstream activation of the GATA factors. Besides upstream signaling activity, we consider Gln3 mRNA direct measurements (Fig. A in S1 Text) as the only additional input to our model, and further assume that mRNA changes are reflected in the abundance of Gln3 protein. This assumption is based on the observation that Gln3 mRNA displays moderate fluctuations during both shifts considered and these changes are weakly controlled by proteins other than the GATA factors (Gcn4 could be implicated in this regulation [42]). Apart from Gln3 gene expression, we assume that all other instances of gene expression regulation in the GATA network arise from within the network itself and thus are described in the model. While GATA factors have many confirmed and potential external regulators according to the Yeastract database (http://www.yeastract.org), none of these regulators changes significantly during the nitrogen source, or rapamycin shifts [11, 42].

#### 2. Mechanisms of gene regulation by GATA factors

Transcription factor binding is assumed to be non-competitive, i.e. several TFs are assumed to be able to bind to the same promoter simultaneously. This assumption is reflected in the form of the fractional occupancy functions for each GATA factors promoter. The plethora of GATA binding sites both on GATA-factor and target promoters indicates that this simplification is realistic. Moreover, testing all possible binding configurations for each promoter would be infeasible computationally

Additionally, the system is assumed to be at steady-state prior to each shift, an assumption supported the exponential growth of yeast cultures prior to each perturbation. What distinguishes the assumptions described above from the hypothesized alternative interactions evaluated in this work is the fact that former are all in agreement with our current biological knowledge of the GATA network [1–4, 41], while the latter have been determined based on contradictory and/or ambiguous experimental data that provide equally plausible evidence for their presence and absence.

### Modeling of the GATA network

All GATA factors recognize the same core motif (5’-GATAA-3’ or 5’-GATTA-3’), found in several copies upstream of NCR-controlled targets, as well as at the *GAT1*, *DAL80* and *GZF3* promoters. Gln3 is the only GATA factor whose expression is not nitrogen-regulated to any significant extent [1], while the rest of the GATA factors display a complex interaction pattern ([1, 2, 10, 16, 45] and references therein). From the interactions summarized in Figs 1 and 8, the following chemical reactions were derived, based on the list of assumptions given above (proteins are denoted by capital first letter, mRNA by small):

**Transcription factor activation and translocation**

$$\begin{array}{c} {\text{Gln3}}_{\text{inactive}}^{\text{cyt}}\overset{k_{2w}}{\underset{k_{1w}(t)}{⇌}}{\text{Gln3}}_{\text{active}}^{\text{cyt}}\\ {\text{Gat1}}_{\text{inactive}}^{\text{cyt}}\overset{k_{2x}}{\underset{k_{1x}(t)}{⇌}}{\text{Gat1}}_{\text{active}}^{\text{cyt}}\\ {\text{Gln3}}_{\text{active}}^{\text{cyt}}\overset{k_{exp}^{W}}{\underset{k_{imp}^{W}}{⇌}}{\text{Gln3}}^{\text{nuc}}\\ {\text{Gat1}}_{\text{active}}^{\text{cyt}}\overset{k_{exp}^{X}}{\underset{k_{imp}^{X}}{⇌}}{\text{Gat1}}^{\text{nuc}} \end{array}$$

**mRNA production/degradation**

$$\begin{array}{c} \begin{array}{c} ⊘\overset{Gln3^{nuc},Gat1^{nuc},Dal80_{2},Gzf3_{2}}{\to }gat1\\ ⊘\overset{Gln3^{nuc},Gat1^{nuc},Dal80_{2},Gzf3_{2}}{\to }dal80\\ ⊘\overset{Gln3^{nuc},Gat1^{nuc},Dal80_{2}}{\to }gzf3\\ gat1\overset{k_{dx}}{\to }⊘\\ dal80\overset{k_{dy}}{\to }⊘\\ gzf3\overset{k_{dz}}{\to }⊘ \end{array} \end{array}$$

**Protein production/degradation**

$$\begin{array}{c} \begin{array}{c} gln3\overset{k_{pW}}{\to }gln3+\text{Gln}3_{\text{inactive}}^{\text{cyt}}\\ gat1\overset{k_{pX}}{\to }gat1+\text{Gat}1_{\text{inactive}}^{\text{cyt}}\\ dal80\overset{k_{pY}}{\to }dal80+\text{Dal}80\\ gzf3\overset{k_{pZ}}{\to }gzf3+\text{Gzf}3\\ \text{Gln}3_{\text{inactive}}^{\text{cyt}}\overset{k_{dWi}}{\to }⊘\\ \text{Gat}1_{\text{inactive}}^{\text{cyt}}\overset{k_{dXi}}{\to }⊘\\ \text{Gln}3_{\text{active}}^{\text{cyt}}\overset{k_{dW}}{\to }⊘\\ \text{Gat}1_{\text{active}}^{\text{cyt}}\overset{k_{dX}}{\to }⊘\\ \text{Gln}3^{\text{nuc}}\overset{k_{dW}}{\to }⊘\\ \text{Gat}1^{\text{nuc}}\overset{k_{dX}}{\to }⊘\\ \text{Gzf}3\overset{k_{dZ}}{\to }⊘\\ \text{Dal}80\overset{k_{dY}}{\to }⊘\\ {\text{Gzf}3}_{2}\overset{k_{dZ_{2}}}{\to }⊘\\ {\text{Dal}80}_{2}\overset{k_{dY_{2}}}{\to }⊘ \end{array} \end{array}$$

**Protein-protein interactions**

$$\begin{array}{c} \text{Gzf3}+\text{Gzf3}\overset{k_{Z_{2}}}{\underset{k_{Z_{2}}^{diss}}{⇌}}{\text{Gzf3}}_{2}\\ \text{Dal80}+\text{Dal80}\overset{k_{Y_{2}}}{\underset{k_{Y_{2}}^{diss}}{⇌}}{\text{Dal80}}_{2} \end{array}$$

The above reactions are described by a set of ordinary differential equations given in Section 2 of S1 Text. They are all assumed to follow mass-action kinetics, except mRNA transcription and TF activation. The role of each regulator on the production rate of a given mRNA is clarified in Fig 1. The transcription rate of a specific mRNA is assumed to be proportional to the fractional occupancy of its promoter, i.e. the fraction of time that the promoter is active. The fractional occupancy at any given time is a function of the regulator amounts present at that time (following the common quasi-steady-state assumption for promoter occupancy). The form of the fractional occupancy function is determined using the thermodynamic approach of [43, 46]. An example of a fractional occupancy function for two activators is given on Fig 8.

Depending on the type of shift modeled (i.e. upshift or downshift) a separate activation/inactivation signal from the upstream signaling components is considered for each activator, and serves as an external input to the system (functions *k*_1*w*_(*t*) and *k*_1*x*_(*t*) in the reactions above). Each signal belongs to a class of sigmoid functions, which is biologically plausible and can capture step-like activity changes. The parameters of our sigmoids have to be estimated from the available transcription data, along with the rest of system parameters. More concretely, the parameterized functional forms we assume, also displayed on Fig 8, are the following:

- TF activation: $k_{1}\left(t,V_{0},V,n,\theta \right)=V_{0}+V\frac{t^{n}}{t^{n}+\theta^{n}}$ (following rapamycin treatment)
- TF inactivation: $k_{1}\left(t,V_{0},V,n,\theta \right)=V_{0}(1-V\frac{t^{n}}{t^{n}+\theta^{n}})$ (following a shift from proline to glutamine)

The role of each parameter in the above functions is intuitively obvious. Each GATA activator is assigned its own set of parameter values, which also vary between the different shifts and have to be estimated from the available transcription data, along with the rest of system parameters. The assumed time dependence of the activation rate is reasonable, given recent experimental readouts of TOR pathway activity, which show **a)** a fast, step-like decrease in TOR activity upon rapamycin treatment [47] **b)** a very fast, step-like increase in TOR activity during a nutrient upshift (proline to glutamine) [47] **c)** a very fast, step-like increase in Gln3 phosphorylation (which controls its cytoplasmic localization) upon a nutrient upshift (proline to glutamine) [8].

Finally, to obtain the Gln3 mRNA input signal the available mRNA timecourse measurements for each experiment (Section 2.8 and Fig. A in S1 Text) were linearly interpolated and fed into the model simulator.

### Implementation of Bayesian model selection

The generated ordinary differential equation models encode mathematically the existing biological knowledge about the GATA network and enable us to use statistical methods for selecting the model with the optimal complexity that can reproduce the available experimental data. In this work we chose to carry out model selection in a Bayesian framework [48]. Contrary to the commonly used Akaike and Bayesian Information Criteria (AIC and BIC), which are valid only asymptotically [49] (i.e. as the amount of data tends to infinity), Bayesian model selection is applicable with a limited amount of data. Moreover, it naturally penalizes model complexity without explicitly referring to the number of model parameters, as AIC and BIC do. This is especially important for large nonlinear models considered in Systems Biology, as practical unidentifiability of parameters [50] is very common and implies that the “effective” number of parameters (“degrees of freedom”) in a given model does not correspond to the actual number of parameters. Finally, Bayesian model selection incorporates our prior beliefs about parameter values and model plausibility in a consistent way, whereas this is impossible with AIC and BIC.

Given a set of competing biological hypotheses ${\left\{H_{k}\right\}}_{k=1}^{K}$, each encoded in a mathematical model $M_{k}$, Bayesian model selection works by computing the posterior probability $P(M_{k}|D)$ of each model given the available experimental data *D*. This involves the computation of the marginal likelihood (also called evidence) $P(D|M_{k})$, which, being an integral over the high-dimensional parameter space of $M_{k}$, forms the main computational bottleneck of the process. Further details on Bayesian model selection are provided in Section 3.1 of S1 Text.

Since the evidence $P(D|M_{k})$ cannot be evaluated analytically in all but the simplest cases, Monte Carlo-based numerical integration methods are typically employed for its computation. Due to the high dimensionality of the parameter spaces considered, simple estimators based on the Laplace approximation of the posterior and importance sampling estimators have been shown to result in highly variable and/or biased results [51]. After a detailed comparison of different sophisticated sampling methods [24], we chose to implement a Sequential Monte Carlo (SMC) sampler, described in more detail in Section 3.2 of S1 Text.

Briefly, the SMC sampler can provide samples from the posterior distribution of parameter values, $P(\theta_{k}|D,M_{k})$ (where *θ*_*k*_ denotes the parameter vector of the *k*-th model), as well as an estimate of the evidence integral. $P(\theta_{k}|D,M_{k})$ expresses the conditional distribution of the model parameters after taking the observed dataset *D* into account [48] and, according to Bayes’ theorem, it is proportional to $P\left(D|M_{k},\theta_{k}\right)P\left(\theta_{k}|M_{k}\right)$, where is $P(D|M_{k},\theta_{k})$ the likelihood function and $P(\theta_{k}|M_{k})$ the prior parameter distribution (definitions and details are provided in Section 3.1 of S1 Text).

SMC generates samples from the posterior parameter distribution and estimates the evidence using a sequence of bridging distributions, *f*_*β*_, defined according to a “cooling schedule”:

$$\begin{array}{c} f_{\beta_{i}}{\left(\theta )\propto P(D|M,\theta \right)}^{\beta_{i}}P\left(\theta |M\right), \end{array}$$ (1)

for 0 = *β*_0_ < *β*_1_ < … < *β*_*N*_ = 1. The algorithm works by propagating a population of particles sampled from the diffuse prior through this sequence of intermediate distributions that gradually “morph” into the (typically much more concentrated and complex) target posterior.

As it is practically impossible to verify SMC convergence in a rigorous way for the problem at hand, we repeatedly ran the algorithm for a few different models to monitor the variability of the estimated quantities and detect any anomalous behavior. The algorithm was thus iteratively tuned so that the variance of the estimates was small enough to permit safe conclusions about model ranking (further details can be found in Section 5.2 of S1 Text).

### Evidence decomposition for modular systems

When the dynamical system of interest displays a modular structure without feedbacks, a simple rewriting of the evidence integral can prove very helpful for carrying out the computation in a sequential manner. We have used this evidence decomposition to speed up the computation in the second model selection step by defining the transcription factor network as the “upstream” module, and the six GATA targets as the “downstream” modules, as described in Section 4.2 of S1 Text.

Here, we briefly describe the concept of evidence decomposition for modular systems: as an example, consider a dynamical system of the form

$$\begin{array}{c} \dot{x}=F\left(x,\theta \right), \end{array}$$

where $x\in R^{n}$ and $\theta \in R^{m}$ is the parameter vector. We make the following assumptions:

1. There are two disjoint groups of states and parameters, **x**_1_ and **x**_2_ (*θ*_1_ and *θ*_2_), such that
  $$\begin{array}{c} F\left(x_{1},x_{2},\theta_{1},\theta_{2}\right)=\left[\begin{array}{c} F_{1}\left(x_{1},\theta_{1}\right)\\ F_{2}\left(x_{1},x_{2},\theta_{2}\right) \end{array}\right], \end{array}$$
  i.e. the first group of states affects the second, but not vice versa. Thus, the system can be decomposed into two subsystems, with the first affecting the second.
2. We have timecourse measurements of (some of) the states in **x**_1_, denoted collectively by *D*_1_, as well as measurements of some **x**_2_ states, denoted by *D*_2_.

If we denote by *π*(*θ*_1_) and *π*(*θ*_2_) the priors on the two parameter sets and by *P*(*D*_1_, *D*_2_|*θ*_1_, *θ*_2_) the likelihood function of the parameters, we can immediately write

$$\begin{array}{c} P\left(D_{1},D_{2}|\theta_{1},\theta_{2}\right)=P\left(D_{1}|\theta_{1}\right)P\left(D_{2}|\theta_{1},\theta_{2}\right). \end{array}$$ (2)

The form of the likelihood thus encodes the flow of state information between the two subsystems, and can be easily generalized to the case of a cascade of *n* subsystems, each affecting the next.

In the simple case of two modules, the evidence integral becomes

$$\begin{array}{c} P\left(D_{1},D_{2}\right)=\;\int \int \;P\left(D_{1}|\theta_{1}\right)P\left(D_{2}|\theta_{1},\theta_{2}\right)\pi \left(\theta_{1}\right)\pi \left(\theta_{2}\right)d\theta_{1}d\theta_{2} \end{array}$$ (3)

$$\begin{array}{c} =\underset{P(D_{1})}{\underset{︸}{\int \;P\left(D_{1}|\theta_{1}\right)\pi \left(\theta_{1}\right)d\theta_{1}}}\int \;\frac{P\left(D_{1}|\theta_{1}\right)\pi \left(\theta_{1}\right)}{\int P\left(D_{1}|\theta_{1}\right)\pi \left(\theta_{1}\right)d\theta_{1}}P\left(D_{2}|\theta_{1},\theta_{2}\right)\pi \left(\theta_{2}\right)d\theta_{2} \end{array}$$ (4)

$$\begin{array}{c} =P\left(D_{1}\right)\int \;P\left(D_{2}|\theta_{1},\theta_{2}\right)P\left(\theta_{1}|D_{1}\right)\pi \left(\theta_{2}\right)d\theta_{2}. \end{array}$$ (5)

In the above equations, *P*(*D*_1_) denotes the evidence of the module corresponding to *F*_1_, based only on the *D*_1_ dataset by ignoring the downstream subsystem. Apart from *P*(*D*_1_), we also need *P*(*θ*_1_|*D*_1_), which is the parameter posterior for the upstream module, based again on *D*_1_. According to this rewriting of the total evidence, its calculation can then proceed in two steps: first, the upstream module is treated in isolation, and the results of this computation (evidence and parameter posterior) are then fed into the calculation of the evidence for the downstream module. In effect, numerical estimation of this second integral amounts to integrating the likelihood for *D*_2_ with respect to the posterior of *θ*_1_ in place of the prior, and multiplying by the evidence *P*(*D*_1_).

The same procedure can be generalized when multiple subsystems are jointly affected by the first one, *but do not interact with each other*. Further details on how this decomposition can be exploited in the SMC sampling algorithm are provided in Section 4 of S1 Text.

### Implementation and computational cost analysis

All models were implemented using SBTOOLBOX2 [52] (http://www.sbtoolbox2.org/main.php), a freely available Matlab toolbox that is best suited for simulation and analysis of ODE-based models. The SBPD extension of the toolbox is particularly useful, as it enables high-speed simulation (∼100x faster than the built-in Matlab integrators) of high-dimensional ODEs by converting models to C code and using the powerful CVODEs integrator [53] from the SUNDIALS package [54].

At each temperature step, the SMC sampler requires the likelihood evaluation of *b* ⋅ *M* parameter points, where *M* is the size of the particle population and *b* the number of Metropolis-Hastings iterations used in our proposal kernel (Section 3.4, S1 Text). Since the likelihood evaluation requires the integration of the model ODEs, this is a very computationally demanding task, even if a single model run takes a small fraction of a second. For this reason, all SMC runs in this work were performed on 64 cores of the ETH Brutus cluster (https://www1.ethz.ch/id/services/list/comp_zentral/cluster/index_EN), using custom-written and speed-optimized parallel Matlab code. With this setup, an SMC run of the first model selection round with *M* = 15000, *b* = 15 and 70 temperature steps, takes around 2 hours to complete for each model structure. Additional speedup can be achieved by converting into C code the second most time-consuming step of the SMC, the fit of the Gaussian mixture model (Section 3.4, S1 Text).

The full GATA-factor model in SBML and SBTOOLBOX2 formats is provided in S1 File.

### Experimental data for model selection

We used time-course mRNA microarray data previously obtained by us in two different perturbation experiments: a nitrogen quality upshift from proline to glutamine (Pro→Gln) and a rapamycin-induced downshift during growth in glutamine (Gln+Rap) [22] (NCBI GEO accession numbers GSE54844 and GSE54851). Briefly, wildtype *Saccharomyces cerevisiae* was grown in well-controlled bioreactor operated in batch mode using a defined minimal media with glucose as sole carbon source and a defined nitrogen source composition. In the Pro→Gln upshift, yeast was grown exponentially in proline as sole nitrogen-source and a dynamic upshift was induced by addition of glutamine. In the rapamycin-induced downshift (Gln+Rap), the downshift was induced by the addition of rapamycin to yeast growing exponentially in glutamine. Gene expression was quantified using Affymetrix DNA microarrays at eight timepoints (-10, 3, 7, 10, 14, 24, 56 and 120 minutes after the perturbation), with triplicate measurements taken at -10, 7, and 24 minutes from three independent biological replicates. Further replicates are cost-prohibitive for such dynamic experiments [22]. The triplicates were used to assess both the biological and microarray variability and define a measurement noise model (S1 Text, Section 3.3). Since Affymetrix DNA microarrays do not allow comparison of intensities across different transcripts species, we worked with fold-changes normalized relative to the steady-state sample taken before the time of the shift. Experimental and data processing details can be found in [22].

### Experimental data for model verification

Wildtype *S. cerevisiae* FY4 and four isogenic single gene-deletion yeast strains lacking each of the four GATA-factors were transformed with the low-copy plasmid pRS41H harboring the promoter region of each GATA-factor (-600 to -1 bp upstream of the beginning of the ORF) immediately upstream of a GFP reporter gene (see S2 Text for details). Plasmid inserts containing the GATA promoter, the yGFP3 sequence and the yeast CDC28 terminator were synthesized by GeneArt AG (Regensburg, Germany) as described in S2 Text. This resulted in a total of 25 strains (five backgrounds—wildtype, Δ*dal*80, Δ*gat*1, Δ*gln*3 and Δ*gzf*3—each transformed with one of the possible five plasmids harboring the promoter GATA-GFP—empty vector, pDAL80-GFP; pGAT1-GFP, pGLN3-GFP and pGZF3-GFP). All strains were cultivated in microtiter plates in Biolector, grown under the same conditions and subjected to the same shifts used to generate the mRNA data (details in S2 Text). Cell fluorescence (GFP filter) and biomass accumulation was monitored in real time (S1 Dataset). The fluorescence (*I*(*t*)) and biomass (*A*(*t*)) measurements were background-corrected and processed following the approach described in [29] to obtain the relative concentration of GFP, *r*(*t*) ∝ *I*(*t*)/*A*(*t*), as well as the time-dependent growth rate *μ*(*t*) = *d*ln(*A*(*t*))/*dt*.

## Supporting Information

S1 Text Details on mathematical modeling and model selection. (PDF)

S2 Text Detailed experimental procedure. (PDF)

S1 Dataset GFP intensities and biomass evolution of all strains containing the pDAL80-GFP, pGZF3-GFP and pGLN3-GFP plasmids (raw and processed Biolector data). (ZIP)

S1 File The full GATA-factor model in SBML and SBTOOLBOX2 formats. (ZIP)
