# Supplementary material for: Elucidation of Genetic Interactions in the Yeast GATA-Factor Network Using Bayesian Model Selection
Source: PLoS Comput Biol. 2016 Mar 11;12(3):e1004784. doi: 10.1371/journal.pcbi.1004784 (PMC4788432; doi:10.1371/journal.pcbi.1004784)
Supplement: S1 Text — (PDF) [file pcbi.1004784.s001.pdf]

S1 Text. Details on mathematical modeling and model selection

Contents

1 Biological background **2**
- 1.1 Established interactions 2
- 1.2 Hypothesized interactions 2
- 1.3 Choice of target genes 3

2 ODE modeling of the GATA network **4**
- 2.1 Implementation of the steady-state assumption 4
- 2.2 Relative TF contributions to fractional occupancy 4
- 2.3 Transcription factor (full) model equations 4
- 2.4 Target model 5
- 2.5 GFP reporter model 6
  - 2.5.1 Interpreting the GFP reporter measurements 7
- 2.6 GATA model parameters 7
  - 2.6.1 Transcription factor model parameters 7
  - 2.6.2 Target model parameters 10
- 2.7 Parametrization of models corresponding to different hypotheses 10
- 2.8 SBML implementation 11
- 2.9 Details on modeling assumptions 11

3 Bayesian model selection and the SMC sampler for the GATA network **13**
- 3.1 Bayesian model selection 13
  - 3.1.1 Discriminating among multiple hypotheses using a single multimodal prior 14
- 3.2 Algorithm 14
- 3.3 Model-related settings 16
  - 3.3.1 Prior selection and parametrization 16
  - 3.3.2 Noise model 16
- 3.4 Sampler settings 18
  - 3.4.1 Cooling schedule 18
  - 3.4.2 Transition kernels 18
  - 3.4.3 Resampling strategy 19
  - 3.4.4 Population size 19

4 Sampling parameters for modular systems **19**
- 4.1 Parameter sampling for decomposed systems using SMC 20
- 4.2 Modular sampling for the GATA system 21

5 Parameter inference and model selection with SMC **22**
- 5.1 Example: parameter inference for the full model 22
- 5.2 Verification of SMC convergence and variability of estimates 24
  - 5.2.1 Sensitivity to the prior 26
  - 5.2.2 Random perturbations to the prior: 26
- 5.3 Posterior probability estimates (1st model selection round) 33
- 5.4 Multiplicative factor estimates 33
- 5.5 Broadening selected prior ranges 33

1 Biological background

1.1 Established interactions

The following table provides a non-exhaustive list of references for each of the interactions characterized as established in the main text. Note that GATA-factor names have varied in the past: *GAT1* has also been known as *NIL1*, *DAL80* as *UGA43* and *GZF3* as *DEH1* and *NIL2*.

Interaction description	Relevant references
Dal80 repression of *GAT1*	[13,14], [31] (main text)
Gat1 activation of *DAL80*	[13,14], [31] (main text)
Gln3 activation of *GAT1*	[10], [31] (main text)
Gln3 activation *GZF3*	[15], [16] (main text)
Gat1 activation of *GZF3*	[15], [16] (main text)
Dal80 repression of *GZF3*	[15], [16] (main text)
Gzf3 repression of *GAT1*	[32], [31] (main text)

1.2 Hypothesized interactions

An extensive literature search resulted in several interactions in the GATA network that remain unvalidated to date. They are briefly enumerated below and denoted with dashed lines on Figure 1 of the main text.

Interaction #	Description	Relevant references
1	*DAL80* self-repression	[14], [15], [16] (main text)
2	*GAT1* self-activation	[1], [10], [16], [31], [32] (main text)
3	Gzf3 repression of *DAL80*	[15], [16] (main text)
4	Dal80 repression of *GZF3*	[15], [16], [32] (main text)
5	Gln3-Gat1 interaction	[17], [31] (main text)

1.2.0.1 Discussion of experimental evidence for each interaction

1. It has been suggested that Dal80 exerts negative control on itself [14], [15], [16] (main text), but its binding on the *DAL80* promoter (suggested to be quite weak in [16] (main text)) has not been verified yet, while the importance of this interaction for the dynamic behavior of the system is unknown. Moreover, the interaction has been inferred by an increase in $DAL80$ promoter activity in a $\Delta dal80$ background, based on Northern blots [14] (main text) and LacZ assays [15] (main text). However, one cannot preclude the possibility that this observation is due to indirect regulatory interactions between Dal80 and the rest of the GATA factors.

2. There have been a few reports about the existence of an autoactivation loop on *GAT1*. While initially discovered in 1997 based on the observation that $GAT1$ is capable of self-activation in the absence of Gln3 in a $\Delta gzf3$ background using a LacZ assay [32] (main text), the interaction has disappeared [16] (main text) and reappeared [1] (main text), [10] (main text) in descriptions of the GATA network published in the following years. Interestingly, [16] (main text) reached the conclusion that Gat1 is not self-regulated by noting that deletion of $GAT1$ actually increased pGat1-LacZ activity compared to wild type in a proline-grown $\Delta gat1$ strain. It was later shown that Gat1 can bind to its own promoter [31] (main text), however the regulatory capacity and importance of this interaction is still unclear. Both observations, in favor or against the presence of the interaction, can be explained by indirect regulatory effects.

3. Same as Dal80 self-repression, the negative regulation of *DAL80* by Gzf3 has been shown using assays that cannot differentiate between direct and indirect effects [15], [16] (main text).

4. The evidence on *GZF3* regulation is the most contradictory. Several papers consider *GZF3* expression to be almost constitutive [32] (main text), while others demonstrate its dependence on Gat1 and Gln3 [16] (main text). There is also reference to strong negative regulation of *GZF3* by Dal80 [15], [16] (main text), but no further experiments have verified or disproved this interaction yet. While repression of *DAL80* by Gzf3 is thought to only be effective in a repressive medium (where the Dal80 protein has very low abundance), *GZF3* down-regulation by Dal80 must take place when Dal80 is active, i.e. in derepressive conditions.

5. Every NCR-sensitive promoter contains more than one GATA elements, which implies that the two GATA activators can be found simultaneously on the same promoter. Study of single and double deletions of Gln3 and Gat1 indicate the existence of cooperative behavior for some promoters [31] (main text). This has also been demonstrated in several cases where Gln3 binding efficiency on target (non-GATA) genes is greatly reduced in the absence of Gat1 [17]. The opposite phenomenon has also been observed, namely that Gln3 and Gat1 can compete for the same binding sites, either by mutual exclusion or by reducing the binding affinity of each other when bound to the same promoter [39]. Obtaining the same information for the GATA factor promoters is not an easy task, especially when using steady-state deletion data, hence no concrete evidence exists.

1.3 Choice of target genes

Given that all GATA factors recognize the same target sequences, and the fact that most NCR genes (including the GATA factors except Gln3) contain multiple GATA motifs in their promoters, it is reasonable to assume that all GATA TFs compete for the same binding sites [10], [16] (main text). However, not all target genes are regulated to the same extent by all GATA factors, since the presence or absence of auxiliary binding sites, and the number, spacing and flanking sequences of the GATA motifs play significant role in the control of each target promoter [36], [1], [27] (main text). The precise way in which all these elements affect GATA factor binding is still under investigation, and some targets have been studied in much more detail than others. A common characteristic of the target genes considered in this work is that they are known to be mainly controlled by the GATA factors during NCR.

- *DAL1* (allantoinase) and *DAL5* (allantoin permease): Both genes are verified GATA targets, with the latter often serving as a readout gene for GATA-factor activity (e.g. [17]). In addition, their expression is not induced by any intermediates of the allantoine pathway, as happens in the case of DAL4 and DAL7 [2] (main text).

- *GLN1* (glutamine synthetase) and *GLT1* (glutamate synthetase): Several pathways impinge on the expression of these genes. However, they are mainly regulated in response to changes in intracellular glutamine and glutamate levels respectively, that result from different available nitrogen sources. They also display a much simpler, mostly Gln3-dependent regulation pattern than other core nitrogen-metabolic enzymes, such as GDH1 and GDH2 [40], [2] (main text).

- *MEP2* (ammonium permease): From the three known ammonium permeases, Mep1, Mep2, and Mep3, the second displays the highest sensitivity to the nitrogen source (as well as the highest affinity for ammonium) and appears to be controlled by both GATA activators [40].

- *PUT4* (proline permease): The high-affinity permease has been included as a representative of the proline degradation pathway, which has long been established to be NCR-sensitive. In contrast to *PUT1* and *PUT2*, *PUT4* is not under the control of Put3, a transcription factor whose activity is induced by proline and is necessary for the full activation of *PUT1* and *PUT2* [2] (main text).

2 ODE modeling of the GATA network

2.1 Implementation of the steady-state assumption

All initial conditions are calculated based on the assumption that the system is at steady-state at the beginning of each shift. This implies that a system of nonlinear algebraic equations has to be solved for the given parameter set. Given the structure of the mRNA equations, obtaining analytical solutions is a formidable task. However, steady-state protein abundances are relatively easy to calculate, given a set of mRNA values. Hence, we follow a simple fixed-point iteration scheme to approximate the steady-state initial conditions of the model: starting from some initial mRNA abundance values, the corresponding protein abundances are calculated analytically, and the outcome of the calculation is fed back into the mRNA equations, which give a new set of mRNA abundances. The process is then repeated a few times until convergence.

2.2 Relative TF contributions to fractional occupancy

The relative contribution of each GATA factor to the fractional occupancy of a target promoter can be defined with the help of the small example presented on Figure 8 of the main paper: the relative contribution of TF1 is $K_{d1}[TF1] \cdot (1 + K_{d1}[TF1] + K_{d2}[TF2] + K_{d1}K_{d2}K_c[TF1][TF2])^{-1}$, while the joint contribution of TF1 and TF2 would be $K_{d1}K_{d2}K_c[TF1][TF2] \cdot (1+K_{d1}[TF1]+K_{d2}[TF2]+K_{d1}K_{d2}K_c[TF1][TF2])^{-1}$.

As a more general example, consider the *GAT1* promoter, whose fractional occupancy function is given in subsection 2.7 below. This promoter is regulated both by repressors and activators. The relative contribution of Gln3 is then defined as

$$\frac{k_{xw}[W]}{1 + k_{xw}[W] + k_{xx}[X] + k_{xw}k_{xx}k_{xwc}[W][X]} \cdot \frac{1}{1 + k_{xy}[Y_2] + k_{xz}[Z_2]},$$

while the relative contribution of Dal80, is given by

$$\frac{k_{xy}[Y_2]}{1 + k_{xw}[W] + k_{xx}[X] + k_{xw}k_{xx}k_{xwc}[W][X]} \cdot \frac{1}{1 + k_{xy}[Y_2] + k_{xz}[Z_2]}$$

Given the above definition, it becomes obvious that the TFs with the greatest relative contribution to the fractional occupancy of a given target promoter are those that mainly control the expression of the corresponding target gene.

2.3 Transcription factor (full) model equations

Model states comprise the abundances of Gat1, Dal80 and Gzf3 mRNA, the nuclear and cytoplasmic parts of Gln3 and Gat1 (further divided into active and inactive cytoplasmic parts), and the Dal80 and Gzf3 monomers and homodimers. Table A describes the notation for model variables (both states and inputs).

Using this notation, the model equations derived from the set of chemical reactions given above are the following:

mRNA dynamics:

$$\dot{[x]} = k_x \frac{k_{xw}[W] + k_{xx}[X] + k_{xw}k_{xx}k_{xwc}[W][X]}{1 + k_{xw}[W] + k_{xx}[X] + k_{xw}k_{xx}k_{xwc}[W][X]} \cdot \frac{1}{1 + k_{xy}[Y_2] + k_{xz}[Z_2]} - k_{dx}[x]$$

$$\dot{[y]} = k_y \frac{k_{yw}[W] + k_{yx}[X] + k_{yw}k_{yx}yk_{ywxc}[W][X]}{1 + k_{yw}[W] + k_{yx}[X] + k_{yw}k_{yx}k_{ywxc}[W][X]} \cdot \frac{1}{1 + k_{yy}[Y_2] + k_{yz}[Z_2]} - k_{dy}[y]$$

$$\dot{[z]} = k_z \frac{k_{zw}[W] + k_{zx}[X] + k_{zw}k_{zx}k_{zxwc}[W][X]}{1 + k_{zw}[W] + k_{zx}[X] + k_{zw}k_{zx}k_{zxwc}[W][X] + k_{zy}[Y_2]} - k_{dz}[z]$$

Variable name	Symbol
Gln3 (in)activation rate (input)	$k_{1w}(t)$
Gat1 (in)activation rate (input)	$k_{1x}(t)$
Gln3 mRNA (input)	$[w]$
Gat1 mRNA	$[x]$
Dal80 mRNA	$[y]$
Gzf3 mRNA	$[z]$
nuclear active Gln3 protein	$[W]$
cytoplasmic inactive Gln3 protein	$[W_{ci}]$
cytoplasmic active Gln3 protein	$[W_{ca}]$
nuclear active Gat1 protein	$[X]$
cytoplasmic inactive Gat1 protein	$[X_{ci}]$
cytoplasmic active Gat1 protein	$[X_{ca}]$
Dal80 protein monomer	$[Y]$
Dal80 active protein homodimer	$[Y_2]$
Gzf3 protein monomer	$[Z]$
Gzf3 active protein homodimer	$[Z_2]$

Table A. Inputs and states used in the GATA network model. Square brackets are used to denote species abundances.

<u>**Protein dynamics**</u>:

$$
\begin{aligned}
[\dot{W}] &= -k_{dW}[W] + k_{imp}^{W}[W_{ca}] - k_{exp}^{W}[W] \\
[\dot{X}] &= -k_{dX}[X] + k_{imp}^{X}[X_{ca}] - k_{exp}^{X}[X] \\
[\dot{W_{ca}}] &= -k_{dW}[W_{ca}] - k_{imp}^{W}[W_{ca}] + k_{exp}^{W}[W] - k_{2w}[W_{ca}] + k_{1w}(t)[W_{ci}] \\
[\dot{X_{ca}}] &= -k_{dX}[X_{ca}] - k_{imp}^{X}[X_{ca}] + k_{exp}^{X}[X] - k_{2x}[X_{ca}] + k_{1x}(t)[X_{ci}] \\
[\dot{W_{ci}}] &= k_{pW}[w] - k_{dWi}[W_{ci}] + k_{2w}[W_{ca}] - k_{1w}(t)[W_{ci}] \\
[\dot{X_{ci}}] &= k_{pX}[x] - k_{dXi}[X_{ci}] + k_{2x}[X_{ca}] - k_{1x}(t)[X_{ci}] \\
[\dot{Y}] &= k_{pY}[y] - k_{dY}[Y] + 2k_{Y_2}^{diss}[Y_2] - 2k_{Y_2}[Y]^2 \\
[\dot{Z}] &= k_{pZ}z - k_{dZ}[Z] + 2k_{Z_2}^{diss}[Z_2] - 2k_{Z_2}[Z]^2 \\
[\dot{Y_2}] &= k_{Y_2}[Y]^2 - k_{Y_2}^{diss}[Y_2] - k_{dY_2}[Y_2] \\
[\dot{Z_2}] &= k_{Z_2}[Z]^2 - k_{Z_2}^{diss}[Z_2] - k_{dZ_2}[Z_2]
\end{aligned}
$$

2.4 Target model

If we denote by $[m]$ the target gene mRNA abundance, we can write the following differential equation for its evolution:

$$
\begin{aligned}
[\dot{m}] = b_m + k_m \frac{k_{mw}[W] + k_{mx}[X] + k_{mw}k_{mx}k_c[W][X]}{1 + k_{mw}[W] + k_{mx}[X] + k_{mw}k_{mx}k_c[W][X] + k_{my}[Y_2] + k_{mz}[Z_2]} \\
- k_{dm}[m],
\end{aligned} \tag{1}
$$

where we used the notation of the previous section for the transcription factor abundances. This model has four unmeasured inputs, generated from the upstream GATA model (and thus not independently controllable) and one measured output, which coincides with its state.

Of the eight new parameters introduced, we had to fix k_m to ensure that the model is structurally identifiable from relative mRNA data, while leaving the rest of the parameters to be estimated from the experimental data. We can easily see why this step is necessary, without using any of the more advanced tools presented in the next Section: considering the TF profiles as input signals, we see that k_m multiplies a function of the inputs that varies between 0 and 1. As the available mRNA data come in the form of relative abundances (i.e. normalized with respect to the first measurement), we can easily see that an infinite number of $(k_m,\ k_{dm},\ b_m)$ combinations is able to reproduce the same output $y = m/m(0)$, unless one of the parameters is fixed. A detailed list of nominal parameter values is provided in Section 2.6.

Each additional target contributes 16 datapoints (8 for each shift), which increase the constraining capability of the augmented dataset. On the other hand, this approach entails a significant model expansion, as one has to consider one extra differential equation with seven free parameters for each target. To maintain the consistency of the model selection process, we kept the model of each target gene exactly the same across the different TF models, as well as its prior parameter distributions. In this way, the first model selection step can be seen as a restricted form of the second, since using just the TF mRNA data makes the downstream effects of the GATA factors "invisible".

2.5 GFP reporter model

Since the original GATA model did not include the GFP reporter dynamics, a small model of the reporter system was appended to the TF network model. The extension comprised three species: GFP mRNA, immature GFP and mature GFP, as described below:

$$\begin{aligned}
\frac{d[mRNA_{GFP}]}{dt} &= k_{rg} f([W],[X],[Y_2],[Z_2]) - k_{drg}[mRNA_{GFP}] \\
\frac{d[GFP_{imm}]}{dt} &= k_{pg}[mRNA_{GFP}] - k_{dg}(t)[GFP_{imm}] - k_{mat}[GFP_{imm}] \\
\frac{d[GFP_{mat}]}{dt} &= k_{mat}[GFP_{imm}] - k_{dg}(t)[GFP_{mat}]
\end{aligned}$$

The regulation function of the GFP gene (f) was assumed to be the same as that of the corresponding GATA promoter driving it, hence dependent on the predicted active form abundances of the GATA factor proteins. Model parameters were either set to arbitrary plausible values, or obtained directly from the available data. More specifically, both production rates $(k_{rg},\ k_{pg})$ were assumed to be equal to one (given that only relative predictions are of interest), while the mRNA half-life $(\log(2)/k_{drg})$ and GFP maturation half-life $(\log(2)/k_{mat})$ were set to, respectively, 20 minutes (median yeast mRNA half-life [41]) and 30 minutes (a plausible value deduced from the previously measured 39 minutes of in vivo EYFP maturation half-life in yeast [18]). Finally, the concentration of GFP was assumed to decrease only due to cell growth, and thus the degradation rates of both mature and immature GFP were set equal to the experimentally measured (time-varying) growth rate of each yeast strain $(k_{dg}(t))$.

It should be noted that all alternative TF models were originally fitted to microarray data, and therefore relative abundances between species were not considered during model selection due to the unreliability of microarrays to quantify inter-species changes. For this reason, we did not expect the TF models to be able to reproduce the ratios of initial GFP measured across strains and conditions. In order to account for relative levels of initial GFP concentrations in the model, an intermediate fine-tuning step was performed in which all candidate TF models were fitted using a likelihood function that, besides the mRNA data, took into account the initial GFP ratios measured with the *DAL80* promoter in the different strains and conditions. This modification allowed us to capture the relative abundance of each GATA

species when predicting the evolution of each GATA-factor levels in the different deletion backgrounds, in the two dynamic shifts.

2.5.1 Interpreting the GFP reporter measurements

A quantitative comparison of experimental validation data and model predictions would not be appropriate for several reasons: besides the fact that the promoter dynamics (e.g. (basal) production rates) of the endogenous GATA promoters and the plasmid-borne promoter fragments are not necessarily identical, the simple linear GFP reporter model is itself simplistic. Moreover, deletions of GATA factors, although not detrimental for cell growth, are bound to alter the behavior of the system. This is not taken into account in our tests, where all parameter values of the GATA network are assumed to be unchanged across all deletion strains.

2.6 GATA model parameters

2.6.1 Transcription factor model parameters

Degradation rates (min^{-1})		Nominal value	Exponent range in prior
k_{dx}	Gat1 mRNA degradation	0.1	$[-1, 1]$
k_{dy}	Dal80 mRNA degradation	0.3	$[-1, 1]$
k_{dz}	Gzf3 mRNA degradation	0.4	$[-1, 1]$
k_{dW}	$Gln3^{nuc}$ and $Gln3^{cyt}_{active}$ degradation	0.033	$[-1, 1]$
k_{dX}	$Gat1^{nuc}$ and $Gat1^{cyt}_{active}$ degradation	0.033	$[-1, 1]$
k_{dWi}	$Gln3^{cyt}_{inactive}$ degradation	0.033	$[-1, 1]$
k_{dXi}	$Gat1^{cyt}_{inactive}$ degradation	0.033	$[-1, 1]$
k_{dY}	Dal80p degradation	0.01	$[-1, 1]$
k_{dY_2}	$Dal80_2p$ degradation	0.077	$[-1, 1]$
k_{dZ}	Gzf3p degradation	0.01	$[-1, 1]$
k_{dZ_2}	$Gzf3_2p$ degradation	0.077	$[-1, 1]$
Production rates ($abundance \cdot min^{-1}$, **fixed**)		Nominal value	Exponent range in prior
k_x	Gat1 transcription rate	0.1	N/A
k_y	Dal80 transcription rate	0.1	N/A
k_z	Gzf3 transcription rate	0.1	N/A
k_{pW}	Gln3p translation rate	100	N/A
k_{pX}	Gat1p translation rate	100	N/A
k_{pY}	Dal80p translation rate	100	N/A
k_{pZ}	Gzf3p translation rate	100	N/A
Translocation | inactivation rates (min^{-1})		Nominal value	Exponent range in prior
k^W_{imp}	Gln3p nuclear import rate	1 (**fixed**)	N/A
k^W_{exp}	Gln3p nuclear export rate	1	$[-2, 2]$
k^X_{imp}	Gat1p nuclear import rate	1 (**fixed**)	N/A
k^X_{exp}	Gat1p nuclear export rate	1	$[-2, 2]$
k_{2w}	Gln3 inactivation rate	1 (**fixed**)	N/A
k_{2x}	Gat1 inactivation rate	1 (**fixed**)	N/A
Dimerization | dissociation rates ($(abundance \cdot min)^{-1}$ | min^{-1})		Nominal value	Exponent range in prior
k_{Y_2}	Dal80p dimerization rate	10	$[-1, 1]$
$k^{diss}_{Y_2}$	$Dal80_2p$ dissociation rate	1	$[-1, 1]$
k_{Z_2}	Gzf3p dimerization rate	10	$[-1, 1]$
$k^{diss}_{Z_2}$	$Gzf3_2p$ dissociation rate	1	$[-1, 1]$

Basal mRNA transcription rates (abundance $\cdot$ min^{-1})		Nominal value	Exponent range in prior
b_x	Gat1 basal rate	0.001	$[-1, 1]$
b_y	Dal80 basal rate	0.001	$[-1, 1]$
b_z	Gzf3 basal rate	0.001	$[-1, 1]$

Regulation function parameters (first letter: target gene, second letter: transcription factor) Nominal value: 0.02 (abundance^{-1}) Exponent range in prior: $[-2, 2]$	
k_{xw}	
k_{xx}	
k_{xy}	
k_{xz}	
k_{yw}	
k_{yx}	
k_{yy}	
k_{yz}	
k_{zw}	
k_{zx}	
k_{zy}	
Interaction constants Nominal value: 10 (abundance^{-2}) Exponent range in prior: $[-2, 2]$	
k_{xwc}	Gln3p-Gat1p interaction on *GAT1*
k_{yxwc}	Gln3-Gat1p interaction on *DAL80*
k_{zxwc}	Gln3-Gat1p interaction on *GZF3*

Input signal parameters	Nominal value Gln+Rap (Exponent range in prior)	Nominal value Pro→Gln (Exponent range in prior)
V_{0w}	0.1 min^{-1} ($[-1, 1]$)	10 min^{-1} ($[-1, 1]$)
V_w	10 min^{-1} ($[-1, 1]$)	0.93 ($[-1.5, 1.5]$)
V_{0x}	0.1 min^{-1} ($[-1, 1]$)	10 min^{-1} ($[-1, 1]$)
V_x	10 min^{-1} ($[-1, 1]$)	0.93 ($[-1.5, 1.5]$)
θ_w	5 min ($[-1, 1]$)	5 min ($[-1, 1]$)
θ_x	5 min ($[-1, 1]$)	5 min ($[-1, 1]$)
n_w	2 ($[-0.75, 0.75]$)	2 ($[-0.75, 0.75]$)
n_x	2 ($[-0.75, 0.75]$)	2 ($[-0.75, 0.75]$)

2.6.1.1 On the choice of exponential parameter ranges and nominal values The choice of nominal values and exponential ranges for the model was based on 1) available information in the lit-

erature regarding some parameter values 2) general intuition about the dynamics of signaling and gene expression systems and 3) intuition about the relative values of the various model states. A more detailed description is provided below. Note that, to ensure the structural identifiability of the full model[1], all protein and mRNA production rates, as well as the deactivation and import rates for the two activators were fixed. Note that the fixing of the production rates establishes arbitrary unit scales for the abundance of different species types in the model. Further details can be found in [29].

mRNA degradation rates: The nominal estimates were based on data reported in [30] and correspond to half-lives of $\sim 2-6$ min ($t_{1/2} = \ln(2)/k$, were k is the degradation rate). Transcription factor mRNA degradation rates are known to be among the highest in yeast, however the dataset of [30] contains mRNAs with even faster degradation (0.2 min). Since the distribution of of yeast mRNA half-lives has a median of 11 min with the 3rd quartile at 33 min [30], we allowed an order of magnitude variation above and below the nominal values to allow for the possibility of quantification errors.

Protein degradation rates: Nominal estimates were based on the dataset of [3], corresponding to half-lives of ~ 20 min. Since protein degradation measurement assays such as the one of [3] are typically fraught with artifacts [43], we allow for two orders of magnitude variation in the prior (the dataset of [3] contains no proteins with half-lives smaller than 2 min, while it is known that transcription factors are typically actively degraded, so a 200 min half-life is a reasonable upper bound).

Translocation rates: Very little is known about precise values for these parameters for the proteins studied here and we are not aware of large-scale studies on protein localization dynamics in yeast. From various targeted studies of other yeast transcription factors such as Msn2 and Hog1 [21, 34, 35], it is known that the processes of import and export can be completed in just a few minutes upon a shift in growth conditions. We therefore selected a high nominal value for the export rate, with relatively large uncertainty (± 2 order of magnitude).

Dimerization/dissociation rates: Again, no information is available for the GATA repressors. In general, it is known that protein dimerization is a fast process that evolves in the timescale of seconds to minutes [27], which suggests a high nominal dissociation rate. Moreover, the available data supports the fact that the Dal80-Dal80 and Gzf3-Gzf3 interactions are quite strong [40] (main text). Since these are also the functional forms of the GATA repressors [40] (main text), we assumed an even higher nominal dimerization rate.

Basal transcription rates: It is not known what fraction of the protein expression in the GATA network is due to constitutive promoter activity. Overall, results from mutant strains, suggest that basal expression is quite low.

Regulation function parameters/interaction constants: The DNA binding properties of the GATA factors have not been studied quantitatively, while various studies have shown that the binding strength of the TFs depends on the target promoter. The regulation function parameters are intimately connected with the TF protein abundances: when a TF is much more abundant than the inverse of its corresponding parameter, the promoter to which it binds operates close to saturation. Given that the maximum nominally attainable TF abundance in our model is in the order of a few thousands (as determined by the transcription, translation and degradation rates for mRNAs and proteins), we chose the nominal regulation function parameters and their range of variation so that the GATA promoters can operate both under saturated and unsaturated conditions. The interaction constant values were chosen solely

[1]Loosely speaking, this amounts to ensureing that the exact same output behavior cannot be reproduced by more than one set of parameter values. Rigorous definitions and results can be found in [9]

based on intuition, due to lack of any relevant data.

Input signal parameters: The functions describing the upstream activation/deactivation rates contain the same basic features: an initial (i.e. prior to perturbation) value, V_0, a final value (determined by V), a steepness factor (n) and the timepoint of 50% saturation (θ). The initial and final values for these rates were selected to yield a $\sim$ 100-fold change in activity after each perturbation, and also lie on both sides of the (fixed) inactivation rate. The timing parameter, θ, was selected based on biological evidence on the speed of upstream signaling processes: it is known that activator localization is already visible after only a few (5-10) minutes after rapamycin treatment or a change in nitrogen source [12], [34] (main text) so the upstream signaling processes most likely evolve at a comparable or faster rate. The lower exponential bound of θ reflects the fact that signaling processes cannot evolve faster than a few tens of seconds, while the upper bound corresponds to a very slow and gradual response.

2.6.2 Target model parameters

Parameter name	Nominal value	Exponent range in prior
b_m	0.01 (abundance $\cdot$ min^{-1})	$[-1, 1]$
k_{dm}	0.045 (min^{-1})	$[-1.5, 1.5]$
k_m	50 (abundance $\cdot$ min^{-1})	N/A (fixed)
k_{mw}	0.002 (abundance^{-1})	$[-2.5, 2.5]$
k_{mx}	0.002 (abundance^{-1})	$[-2.5, 2.5]$
k_{my}	0.002 (abundance^{-1})	$[-2.5, 2.5]$
k_{mz}	0.002 (abundance^{-1})	$[-2.5, 2.5]$
k_c	10 (abundance^{-2})	$[-2.5, 2.5]$

The same arguments presented for the choice of the TF model priors were are also used for picking the target model priors. It should only be noted that the mRNA degradation rate (corresponding to a half-life of about 15 min) was set based on the median half-life values reported in the literature for yeast [30, 41], which range between 10 and 20 min.

2.7 Parametrization of models corresponding to different hypotheses

Models corresponding to different combinations of hypotheses can be obtained from the full model by fixing certain subsets of parameters. The parameters corresponding to each single hypothesis are listed on Table F.

Interaction #	Description	Parameters involved
1	Dal80 self-repression	$k_{yy} = 0$
2	Gat1 self-activation	$k_{xx} = 0$
3	Gzf3 repression of *DAL80*	$k_{yz} = 0$
4	Dal80 repression of *GZF3*	$k_{zy} = 0$
5	Gln3-Gat1 interaction	$k_{xwc},\ k_{yxwc},\ k_{zxwc} = 0$

Table F. Parameters involved in single hypotheses.

2.8 SBML implementation

As stated in the main text, all models were implemented using SBTOOLBOX2 [52] (main text). In order to enable the simulation of the model in other biochemical network simulators, we also converted it into the SBML format. One issue that proved difficult to address was the time varying Gln3 and Gat1 activation rates (and the Gln3 mRNA data interpolation), which are not supported by the SBML format; as a consequence, the SBML representation of our model is not simulation-ready. To simulate the SBML version of our model, the user will first have to import the SBML file into simulation software that allows the definition of time-varying reaction rates. Then, the activation rates of Gln3 and Gat1 (parameters k_{1w} and k_{1x}) will need to be assigned the functional form given in the Methods section of the main text, depending on the shift that is simulated. Moreover, the Gln3 data (given below and shown on Fig. A) will need to be interpolated over time, to be used on the right-hand side of the GLN3 protein production ODE.

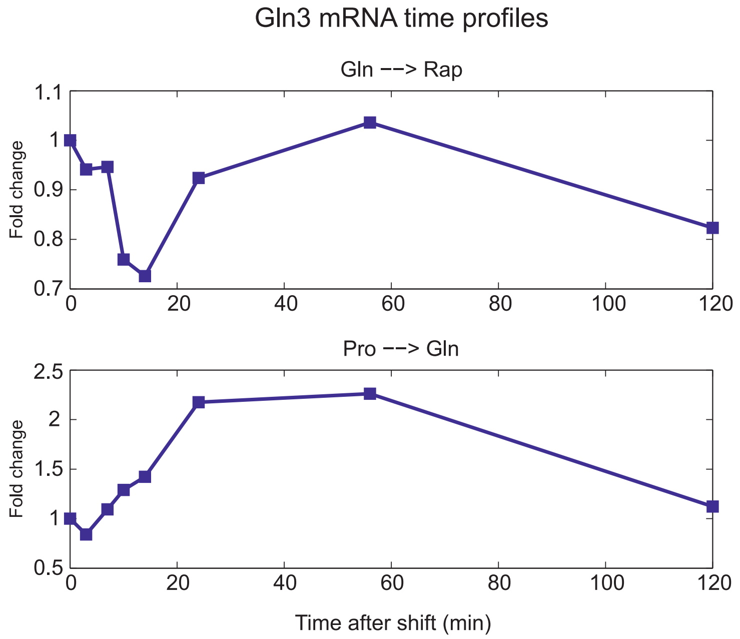

Figure A. Linearly interpolated timecourse of Gln3 gene expression.

2.8.0.1 Gln3 data, Pro→Gln shift: Time (min.): $[0 \quad 3 \quad 7 \quad 10 \quad 14 \quad 24 \quad 56 \quad 80]$
mRNA (fold change): $[1 \quad 0.8397 \quad 1.0933 \quad 1.2902 \quad 1.422 \quad 2.1757 \quad 2.2612 \quad 1.1216]$

2.8.0.2 Gln3 data, Gln+Rap shift: Time (min.): $[0 \quad 3 \quad 7 \quad 10 \quad 14 \quad 24 \quad 56 \quad 80]$
mRNA (fold change): $[1 \quad 0.9411 \quad 0.9462 \quad 0.7593 \quad 0.7259 \quad 0.9239 \quad 1.0359 \quad 0.8232]$

2.9 Details on modeling assumptions

1. Lack of significant external regulators: We assume that there are no external (i.e. non-GATA) regulators of the GATA factors that change their regulatory effect significantly during the shifts considered (with the possible exception of *GLN3* - see Assumption 4 below). To the best of our knowledge today, this assumption is valid [11], [42] (main text). Of course, all GATA factors have many confirmed and potential

regulators according to the Yeastract database (`http://www.yeastract.org`). However, none of these regulators is considered to change significantly during the nitrogen source, or rapamycin shifts. This hypothesis is made implicitly in all studies of the GATA network, but remains to be proven conclusively in the future.

2. TF active forms: Dal80 and Gzf3 are functional as homodimers [40] (main text). On the other hand, both activators are thought to act as monomers. Regarding subcellular localization, Gln3 and Gat1 can be both nuclear (active) and cytoplasmic (both active and inactive), while Dal80 and Gzf3 are only nuclear [1] (main text). It is known that phosphorylation controls the localization of the activators, but no post-translational regulation of repressor activity, other than dimerization, is known.

3. Activator localization mechanism: TOR inhibition upon rapamycin treatment results in a nitrogen starvation phenotype, as it induces fast TF activation and localization in the nucleus. Conversely, the nutrient upshift results in restoration of TOR activity and a quick de-activation and nuclear export of the two activators. These (in)activation signals are assumed to drive the changes in Gln3 and Gat1 localization in response to nitrogen or rapamycin shifts, and therefore act as the primary driving input signals to the system. Capturing them accurately would require a full, validated mathematical model of the TOR network, as well as precise knowledge of how the nitrogen source affects TOR activity. However, the existing state-of-the-art TOR models [37] (main text) still contain unverified hypotheses. Our model was chosen to be simple, but still provide a realistic (in)activation and translocation mechanism, in line with evidence accumulated for similar systems in yeast [22], [18] (main text). According to this mechanism the TFs first get activated (e.g. by phosphorylation, release from an anchor protein or both) and then shuttled across the nuclear membrane, with both processes being reversible and governed by mass-action kinetics. Once quantitative dynamic translocation data become available, this model can be further tested and refined.

4. Gln3 input: We consider Gln3 mRNA (Fig. A) as a secondary input to our model, and further assume that mRNA changes are reflected in the abundance of Gln3 protein, which consequently has to change over time. This assumption is based on the observation that Gln3 mRNA displays moderate fluctuations during both shifts considered. These small but significant Gln3 mRNA changes in all timecourse experiments imply that *GLN3* is weakly controlled by proteins other than the GATA factors (Gcn4 could be implicated in this regulation [42] (main text)).

5. Steady-state conditions: The system is assumed to be at steady-state prior to each shift, an assumption that is approximately correct given that the yeast cells were growing exponentially before the perturbations.

6. No Gat1-Gzf3 complexes: A recent study presented evidence for negative regulation of Gat1 by Gzf3 through the formation of a Gat1-Gzf3 heterodimer [31] (main text). Since it is still very unclear how this interaction occurs mechanistically, what is its strength and its physiological significance, we decided not to include it in the model. Previous work has presented hints of interactions between the zinc-finger regions of Gln3 and Dal80, as well as Gln3 and Gzf3, both much weaker than the interactions in the Dal80 and Gzf3 homodimers respectively, and has cautiously avoided giving biological significance to the finding [40] (main text). It is thus very likely that the Gat1-Gzf3 complexes are also a result of a "residual" interaction that arises due to the large degree of homology shared by all GATA factors.

7. No Dal80-Gzf3 dimers/active Gzf3 monomers: We elected to ignore the Dal80-Gzf3 heterodimer from our model, for two reasons: no DNA binding site has yet been identified for Dal80-Gzf3, while the strength of this interaction is most likely much weaker than the Dal80-Dal80 and Gzf3-Gzf3 interactions [40] (main text). We further assumed that Gzf3 monomers play no role in gene regulation: the experimental data in favor of the opposite is very weak [32] (main text), while it was recently shown that deletion of the Gzf3 dimerization domain abolishes its repressing ability [31] (main text), a very strong indication that repression is exerted by the homodimer.

8. Non-competitive TF binding: Transcription factor binding is assumed to be non-competitive, i.e. several TFs are assumed to be able to bind to the same promoter simultaneously. This assumption is

reflected in the form of the fractional occupancy functions for each GATA factors promoter. The plethora of GATA binding sites both on GATA-factor and target promoters indicates that this simplification is realistic. Moreover, testing all possible binding configurations for each promoter would be infeasible computationally.

9. Model changes between shifts: The only parts of the model that are assumed to vary from one shift to the other are the parametrization of the input signal and the Gln3 mRNA input. All other parameters are assumed not to vary between shifts, as no concrete evidence is currently available regarding which parameters should vary and to what extent.

Among our assumptions, #5 is reasonable and necessary for preventing nonsensical model predictions, and #9 is made to prevent unnecessary overfitting given the available amount of data and the lack of biological evidence for the alternative. Assumptions #6, 7 and 2 are based on currently available evidence that allows us to simplify our model, while the lack of specific mechanistic details (such as the unknown binding site of Dal80-Gzf3 or the fact that Gzf3 monomers can still weakly bind to DNA but cannot repress expression) make their mathematical encoding very difficult. Similarly, assumption #8 is the most plausible choice based on the lack of concrete biological evidence and computational limitations. Finally, assumptions # 1, 3, and 4 are required for defining the model "boundaries" and its interactions with the rest of the cell.

3 Bayesian model selection and the SMC sampler for the GATA network

3.1 Bayesian model selection

Consider a set of competing biological hypotheses $\{\mathcal{H}_k\}_{k=1}^{K}$, each represented by a mathematical model $\mathcal{M}_k$, to be compared given experimental data D. For each model $\mathcal{M}_k$, dataset D is assumed to have a density $P(D|\mathcal{M}_k, \boldsymbol{\theta}_k)$, where $\boldsymbol{\theta}_k$ is the parameter vector of model $\mathcal{M}_k$. $P(D|\mathcal{M}_k, \boldsymbol{\theta}_k)$ is known as the *likelihood function* [48] (main text). It encodes the fact that the dataset D is corrupted by measurement noise and its functional form depends on our assumptions about the measurement error distribution. Our prior knowledge about parameter values and plausibility of the models is encoded by the prior distributions $P(\boldsymbol{\theta}_k|\mathcal{M}_k)$ and $P(\mathcal{M}_k)$ respectively [48] (main text) (details on the choice of measurement noise model and priors for our model are presented in Subsection 3.3).

The first key object in Bayesian model selection is the marginal density of D under $\mathcal{M}_k$,

$$P(D|\mathcal{M}_k) = \int P(D|\mathcal{M}_k, \boldsymbol{\theta}_k) P(\boldsymbol{\theta}_k|\mathcal{M}_k)\, d\boldsymbol{\theta}_k, \tag{2}$$

also called the *evidence* for $\mathcal{M}_k$. The second is the *Bayes factor* [25] B_{ij} of $\mathcal{M}_i$ to $\mathcal{M}_j$, given by

$$B_{ij} = \frac{P(D|\mathcal{M}_i)}{P(D|\mathcal{M}_j)}, \tag{3}$$

which can be interpreted as the "weight of evidence" provided by the data in favor of model i as opposed to model j. For our purposes, we will use the fact that a Bayes factor greater than 10 is commonly interpreted in practice as strong evidence in favor of model i. A scale that matched Bayes factor values to evidence support categories can be found in [25].

The Bayes factor is used to update our initial beliefs in models i and j and obtain the posteriors ratio

$$\frac{P(\mathcal{M}_i|D)}{P(\mathcal{M}_j|D)} = \frac{P(D|\mathcal{M}_i)}{P(D|\mathcal{M}_j)} \frac{P(\mathcal{M}_i)}{P(\mathcal{M}_j)} = B_{ij} \frac{P(\mathcal{M}_i)}{P(\mathcal{M}_j)}. \tag{4}$$

The model posterior probability $P(\mathcal{M}_k|D)$ is the fundamental quantity in Bayesian model selection. Having the Bayes factors, we can compute it as

$$P(\mathcal{M}_k|D) = \left[\sum_{j=1}^{K} \frac{P(\mathcal{M}_j)}{P(\mathcal{M}_k)} B_{jk}\right]^{-1}. \tag{5}$$

$P(\mathcal{M}_k|D)$ can be interpreted as a measure of the "plausibility" of model k after all information provided by D has been considered. Thus, model selection is accomplished by choosing the most probable model (or models, in case the data is not discriminative enough) [8].

This approach to model selection naturally strikes a balance between data misfit and model complexity. The key to this balance comes from (2): generally speaking, a simple model $\mathcal{M}_s$ has a limited predictive ability, which means that its evidence $P(D|\mathcal{M}_s)$ is concentrated in a small region of the space of all possible datasets D. On the contrary, a more complex model $\mathcal{M}_c$ is able to generate a larger variety of datasets, which however implies that its evidence is spread over a larger region of the data space. If the data we observe fall within the high-density region of $P(D|\mathcal{M}_s)$, the simpler model will end up as the most probable model assuming that both models have equal priors (Ch.28 of [48] (main text)).

3.1.1 Discriminating among multiple hypotheses using a single multimodal prior

Assume that that there are K hypotheses to be tested, and that the absence or presence of hypothesized interaction k is equivalent to a certain model parameter, say θ_k, being zero or nonzero. In this case, one can consider the "full" model (i.e. the model with all interactions present) and assign to each of those K parameters a "spike and slab" prior for, as in [31]:

$$\pi(\theta_k) = w_k \delta(\theta_k) + (1 - w_k) f_k(\theta_k).$$

This prior is a mixture of a point mass at zero and an absolutely continuous distribution f_k, whose support does not include zero. The weight w_k indicates our prior belief that θ_k is zero (eqv. the corresponding hypothesis is not present). If the w_k's are fixed for all θ_k's (e.g. to 0.5) and the priors for different parameters are independent, then the total posterior $P(\{\theta_k\}_{(k=1,,K)}|D)$ will be a mixture distribution with 2^K components, $\{p_m\}_{(m=1,,2^K)}$, each corresponding to one of the possible products of point mass and f_k priors. With respect to the model selection problem, the precise form of these posterior components does not matter. What matters instead, is their relative contributions to the overall posterior, because these indicate which combination of parameters is more likely to be non-zero. In turn, for each posterior mixture component, p_m, this contribution is given by the integral of the likelihood with respect to π_m, and these are precisely the numbers we report in this work.

A further generalization of the above problem would be a fully Bayesian approach, where the prior weights w_k are not assumed constant, but are assigned a hyperprior distribution (as described, for example, in [16]), according to which each weight can only be 0 or 1 with certain probability. In this case, the goal is to infer the posterior weight distribution given the data; weights with a high posterior probability of being 1 correspond to parameters that should be set to zero. Here as well, it is easy to see that the computation of the weight posterior necessarily involves the computation of the marginal likelihoods corresponding to each combination of "spikes" and "slabs". Under the uninformative, uniform prior that assigns probability 2^{-K} to each weight combination, the maximum of the weight posterior coincides with the top-ranking model, as identified by our approach (different, more informative weight hyperpriors would of course result in different model ranking).

3.2 Algorithm

As stated in the main text, simple approaches for approximating the evidence, such as plain Monte Carlo integration, Importance Sampling-based estimators or even Markov Chain Monte Carlo (MCMC)

samplers quickly become inefficient as the number of parameters grows: given the fact that the fraction of measured to the total number of states in large models is usually very small, likelihood distributions may become highly correlated and/or multimodal, due to model nonlinearities and the inevitable practical identifiability problems that follow. Moreover, the ratio of the feasible parameter volume (i.e. the volume of the parametric sets leading to good model fits) to the total available volume determined by the (usually very diffuse) priors typically decreases substantially with increasing model complexity.

The recently introduced Sequential Monte Carlo (SMC) (or Population Monte Carlo) [7, 23, 24], [23] (main text) methods are able to overcome the difficulties of sampling from high-dimensional, correlated and multimodal distributions. These methods are intimately connected with sequential importance (re)sampling and less related to Markov Chain Monte Carlo (although MCMC kernels are often among their ingredients), and are able to handle complex distributions by building a sequence of increasingly better proposals. Each distribution is represented by a large collection of random samples ("particles") that get propagated through the intermediate steps using ideas from importance sampling. Contrary to MCMC methods, the population at each step can depend in an arbitrary way on past samples, while the approximation to the target at each iteration remains unbiased and does not require throwing away burn-in samples [37]. On the other hand, bias can be accidentally introduced in these methods if the population diversity (quantified for example by its effective sample size) gets reduced significantly, and it is necessary to use large population sizes (at an increased computational cost) to properly sample high-dimensional distributions. Fortunately, recent results indicate that SMC algorithms are stable with an $O(Nd^2)$ computational cost, where N is the number of particles and d the dimension of the target distribution, when $O(d)$ intermediate distributions are considered [4]. For comparison, the computational cost to maintain stability of importance sampling increases exponentially with d.

The SMC sampler implemented in this work is able to circumvent the problem of sampling from complex, high-dimensional posteriors by defining a sequence of bridging distributions f_β according to a "cooling schedule":

$$f_{\beta_i}(\boldsymbol{\theta}) \propto P(D|\mathcal{M}, \boldsymbol{\theta})^{\beta_i} P(\boldsymbol{\theta}|\mathcal{M}), \tag{6}$$

for $0 = \beta_0 < \beta_1 < \cdots < \beta_N = 1$.

The basic idea of the algorithm is to propagate a population of particles sampled from the prior through this sequence of intermediate distributions that gradually morph into the target posterior. At each step, distribution $f_{\beta_n}(\boldsymbol{\theta})$ serves as an importance distribution for $f_{\beta_{n+1}}(\boldsymbol{\theta})$. Starting from the sampled points of $f_{\beta_n}(\boldsymbol{\theta})$, the new samples from $f_{\beta_{n+1}}(\boldsymbol{\theta})$ are drawn using a Markov chain transition kernel $K_n(\cdot|\boldsymbol{\theta}^j_{(n-1)})$ that leaves $f_{\beta_n}(\boldsymbol{\theta})$ invariant. The great flexibility of the method stems from the fact that this is the only requirement imposed on the kernels, which can be selected to be arbitrarily complex. One of the simplest choices is to let $K_n(\cdot|\boldsymbol{\theta}^j_{(n-1)})$ consist of a few (10-20) Metropolis-Hastings updates of $\boldsymbol{\theta}^j_{(n-1)}$ with $f_{\beta_n}(\boldsymbol{\theta})$ as the target density. It is not necessary that the K_n's mix fast, although this is desirable for reducing the weight variance [33]. In any case, the importance weights may still end up with large variance after some number of annealing steps, resulting in a small effective sample size (ESS). This problem can be avoided by monitoring the ESS at each step n and resampling the particles from the current approximation of f_{β_n} if $ESS < 0.5M$ [23] (main text).

The pseudocode for the SMC sampler is given in Algorithm 1. This is a modified version of Algorithm 3.1.1 given in [23] (main text), to take into account the specific form of Markov kernels used here, which are a special case of the generic transition kernels assumed in [23] (main text). Below we proceed to describe in more details our choices for the individual algorithm components.

3.3 Model-related settings

3.3.1 Prior selection and parametrization

Only a few parameters in our model have been measured and reported in the literature, often for different growth conditions and yeast strains. One has thus to cope with the problem of large parameter uncertainty, which necessitates the use of wide prior distributions spanning several orders of magnitude. To model this uncertainty we use the log-uniform prior, which assigns equal likelihood to all orders of magnitude in a given interval and is scale-invariant. The same prior distributions were used for all common parameters across the different model structures.

To arrive at the log-uniform prior for each model parameter θ'_i, we first determined a nominal value $\theta'_{i,nom}$. We subsequently defined the log-ratios $\theta_i = \log_{10}\left(\frac{\theta'_i}{\theta'_{i,nom}}\right)$. The uniform priors for the θ_i's, centered at $\theta_i = 0$ and supported on $[-a_i, a_i]$, correspond then to log-uniform distributions in the original (linear) parameter space. In paragraph 2.6.1.1 we provide further information on the specifications of these priors, namely the nominal values $\theta_{i,nom}$ and exponential ranges $[-a_i, a_i]$. From this point on, to simplify the presentation the θ_i's will be called the system parameters.

3.3.2 Noise model

With only three biological replicates for three timepoints in each experimental timecourse (as described in the main text), the reliable estimation of a noise model for the different biological replicates is not feasible. On the other hand, there is also a noise component stemming from the various sources of errors in the microarray processing, which is quite hard to model accurately. However, a general consensus seems to be that this noise is of a broadly multiplicative nature, i.e. its variance grows as mRNA abundance increases. In an attempt to combine both noise sources in a simple and tractable noise model, we thus made the assumption of independent, identically distributed additive Gaussian noise with time-varying variance, which makes each measurement normally distributed with a CV derived from the average CV of the fold changes in all mRNAs measured with the microarrays in each shift (3 timepoints x 3 replicates per gene).

More concretely, we assume each measurement

$$y(t),\ t \in T,$$

where $T = \{3, 7, 10, 14, 24, 56, 120\}$ is the vector of measurement times, to be of the form

$$y(t) = x + w,$$

where x is the actual mRNA fold change and $w \sim (0, y(t)^2\sigma^2)$. This implies that $y(t) \sim \mathcal{N}(x(t,\theta), y(t)^2\sigma^2)$, and $CV_y \simeq \sigma$ (if we assume x lies close to y). We used $\sigma_1^2 = 0.025$ for the Pro→Gln dataset and $\sigma_2^2 = 0.01$ for the Gln+Rap data.

Based on this noise model, the appropriate likelihood function $P(D|\theta)$ was defined. Its negative logarithm has the form of weighted squares sum:

$$-\log(P(D|\theta)) = \frac{1}{2}\sum_{t\in T}\sum_{i=1}^{3}\left(\frac{x_i(t,\theta) - y_i(t)}{\sigma y_i(t)}\right)^2 + c,$$

where c is the logarithm of the normalizing constant.

Algorithm 1

Require: A sequence of distributions $\{\pi_n\}_{n=1}^N$ defined according to (7)

Ensure: A weighted sample approximation $\{W_N^{(m)}, \theta_N^{(m)}\}_{m=1}^M$ of $\pi_N(\theta)$ and an estimate $\widehat{Z}_N$ of $Z_N = \int \pi_N(\theta)d\theta$

Initialize: Set $n = 1$ and $r = 1$ (vector of steps where resampling occurred)

for $m = 1, \ldots, M$ **do**

Draw $\theta_1^{(m)} \sim \pi_1(\theta)$

Evaluate the unnormalized incremental weights $\tilde{w}_2(\theta_{1:2}^{(m)}) = \dfrac{\pi_2(\theta_1^{(m)})}{\pi_1(\theta_1^{(m)})}$

end for

Normalize $\{\tilde{w}_2^{(m)}\}_{m=1}^M$ to obtain $\{W_2^{(m)}\}_{m=1}^M$

for $n = 2, \ldots, N$ **do**

If $ESS_n < 0.5M$, resample the population $\{\theta_{n-1}^{(m)}\}_{m=1}^M$ according to weights $\{W_n^{(m)})\}$, set $W_n^{(m)} = 1/M$ and $r = [r\ n]$

for $m = 1, \ldots, M$ **do**

Draw $\theta_n^{(m)} \sim K_n(\theta_{n-1}^{(m)})$

Evaluate $\tilde{w}_n(\theta_{n-1:n}^{(m)}) = \dfrac{\pi_n(\theta_{n-1}^{(m)})}{\pi_{n-1}(\theta_{n-1}^{(m)})}$

end for

Calculate particle weights $\left\{ W_n^{(m)} = \dfrac{W_{n-1}^{(m)}\tilde{w}_n(\theta_{n-1:n}^{(m)})}{\sum_{m=1}^M W_{n-1}^{(m)}\tilde{w}_n(\theta_{n-1:n}^{(m)})} \right\}_{m=1}^M$

end for

Set $r = [r\ \ N]$ and calculate $\widehat{Z}_N = \displaystyle\prod_{j=1}^{\text{length}(r)+1} \frac{1}{M} \sum_{m=1}^M \left(\prod_{n=r_j+1}^{r_{j+1}} \tilde{w}_n(\theta_{n-1:n}^{(m)}) \right)$ {Note: $ESS_n = 1/(\sum_{m=1}^M (W_n^{(i)})^2)$}

3.4 Sampler settings

3.4.1 Cooling schedule

As described in Section 3.2, we start by defining a sequence of intermediate distributions that connect the diffuse (and known) parameter prior, $P(\boldsymbol{\theta}|\mathcal{M})$, to the concentrated posterior $P(\boldsymbol{\theta}|D,\mathcal{M})$, for which only know that is proportional to $P(D|\mathcal{M},\boldsymbol{\theta}) \cdot P(\boldsymbol{\theta}|\mathcal{M})$. We thus define the intermediate distributions as

$$\pi_n(\boldsymbol{\theta}) \propto P(D|\mathcal{M},\boldsymbol{\theta})^{\beta_n} P(\boldsymbol{\theta}|\mathcal{M}), \tag{7}$$

where $0 \leq \beta_1 \leq \beta_2 \leq \cdots \leq \beta_N = 1$ is a predetermined *cooling schedule*, i.e. an increasing set of parameters that determines how fast we move from the initial to the final distribution, and can be thought of as "inverse temperature". The term cooling is used to reflect the fact that the sequence of distributions evolves from the most "disordered" one (the prior) to the most "ordered" (the posterior). While theoretically an optimal [2] temperature schedule can be derived for each problem instance, its practical evaluation is not possible except for very simple cases. The results obtained in [5] suggest however that geometric temperature schedule should be close to the optimal. In the same study it was also noted that as the uncertainty encoded by the priors increases, the more the intermediate temperatures have to be clustered towards 0, since the low temperature region is where the rate of change of the intermediate densities is greatest. This prompted us to use a temperature schedule of the form

$$\beta_n = (n/N)^4,$$

where n ranges from 0 to n and a large number of intermediate distributions is considered ($N = 70$). We arrived at this specific form by repeatedly testing different exponents and step numbers, with the objective of reducing the frequency of resamplings, particularly at high temperatures (low β values), where the parameter space has to be sufficiently explored.

3.4.2 Transition kernels

The SMC algorithm requires the use of a sequence of transition kernels $\{K_n\}_{n=1}^N$, that are used to propose new particles at each cooling step n by perturbing the particles $\{X_{n-1}^{(i)}\}$ obtained at step $n-1$. We set each K_n as an MCMC kernel with invariant distribution π_n), defined by 15 iterations of an independent Metropolis-Hastings (IMH) sampler with proposal distribution q_n obtained by fitting a Gaussian mixture distribution to the particle population $\{X_{n-1}^{(i)}\}$ from step $n-1$.

The use of the more straightforward Gaussian random walk proposals in place of the IMH, while giving satisfactory results in smaller parameter dimensions, was not adequate for sampling in more than 15-20 dimensions. Its main drawback derives from the great differences between the extremes of the distribution sequence: for small β;s, large moves need to be proposed, in order to fully explore the parameter space and locate regions of potential interest. As the β increases, however, and the particles concentrate in high-density regions of the posterior, the proposed moves must be much smaller to ensure non-zero acceptance probabilities, otherwise the particles will be left essentially unperturbed and the SMC sampler will degenerate to the extremely inefficient Importance Sampler. One could try to alleviate this problem by tuning the covariance matrix of the proposal density in a way that depends on the cooling schedule, or use a mixture of kernels with a large range of bandwidths, yet this is neither an easily implementable or computationally efficient approach. Finally, use of Adaptive MCMC schemes [1, 2, 20] is not advisable in this case, since they cannot be used for such a small number of iterations (they converge to the target distribution after an adaptation phase).

On the other hand, Gaussian mixtures (GM's) are a versatile class of semi-parametric models that are very commonly used for density estimation [28]. Although the optimal number of components for a

[2] in the sense of minimizing the Monte Carlo variance of the evidence estimates

given distribution can be obtained using model selection methods, we chose to keep it constant in this work, after extensive offline experimentation with distributions generated from the AIS-SMC sampler, which showed that the optimal number of components very rarely exceeded six or seven. We used the Expectation-Maximization (EM) algorithm [32] to obtain the maximum-likelihood estimates for the mixture parameters, and considered full covariance matrices for each component.

The use of GM's as importance densities has recently been introduced in the framework Adaptive and Multiple Adaptive Importance Sampling with very promising results [6, 11, 42]. It has also been shown [11] that the maximum-likelihood parameter estimates for these mixtures are also the ones that minimize the Kullback-Leibler divergence from the target distribution (in our case π_n) to its mixture approximation, provided one considers the particle weights in the EM algorithm. In our GM models the particle population is considered unweighted to simplify a bit the computations, but our fits do not deviate much from the optimal case, since the variance of the particle weights is not allowed to increase much [3]. This is accomplished by monitoring the Effective Sample Size (ESS) of the population, which is not allowed to decrease below $M/2$, where M is the number of particles.

3.4.3 Resampling strategy

As mentioned above, the quality of our particle population at each cooling step is estimated using the Effective Sample Size (ESS) criterion, that takes values between 1 and M (where M is the population size), and represents the number of particles that effectively contribute to the current Monte Carlo approximation of the density at that step. The ESS is calculated from the particle weights at each step [23] (main text) and when it drops below $M/2$, the particles are resampled according to the residual resampling scheme [26], that offers reduced variance in comparison to the standard multinomial sampling method. The resampled particles are then assigned equal weights before getting propagated to the next target distribution.

3.4.4 Population size

The evidence estimates provided by the SMC algorithm have been shown to converge almost surely to the true value as the number of particles tends to infinity [23] (main text). In the same work, an analytical expression of the variance of the SMC sampler is provided, which however is impossible to evaluate practically. We therefore had to resort to numerical experiments to determine the necessary number of particles that achieves an acceptable[4] variance of the estimates. Therefore, the same practice was used in all of our tests: starting from a small population size, the SMC sampler was run for increasing numbers of particles, until all estimates obtained for any evidence were constrained sufficiently tightly. For each run of the first model selection round, a population of 15000 particles was used; runs of the second round used 10^5 particles, due to the increased dimensionality of the problem.

4 Sampling parameters for modular systems

The evidence decomposition described in Materials and Methods can be generalized when multiple subsystems are jointly affected by the first one but do not interact with each other. Consider for example the following form of f:

$$f(\mathbf{x}_1, \mathbf{x}_2, \mathbf{x}_3, \theta_1, \theta_2\theta_3) = \begin{bmatrix} f_1(\mathbf{x}_1, \theta_1) \\ f_2(\mathbf{x}_1, \mathbf{x}_2, \theta_2) \\ f_3(\mathbf{x}_1, \mathbf{x}_3, \theta_3) \end{bmatrix},$$

[3]We have also verified experimentally that this minor change does not have any impact at all on the obtained parameter posteriors and evidence estimates

[4]In the sense of allowing us to draw safe conclusions about model ranking

where $\mathbf{x}_i$ and θ_i have to be interpreted as in the simpler case above. If we assume that there are available measurements of both $\mathbf{x}_2$ and $\mathbf{x}_3$ states (denoted by D_2 and D_3 respectively), we can write

$$P(D_1, D_2, D_3|\theta_1, \theta_2, \theta_3) = P(D_1|\theta_1)P(D_2|\theta_1, \theta_2)P(D_3|\theta_1, \theta_2). \tag{8}$$

This setup is summarized in Figure B.

In this case we exploit the fact that the marginal posteriors of θ_2 and θ_3 are *conditionally independent* given θ_1. More analytically,

$$P(D_1, D_2, D_3) = \tag{9}$$

$$= \iiint P(D_1|\theta_1)P(D_2|\theta_1, \theta_2)P(D_3|\theta_1, \theta_3)\pi(\theta_1)\pi(\theta_2)\pi(\theta_3)d\theta_1 d\theta_2 d\theta_3 \tag{10}$$

$$= P(D_1)\int P(D_2|\theta_1, \theta_2)P(D_3|\theta_1, \theta_3)P(\theta_1|D_1)\pi(\theta_2)\pi(\theta_3)d\theta_2 d\theta_3. \tag{11}$$

Arriving at the same form of integral as previously, we observe that the integrand factorizes into a product of two terms, whose common element is θ_1. This enables us to use a blocking approach for its numerical evaluation, in which we first sample the θ_1 vector from its posterior and, given this, draw independent θ_2 and θ_3 samples to calculate the product term.

4.1 Parameter sampling for decomposed systems using SMC

To demonstrate how the reformulation of the evidence translates into the SMC implementation, we will take up the example of an upstream subsystem (denoted by M_1) and two downstream modules (denoted by M_2 and M_3) with the same parameter and dataset definitions as above (note that model notation bears no relation to GATA model naming). The module interconnections are shown on Figure B.

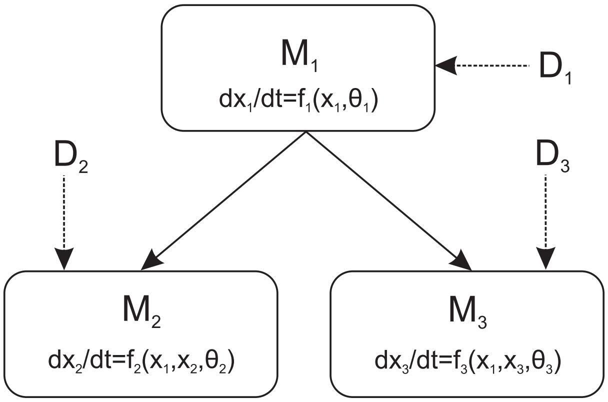

Figure B. A system consisting of an upstream and two downstream subsystems. In the case of the GATA network, the upper module comprises the four GATA factors and their interactions, while the lower ones correspond to GATA-factor target genes. This structure of interactions is exploited in the next Section for facilitating parameter sampling in the high-dimensional parameter space of the composite system. Note that our approach can be easily generalized to the case of multiple downstream modules (i.e. target genes).

Assuming the estimation of $P(D_1)$ and the particle approximation of $P(\theta_1|D_1)$ have been already obtained, one has to then estimate an integral of the form

$$P(D_1, D_2, D_3) = P(D_1)\int P(D_2|\theta_1, \theta_2)P(D_3|\theta_1, \theta_3)P(\theta_1|D_1)\pi(\theta_2)\pi(\theta_3)d\theta_2 d\theta_3. \tag{12}$$

The parameter "prior" in this case consists of the product of the upstream module posterior and the downstream module priors, and can be approximated arbitrarily well if we replace the posterior distribution by a good GM approximation. From this approximate prior we can then draw the initial samples required by the SMC algorithm. These samples are subsequently perturbed and propagated through the sequence of intermediate distributions, following the basic scheme of Algorithm 1, with a crucial change: the conditional independence of parameter blocks allows us to perform randomized block updates of the total parameter vector, $\theta = [\theta_1\ \theta_2\ \theta_3]$, during the Metropolis Hastings iterations.

This block updating can be implemented efficiently by constructing three GM approximations (additional to the initial θ_1 posterior approximation) at each annealing step n: the first approximates

$$\pi_{n-1}(\theta_1) = P(D_2|\theta_1)^{\beta_{n-1}} P(\theta_1|D_1)$$

(the annealed target distribution of the previous step, marginalized over θ_2 and θ_3), while the second and third represent

$$\pi_{n-1}(\theta_2) = P(D_2|\theta_2)^{\beta_{n-1}} \pi(\theta_2)$$

and

$$\pi_{n-1}(\theta_3) = P(D_3|\theta_3)^{\beta_{n-1}} \pi(\theta_3)$$

(the corresponding annealed marginal target distributions at step $n-1$).

All GM distributions were fitted to the particle approximation $\{X_{n-1}^{(i)}\}$ of the annealed target distribution at step $n-1$. Notice that it would have been more precise to employ GM approximations to $\pi_{n-1}(\theta_1, \theta_2)$ and $\pi_{n-1}(\theta_1, \theta_3)$ (the conditional distributions of downstream parameters given θ_1), however the cost of calculating the conditional variances at each proposal step made the computational cost of the method forbiddingly large. We thus resorted to a cruder -but at least two orders of magnitude faster- approximation of the conditional distributions by the marginals. This approximation proved quite good in practice, since the correlations between the upstream and downstream parameters were not observed to be too strong. Even if this was not the case, we believe this approach would be much more efficient than true conditional approximation by a Gaussian Mixture.

4.2 Modular sampling for the GATA system

In the case of the GATA network, the module decomposition we consider is displayed on Fig. C below, with the "upstream" system being the transcription factor network, and the "downstream" systems being the six GATA targets.

To write the evidence decomposition for this particular example, we first define the following notation (cf. with Subsection 3.1):

- D_{TF}: TF mRNA dataset for both shifts considered together
- $\{D_{target,i}\}_{i=1}^{6}$: target mRNA datasets for both shifts
- $\boldsymbol{\theta}_k^{TF}$: parameter vector for the k-th TF model structure
- $\{\theta_{target,i}\}_{i=1}^{6}$: parameter vectors for the target models
- $\widehat{\mathcal{M}}_k$: the k-th TF model structure, augmented with the six target models

With these definitions, we can write the complete data evidence of model $\widehat{\mathcal{M}}_k$:

$$\begin{aligned} & P(D_{TF}, \{D_{target,i}\}_{i=1}^{6}|\widehat{\mathcal{M}}_k) = \\ & = P(D_{TF}|\mathcal{M}_k) \int \left(\prod_{i=1}^{6} P(D_{target,i}|\boldsymbol{\theta}_k^{TF}, \theta_{target,i}) \pi(\theta_{target,i}) \right) P(\boldsymbol{\theta}_k^{TF}|D_{TF}) d\boldsymbol{\theta}_k^{TF} \prod_{i=1}^{6} d\theta_{target,i} \\ & = P(D_{TF}|\mathcal{M}_k) F_k(D_{TF}, \{D_{target,i}\}_{i=1}^{6}) \end{aligned}$$

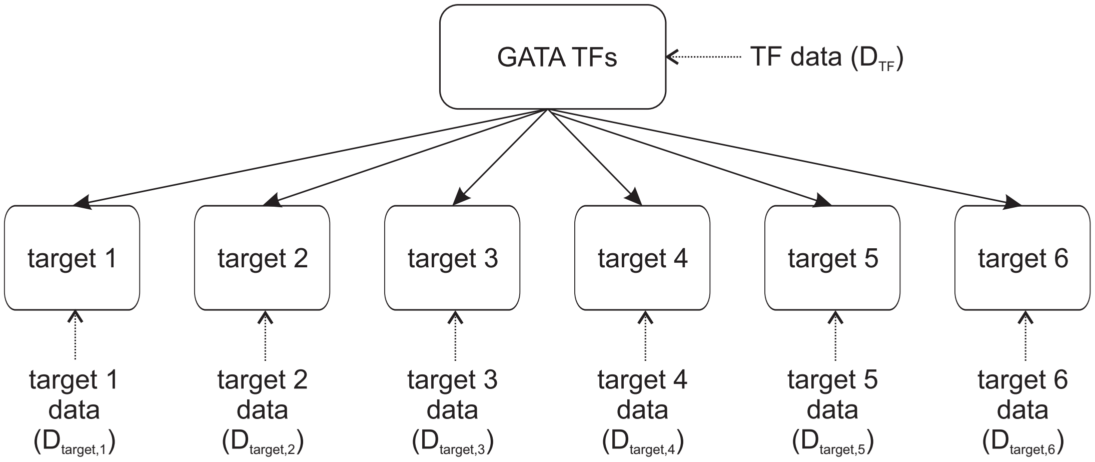

Figure C. The decomposition in the case of the GATA network: the upper module comprises the four GATA factors and their interactions, while the lower ones correspond to GATA-factor target genes. This structure of interactions is exploited in the next Section for facilitating parameter sampling in the high-dimensional parameter space of the composite system.

following the approach (and notation) of the corresponding Materials and Methods section. We thus see that the evidence of $\widehat{\mathcal{M}}_k$ is equal to the evidence of $\mathcal{M}_k$ (already available from the first model selection round), multiplied by a factor F_k that can be estimated with a subsequent SMC run, as shown in the previous Subsection.

We should remark that the above evidence decomposition for model $\widehat{\mathcal{M}}_k$ does not involve any approximation, only a sequential computation that requires two SMC runs, with the result of the first run feeding into the second.

5 Parameter inference and model selection with SMC

5.1 Example: parameter inference for the full model

Using our SMC sampler we obtained parameter posteriors for each of the 32 models considered. Below we present in more detail the results for the full model ($\mathcal{M}_0$), but we should remark that a detailed comparison of posteriors of different models indicates that several parameter marginals remain unchanged, while others display significant changes. This phenomenon can provide useful insight into how different model structures "adapt" to the available data, and how the different parts of the model contribute to its overall behavior, however it will not be further examined here.

Proper visualization of high-dimensional distributions is quite hard, so we have to base our presentation on several mutually complementary views of the results. Figure D displays the marginal posteriors for the free parameters of $\mathcal{M}_0$. (**Note: whenever we refer to the "system parameters", the logarithmic fold-change parametrization will be implied - cf. with Section 3.3**). We observe that some of the distributions are very wide, while other model parameters have very tight posteriors, indicating high likelihood sensitivity to these coordinates.

To get a better idea about parameter correlations in the posterior (which could be responsible for several of the wide marginals as well), we calculated the correlation matrix for the posterior population, which is displayed on Figure E. We can observe several strong correlations among parameters - a direct consequence of the limited amount of available experimental data, which are insufficient to properly

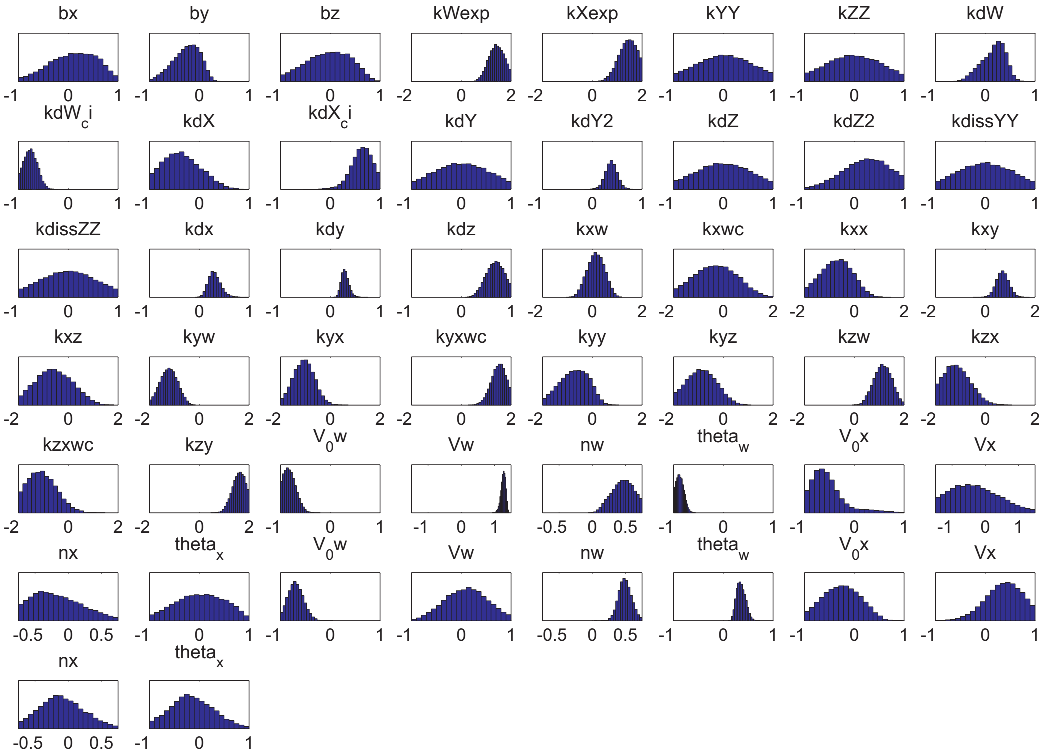

Figure D. Marginal posterior distributions for $\mathcal{M}_0$ parameters. Parameters related to translocation signal are repeated twice, since we used data from two shifts. The first set of signal parameters corresponds to Pro→Gln shift, and the second to Gln+Rap.

constrain all parameters independently of each other. Several wide posteriors can be thus explained as the result of strong pairwise correlations, such as the distributions of k_{xx}, k_{xwc}, k_{xz} etc. We can also observe the behavior of a few truly insensitive parameters, such as k_{YY} and k_{ZZ}, which lack any significant correlation with others and yet display very wide posteriors (this result confirms our findings from an independent global sensitivity analysis of these parameters [38]).

Shifting our view from individual parameters to parameter sets, we can get a good idea about "stiff" and "sloppy" parameter directions (following the terminology introduced in [19]) by examining the eigenvalues and eigenvectors of the population covariance matrix (Principal Component Analysis). The number of large eigenvalues of the covariance matrix, shown on Figure F indicates that several sloppy parameter combinations probably exist. On the other hand, the combinations of parameters to which the model appears to be the most sensitive (i.e. the "stiff" directions) are relatively few, as the left side of the eigenvalue distribution indicates. Figure G displays the eigenvectors that correspond to the 10 smallest eigenvalues of the covariance matrix, where we can observe the parameters with the greatest contributions to each eigenvector. We can see that the largest components of each eigenvector typically correspond to a few parameters, which exert the greatest control on the quality of the model fit to the data.

Overall, the above analysis confirms that the GATA model demonstrates the common features of large biological models, namely the existence of many parameters that cannot be precisely estimated and display an intricate correlation structure [15]. It also confirms the inadequacy of using point parameter estimates to characterize the behavior of such models and make meaningful predictions for future experiments.

As a final check of model consistency, Table G reports the absolute state values (i.e. not fold changes) for Gat1, Dal80 and Gzf3 mRNA, at the beginning and end of each shift, as predicted from the maximum-

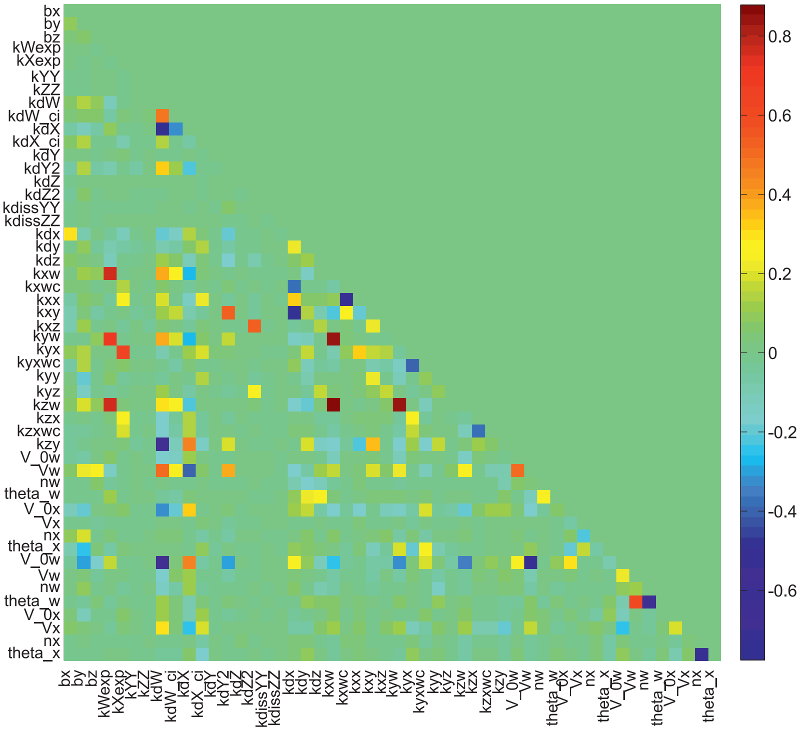

Figure E. Correlation matrix of the posterior parameter sample. As in the previous figure, signal-related parameters are repeated twice. The first repetitions corresponds to Pro→Gln shift, and the second to Gln+Rap.

likelihood parameter set. As we can see, the model reproduces fairly well several qualitative aspects of

Gene mRNA	Gln+Rap, $T = 0$	Gln+Rap, $T = 120$	Pro→Gln, $T = 0$	Pro→Gln, $T = 120$
Gat1	0.1030	0.1455	0.1534	0.0843
Dal80	0.0045	0.0910	0.0795	0.0071
Gzf3	0.0316	0.0245	0.0273	0.0233

Table G. Initial and final values of GATA-factor mRNA abundances

the biological system, despite the fact that it was fitted with fold-change instead of absolute data. More specifically, Dal80 mRNA levels are very low in glutamine, in comparison to the other two transcription factors, which agrees with the fact that Dal80 mRNA is at the limit of detectability in nitrogen-rich conditions, while the Dal80 protein still remains unquantified for the same reason. The situation changes completely in proline, where Dal80 levels exceed Gzf3 levels. The similarity between proline-grown and rapamycin-treated phenotypes is manifested also at the GATA factor mRNA levels, as the third and fourth columns show, while the last column indicates that mRNA levels two hours after the shift to glutamine agree very well with the steady-state levels in glutamine, displayed on the second column.

5.2 Verification of SMC convergence and variability of estimates

In general, verification of SMC convergence is an extremely difficult problem when the correct "answers" (e.g. posterior distribution statistics, marginal likelihood etc.) are not known analytically, and one

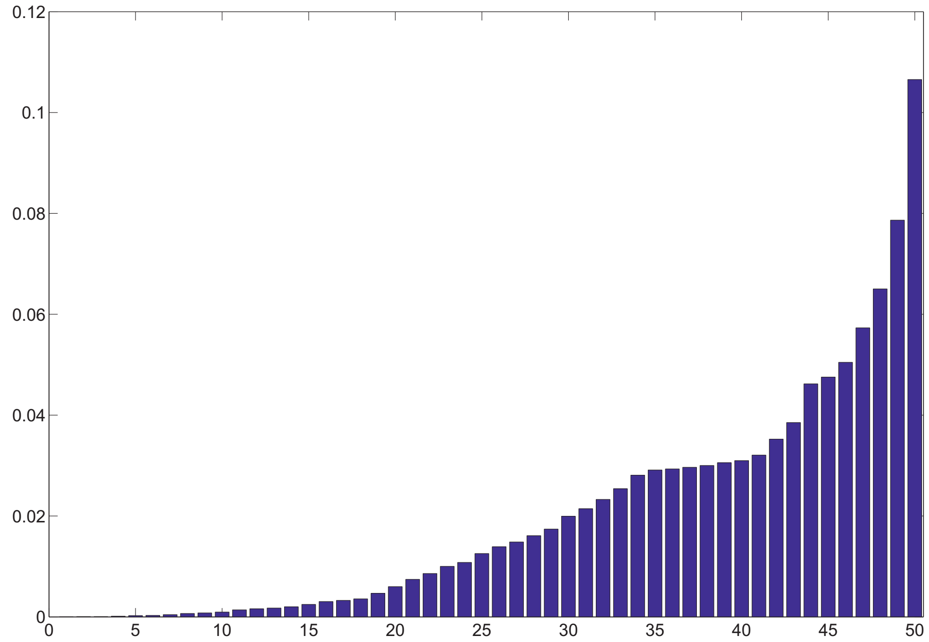

Figure F. Eigenvalues of posterior population covariance matrix Σ, normalized with respect to trace(Σ).

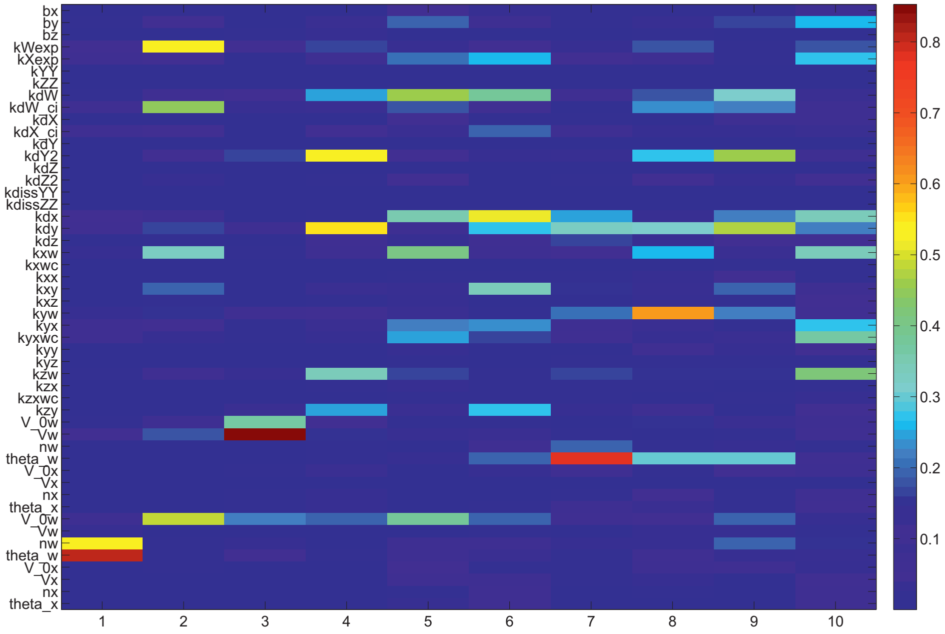

Figure G. Absolute values of eigenvectors corresponding to the 10 smallest eigenvalues of Σ. (following the ordering direction of Figure F)

is only left with heuristics to verify convergence of the algorithm. The safest test, in our opinion, is to run the algorithm many times, starting from different initial samples, and examine the generated

output (posterior samples and evidence estimate). Figs. H, I, J and K show the marginal parameter posteriors obtained from several SMC runs for four GATA TF model structures, while Fig. L contains the corresponding evidence estimates. Finally, Fig. M displays the effective sample size of the particle populations for three different model runs

5.2.1 Sensitivity to the prior

One of the main difficulties with of Bayesian inference, and more specifically model selection, is the sensitivity of the evidence to the choice of priors. To ameliorate this difficulty, we chose the class of log-uniform priors described in Sections 2.6 and 3.3.1, in an effort to sample across a large range of parameter values.

We verified by several runs of the SMC that the evidence of the candidate models is not particularly sensitive to small shifts or scaling of the support intervals of the priors (an example is displayed below). Of course, model ranking may change to a small extent for the closely ranked models of the first model selection round, but this effect is counteracted by our conservative approach at model elimination. Despite these precautions, however, it is still not possible to predict how the evidence of each model would be affected if 20 or 30 parameter priors were varied simultaneously and non-infinitesimally. Unfortunately, to the best of our knowledge, this is an open question.

5.2.2 Random perturbations to the prior:

In this test, we computed the evidence of model $\mathcal{M}_{123}$ (top-ranking in the first model selection round) under small random perturbations of its prior support, by shifting individually the support of every prior in the model by a uniformly distributed random variable in $[-0.2, 0.2]$ (note that the perturbations are not too small, considering that they are done in the log scale). The results are shown on Table H, from which we can conclude that the evidence of $\mathcal{M}_{123}$ is not significantly sensitive to prior perturbations of this magnitude.

Original priors, $\log_{10}(evidence)$:	-24.68, -24.20, -24.06, -24.13, -24.13, -24.01, -23.98
Randomly perturbed priors, $\log_{10}(evidence)$:	-24.37, -23.98, -24.02, -23.94, -23.85

Table H. Individual estimates of model $\mathcal{M}_{123}$ evidence obtained from independent SMC runs under the nominal and randomly perturbed priors, with a different random perturbation applied in each run. The estimate variability in the random perturbation setting does not exceed the variability of the SMC sampler for the nominal case.

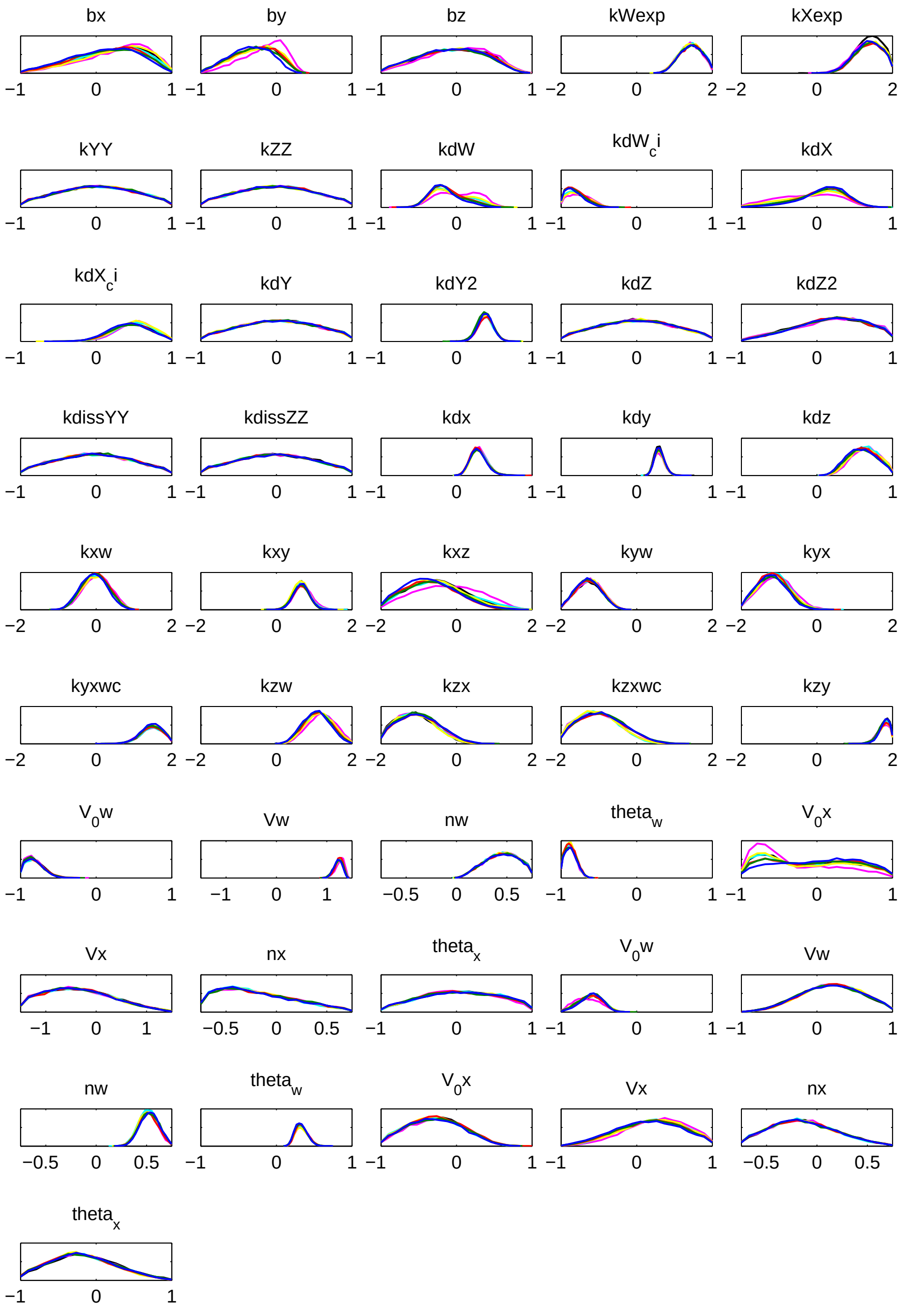

Figure H. Marginal posterior distributions for the parameters of $\mathcal{M}_{123}$ (top-ranking model of the first round), obtained from seven runs of the SMC sampler. Histograms of each run are represented with a different line color. *The x-axis width of each plot denotes the width of the corresponding (log-uniform) parameter prior, to give an idea of the amount of information contained in the data.* Parameters related to translocation signal are repeated twice, since we used data from two shifts. The first set of signal parameters corresponds to Pro→Gln shift, and the second to Gln+Rap. Despite the high dimensionality of the problem, the distributions lie almost on top of each other for almost all parameters, indicating that the converges consistently to the same region of the parameter space.

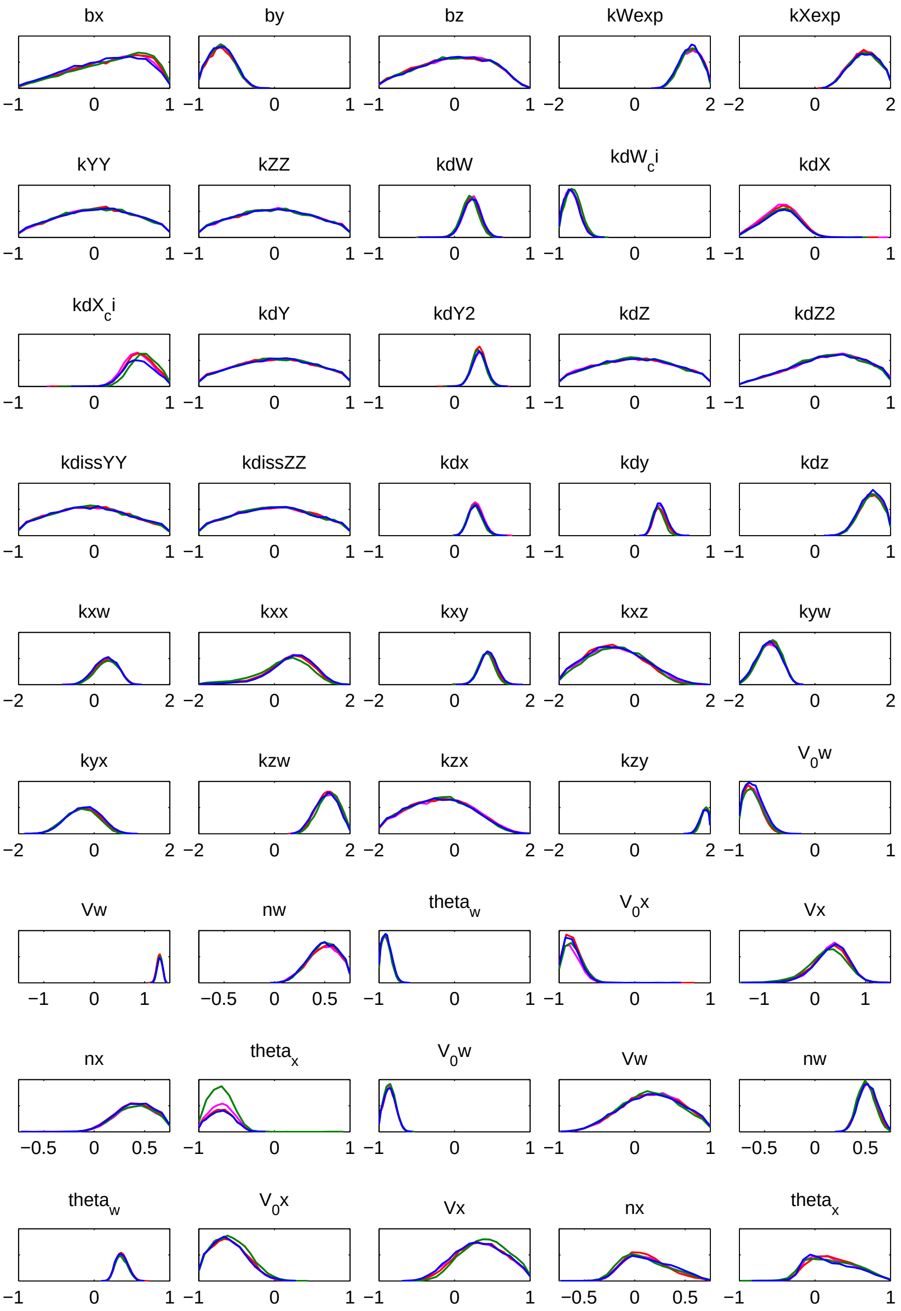

Figure I. Marginal posterior distributions for the parameters of $\mathcal{M}_{135}$ (a high-ranking model of the first round), obtained from four runs of the SMC sampler. Plot details are the same with Fig. H (notice the different number of non-zero parameters contained in the two models). The same consistent behavior of the sampler is observed in this case as well.

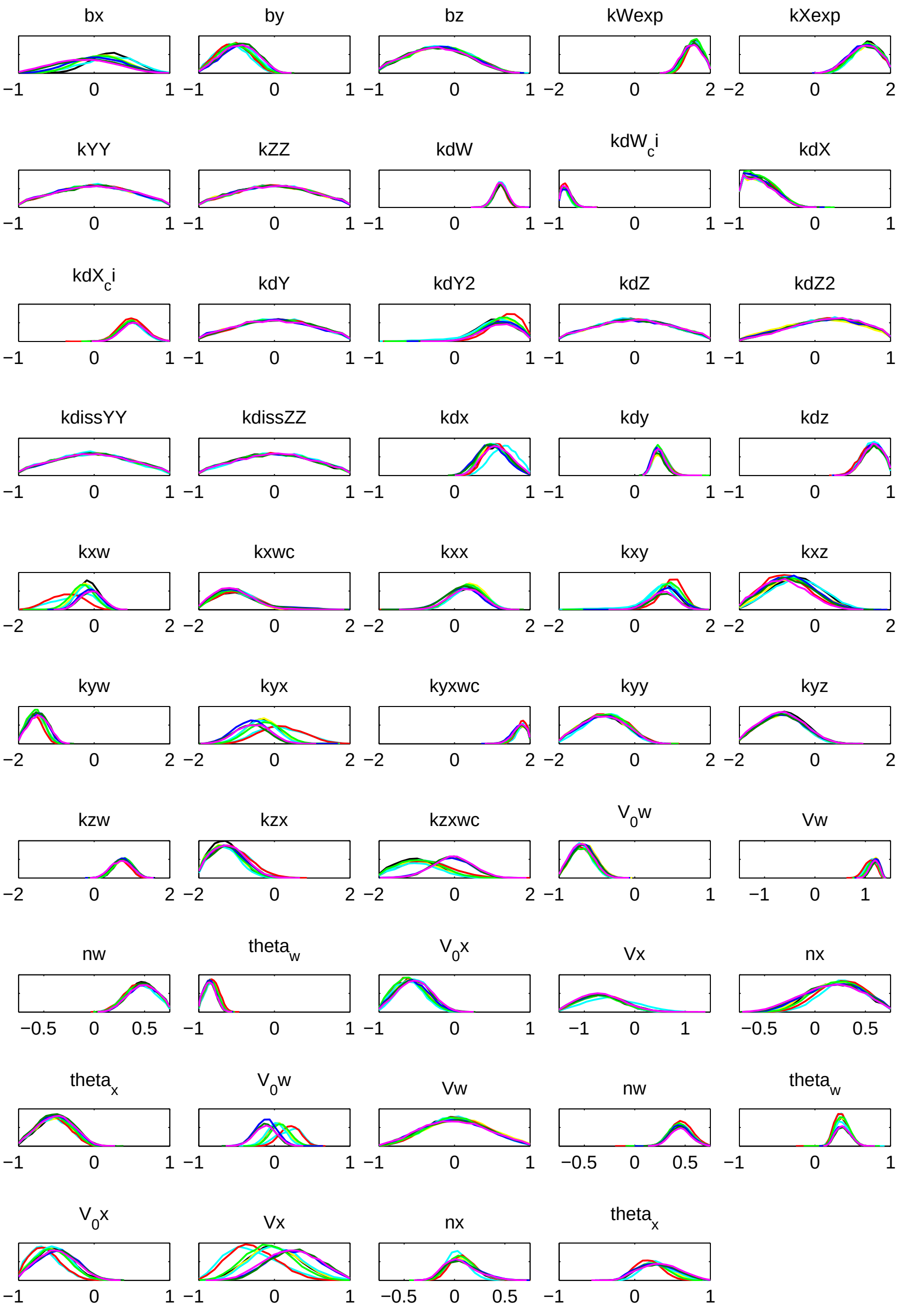

Figure J. Marginal posterior distributions for the parameters of $\mathcal{M}_4$ (a middle-ranking model of the first round), obtained from nine runs of the SMC sampler. Plot details are the same with Fig. H (notice the different number of non-zero parameters contained in the two models). Middle- and low-ranking models present greater difficulties to parameter inference, as they need to be finely tuned to fit the measured data. For this reason, consistency of the SMC sampling deteriorates slightly for a few parameters. This behavior is also mirrored in the evidence estimates shown on Fig. L.

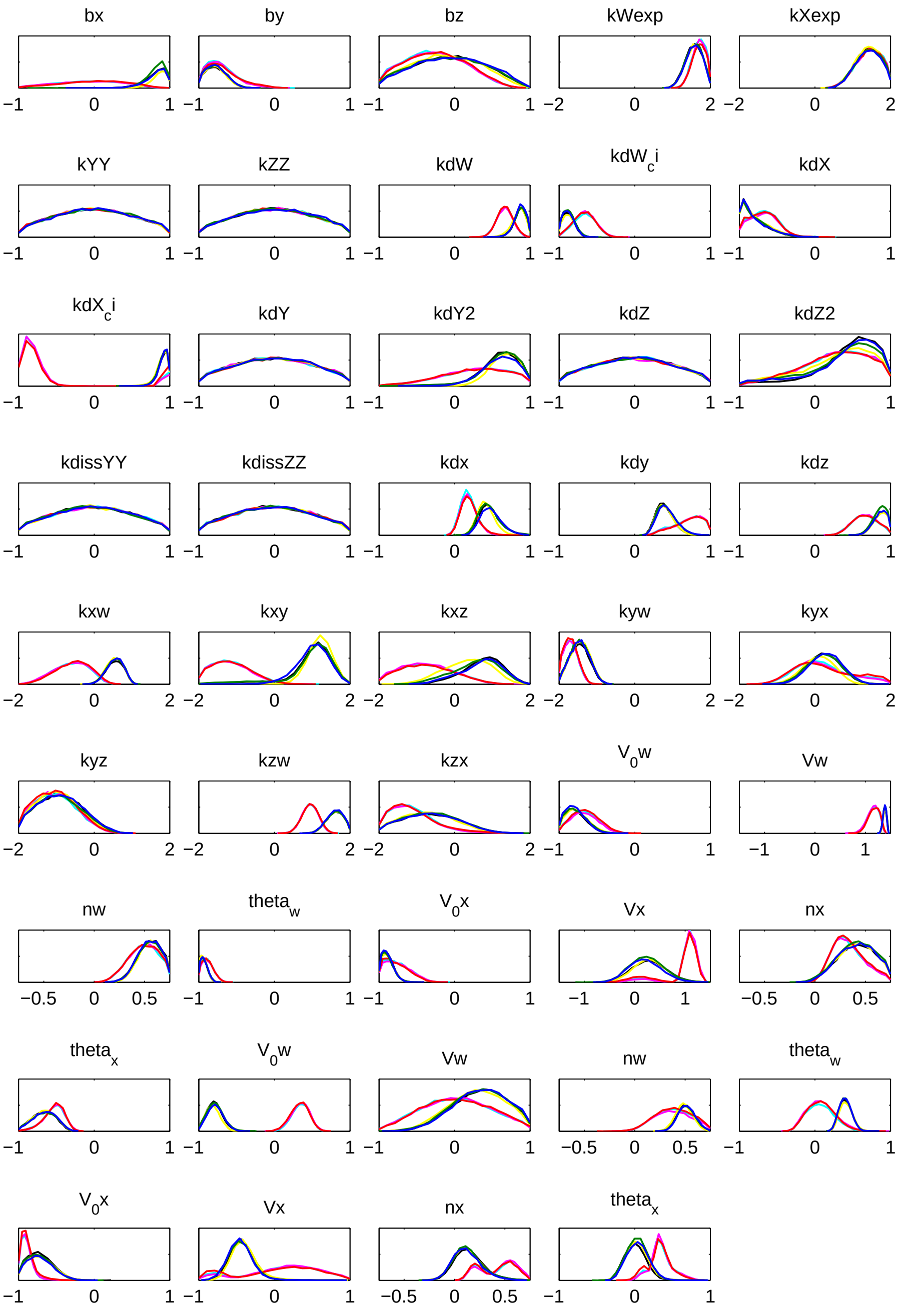

Figure K. Marginal posterior distributions for the parameters of $\mathcal{M}_{1245}$ (the bottom-ranking model of the first round), obtained from seven runs of the SMC sampler. Plot details are the same with Fig. H (notice the different number of non-zero parameters contained in the two models). The evidence of this model is more than 10 orders of magnitude lower than that of $\mathcal{M}_{123}$, indicating that the model structure cannot fit the data properly. It can be seen that the SMC marginal posteriors for several parameters are highly variable between runs, indicating a very rugged likelihood landscape with very low peaks that is not dominated by a clear, high-likelihood peak as in the case of the above models. The same, large variability can be seen in the extremely low evidence estimates on Fig. L.

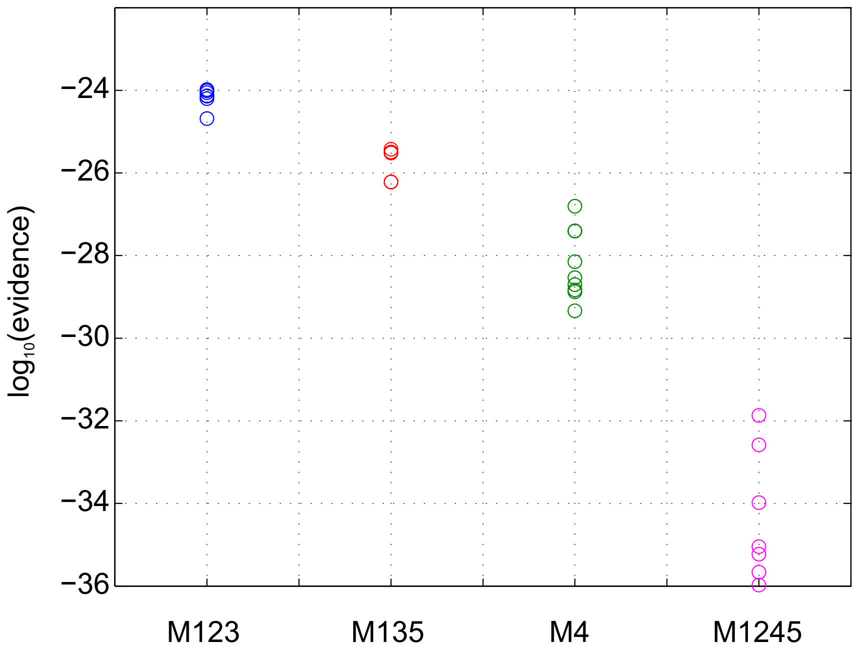

Figure L. Model log-evidence estimates obtained from repeated SMC runs (first model selection round) for the four models considered in the histogram plots above. $\mathcal{M}_{123}$: 7 repetitions. $\mathcal{M}_{135}$: 5 repetitions. $\mathcal{M}_4$: 9 repetitions. $\mathcal{M}_{1245}$: 7 repetitions. It becomes apparent that estimate variability increases as the evidence decreases. As discussed in Figs. J and K, this happens as models become increasingly unable to adequately fit the data and required extremely good fine-tuning. However, this variability does not preclude a clear visual model ranking. Therefore, while individual model ranks in Fig. N may vary by a few positions from run to run, a clear separation between models ranked several positions apart can safely be made.

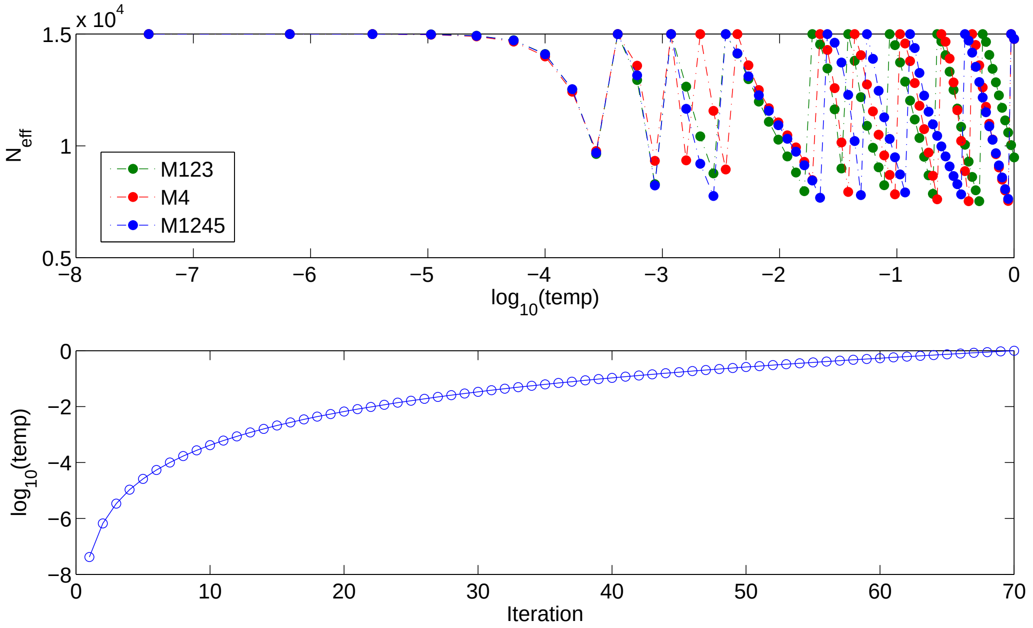

Figure M. Upper panel: effective sample size (N_{eff}) vs. temperature for three different SMC runs, each for a different model: a high-ranking ($\mathcal{M}_{123}$), a middle-ranking ($\mathcal{M}_4$) and low-ranking ($\mathcal{M}_{1245}$). N_{eff} decreases at every temperature step, but is restored by the resampling procedure, which is described in Section 3.4.
Lower panel: logarithm of the temperature schedule used for all models and described in Section 3.4.

5.3 Posterior probability estimates (1st model selection round)

Figure N shows the model posterior probabilities obtained from three individual runs of the SMC algorithm using the GATA TF mRNA data. For each model, the results of each run are denoted with different color and demonstrate the very low variance of the estimates, especially for the top 20 models. Evidence estimates for the low-ranking models are more noisy. This is expected: for such models, only a very small volume of the parameter space can generate predictions that are even roughly compatible with the experimental data. Consequently, locating these regions becomes increasingly hard for the SMC sampler, resulting in higher variance in the evidence estimates. Model ranking is not affected, however, since the low-ranking models are anyway several orders of magnitude less likely than the top-ranking ones.

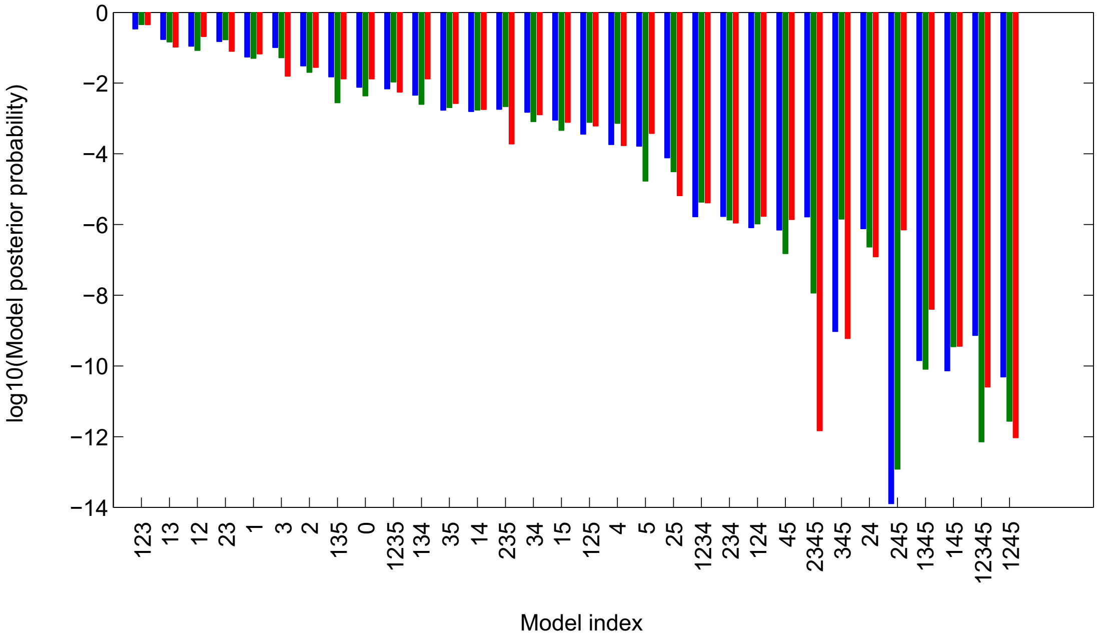

Figure N. Logarithm of posterior model probabilities obtained from the first model selection round: individual runs.

5.4 Multiplicative factor estimates

Table I summarizes the estimates of the factor $F_k(\{D_{target,i}\}_{i=1}^{6})$, for each of the top-ranking models considered in the second model selection round. The estimates from at least two independent SMC runs are shown.

5.5 Broadening selected prior ranges

The histograms of marginal posterior above show that some parameter posteriors end up close to the boundary of the prior distribution. To ensure that this behavior does not affect adversely the results of parameter inference and model selection, we broadened the prior ranges of selected parameters, as shown on Table J below. Figures P, Q and R show a the marginal posterior parameter distributions obtained for a few TF model structures. It can be observed that the posteriors of some parameters shift by a small amount, yet the overall picture remains roughly the same.

Model index	$log_{10}(F_k(\{D_{target,i}\}_{i=1}^{6})$ (2-3 repetitions)
0 (full)	$\{-156.04, -156.11\}$
1	$\{-156.05, -156.86\}$
2	$\{-168.07, -169.06\}$
3	$\{-156.97, -156.98\}$
5	$\{-153.5, -153.6\}$
15	$\{-152.9, -152.8, -153.0\}$
25	$\{-166.0, -164.5\}$
**35**	$\{\mathbf{-148.1}, \mathbf{-147.4}, \mathbf{-148.13}\}$
12	$\{-169.38, -169.95\}$
13	$\{-154.61, -155.60\}$
23	$\{-171.10, -170.39\}$
123	$\{-169.65, -169.67\}$
125	$\{-165.0, -163.4\}$
235	$\{-161.8, -160.4\}$
**135**	$\{\mathbf{-147.66}, \mathbf{-148.05}, \mathbf{-147.61}\}$
1235	$\{-160.59, -160.81\}$

Table I. Monte Carlo estimates of the multiplicative factor F for the augmented GATA models considered in the second model selection round. Despite the very high dimensionality of the parameter space, the estimates turn out remarkably consistent, and allow us to draw strong conclusions about model hypotheses. Models $\mathcal{M}_{135}$ and $\mathcal{M}_{35}$ (rows in bold) rank clearly higher than the third ($\mathcal{M}_{15}$) and all the rest.

Parameter	Shift considered	Old range	New range
k_{exp}^{X}	N/A	$[-2, 2]$	$[-2, \mathbf{2.5}]$
k_{dWi}	N/A	$[-1, 1]$	$[\mathbf{-1.5}, 1]$
k_{zy}	N/A	$[-2, 2]$	$[-2, \mathbf{2.5}]$
k_{dX}	N/A	$[-1, 1]$	$[\mathbf{-1.5}, 1]$
V_{0w}	both	$[-1, 1]$	$[\mathbf{-1.5}, 1]$
θ_w	Pro→Gln	$[-1, 1]$	$[\mathbf{-1.5}, 1]$
V_w	Pro→Gln	$[-1.5, 1.5]$	$[-1.5, \mathbf{2}]$

Table J. Parameters whose prior ranges were increased. The range of each parameter was broadened by 0.5 units in the $\log_{10}$ scale (i.e. increased by about 3.1 times in the linear scale) towards the direction of the boundary that was originally closest to the posterior.

Looking at the evidence estimates under the broadened priors, it appears that the increase of the range results in a moderately (by 1-2 orders of magnitude) increased evidence for all models, but the original ranking is barely affected, as Fig. O shows.

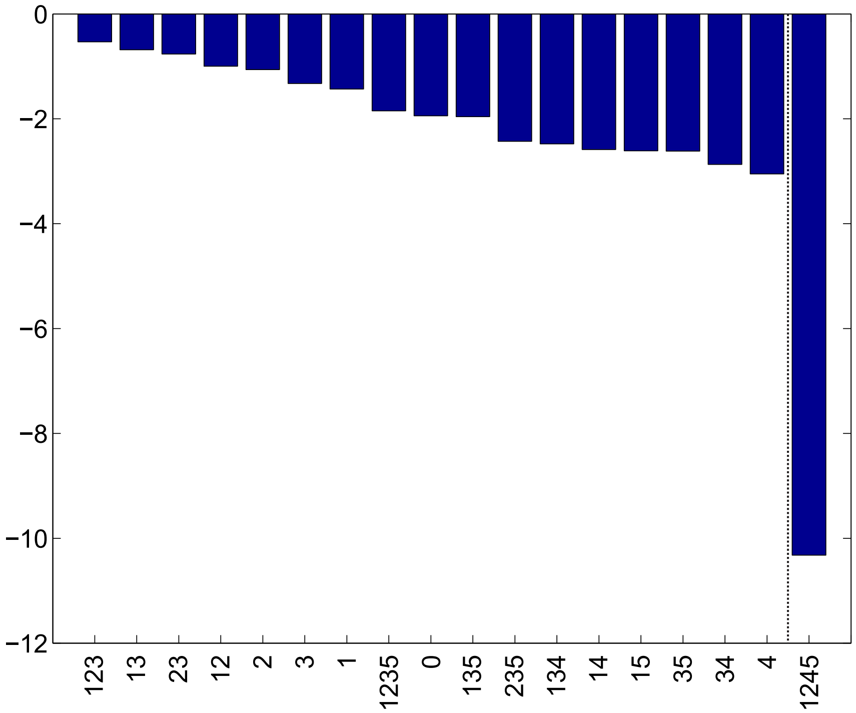

Figure O. Logarithm of posterior model probabilities of the top 17 ranking models of Fig. N (plus the bottom-ranking model) under the broadened priors of Table J. Although individual evidence estimates (not shown) increase by 1-2 orders of magnitude, the posterior model probabilities which depend on the *relative* evidence values (cf. with (5)) remain relatively unaffected. Despite a few exchanges in ranking positions, the overall ranking of Fig. N is preserved, indicating that no model "benefits" excessively by the increased prior ranges.

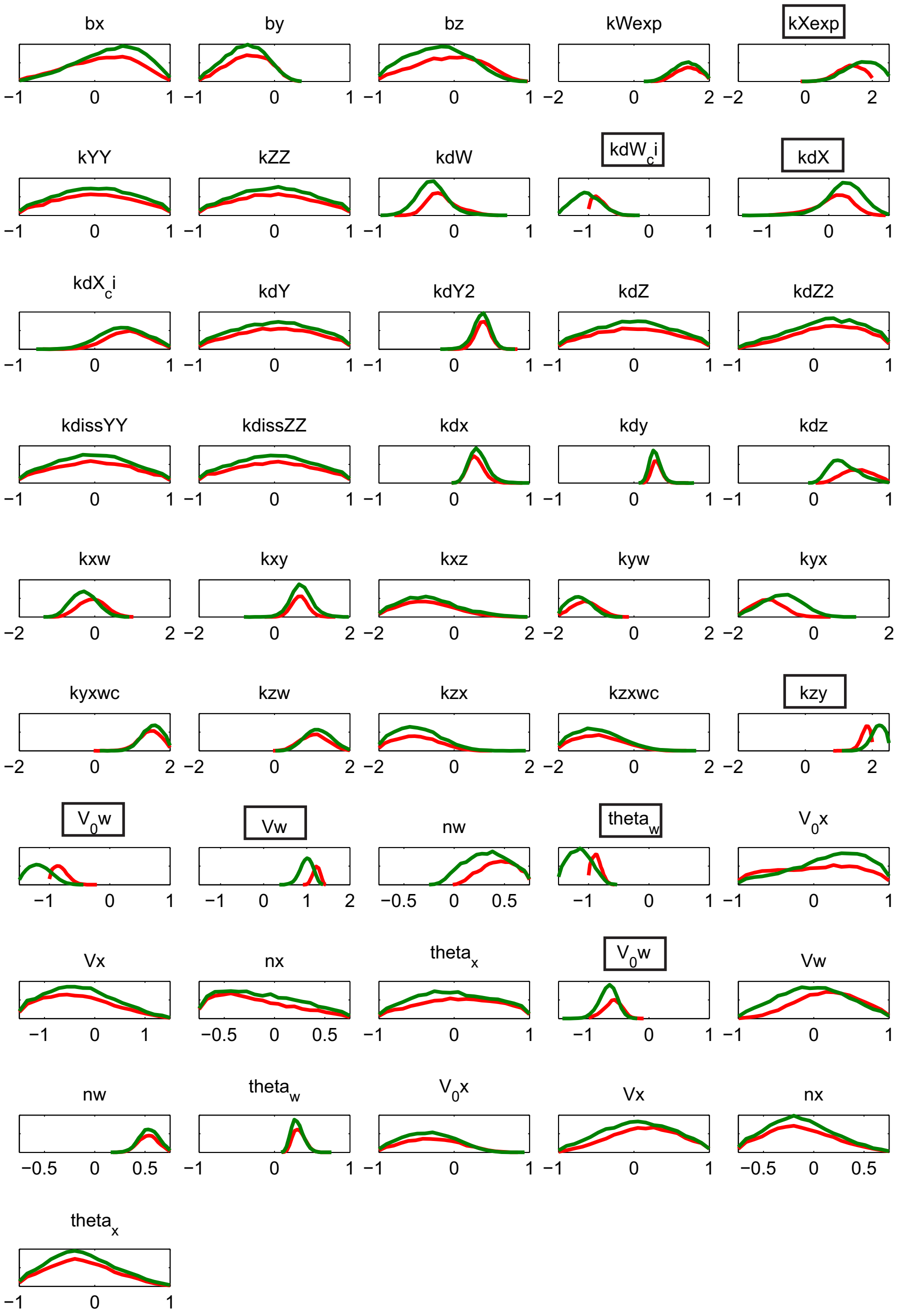

Figure P. Marginal posterior distributions for the parameters of $\mathcal{M}_{123}$, obtained from two different sets of priors. Red line: original priors. Green line: broadened priors reported on Table J. Parameter labels whose prior range was changed are in boxes. The rest of the plot details are the same with Fig. H.

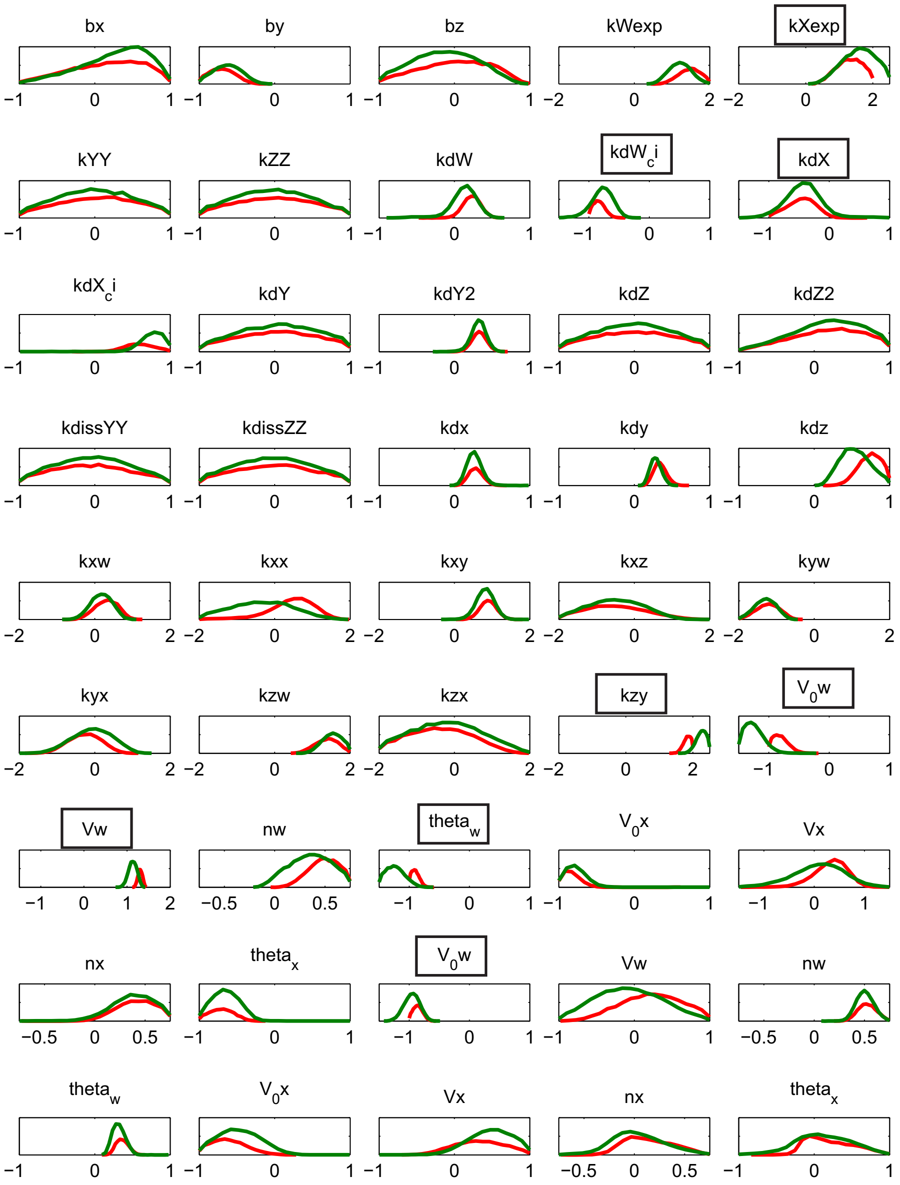

Figure Q. Marginal posterior distributions for the parameters of $\mathcal{M}_{135}$, obtained from two different sets of priors. Red line: original priors. Green line: broadened priors reported on Table J. Parameter labels whose prior range was changed are in boxes. The rest of the plot details are the same with Fig. H.

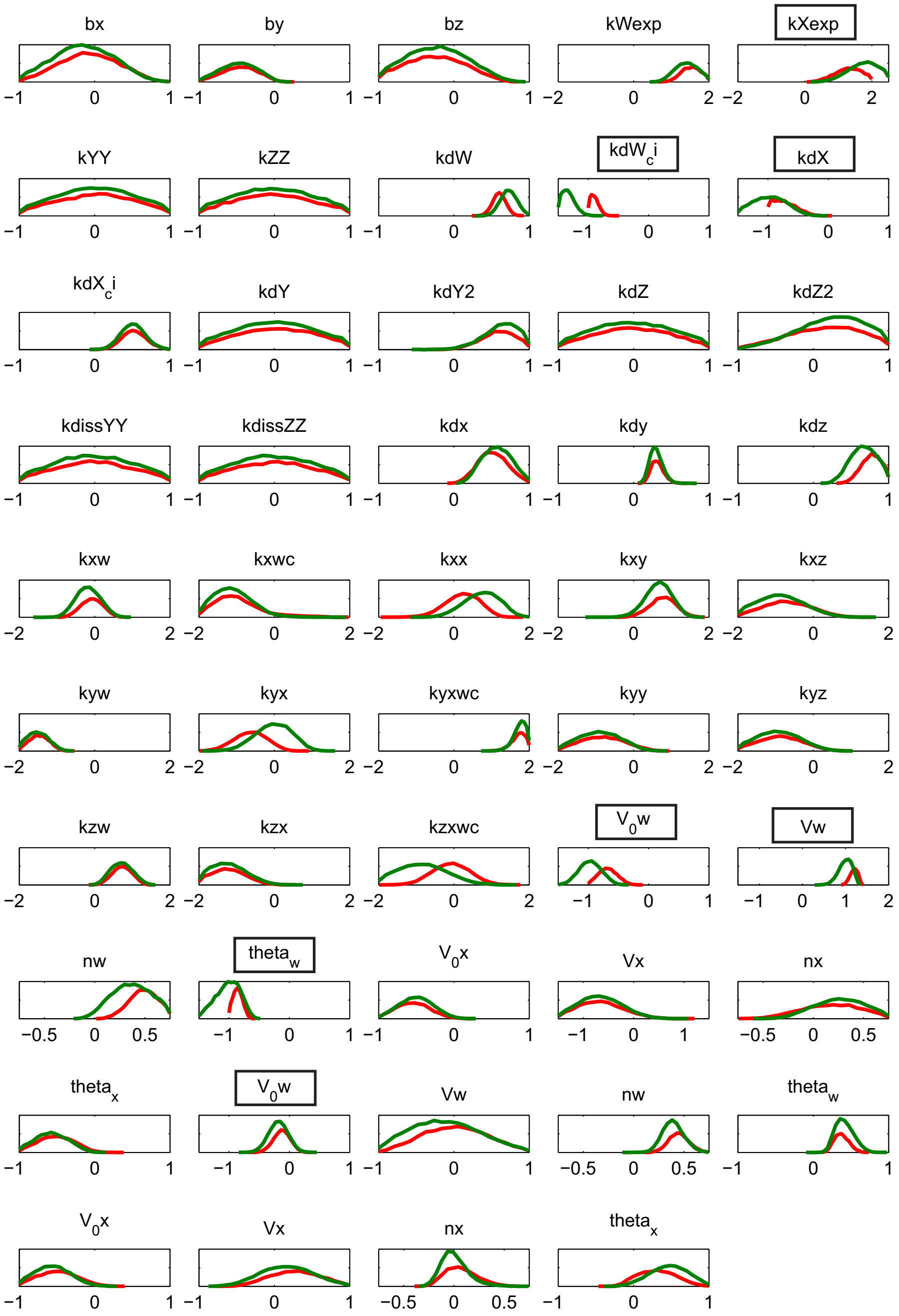

Figure R. Marginal posterior distributions for the parameters of $\mathcal{M}_4$, obtained from two different sets of priors. Red line: original priors. Green line: broadened priors reported on Table J. Parameter labels whose prior range was changed are in boxes. The rest of the plot details are the same with Fig. H.

References for Text S1

1. Andrieu, C., and Thoms, J. A tutorial on adaptive MCMC. *Statistics and Computing 18* (2008), 343–373.

2. Atchadé, Y., and Rosenthal, J. On adaptive Markov chain Monte Carlo algorithms. *Bernoulli 11*, 5 (2005), 815–828.

3. Belle, A., Tanay, A., Bitincka, L., Shamir, R., and OShea, E. K. Quantification of protein half-lives in the budding yeast proteome. *Proceedings of the National Academy of Sciences 103*, 35 (2006), 13004–13009.

4. Beskos, A., Crisan, D., and Jasra, A. On the Stability of Sequential Monte Carlo Methods in High Dimensions. *ArXiv e-prints* (2011).

5. Calderhead, B., and Girolami, M. Estimating Bayes factors via thermodynamic integration and population MCMC. *Computational Statistics & Data Analysis 53*, 12 (2009), 4028 – 4045.

6. Cappé, O., Douc, R., Guillin, A., Marin, J., and Robert, C. Adaptive importance sampling in general mixture classes. *Statistics and Computing 18*, 4 (2008), 447–459.

7. Cappé, O., Guillin, A., Marin, J., and Robert, C. Population monte carlo. *Journal of Computational and Graphical Statistics 13*, 4 (2004), 907–929.

8. Chipman, H., George, E. I., and McCulloch, R. E. The practical implementation of Bayesian model selection. In *Model selection*, vol. 38 of *IMS Lecture Notes Monogr. Ser.* Inst. Math. Statist., 2001, pp. 65–134.

9. Chis, O., Banga, J., and Balsa-Canto, E. Structural identifiability of systems biology models: A critical comparison of methods. *PloS one 6*, 11 (2011), e27755.

10. Coffman, J. A., Rai, R., Cunningham, T., Svetlov, V., and Cooper, T. G. Gat1p, a GATA family protein whose production is sensitive to nitrogen catabolite repression, participates in transcriptional activation of nitrogen-catabolic genes in Saccharomyces cerevisiae. *Molecular and cellular biology 16*, 3 (1996), 847–858.

11. Cornuet, J.-M., Marin, J.-M., Mira, A., and Robert, C. P. Adaptive multiple importance sampling. *Scandinavian Journal of Statistics* (2012).

12. Cox, K. H., Tate, J. J., and Cooper, T. G. Cytoplasmic compartmentation of Gln3 during nitrogen catabolite repression and the mechanism of its nuclear localization during carbon starvation in *Saccharomyces cerevisiae*. *Journal of Biological Chemistry 277*, 40 (2002), 37559–37566.

13. Cunningham, T. S., Andhare, R., and Cooper, T. G. Nitrogen Catabolite Repression of DAL80 expression Depends on the Relative Levels of Gat1p and Ure2p Production in *Saccharomyces cerevisiae*. *Journal of Biological Chemistry 275*, 19 (2000), 14408–14414.

14. Cunningham, T. S., Rai, R., and Cooper, T. G. The Level of DAL80 expression Down-Regulates GATA Factor-Mediated Transcription in *Saccharomyces cerevisiae*. *Journal of bacteriology 182*, 23 (2000), 6584–6591.

15. Erguler, K., and Stumpf, M. Practical limits for reverse engineering of dynamical systems: a statistical analysis of sensitivity and parameter inferability in systems biology models. *Molecular BioSystems 7* (2011), 1593–1602.

16. George, E. I., and McCulloch, R. E. Variable selection via Gibbs sampling. *Journal of the American Statistical Association 88*, 423 (1993), 881–889.

17. Georis, I., Tate, J. J., Cooper, T. G., and Dubois, E. TOR pathway control of the nitrogen-responsive DAL5 gene bifurcates at the level of Gln3 and Gat1 regulation in *Saccharomyces cerevisiae*. *Journal of Biological Chemistry 283*, 14 (2008), 8919–8929.

18. Gordon, A., Colman-Lerner, A., Chin, T. E., Benjamin, K. R., Yu, R. C., and Brent, R. Single-cell quantification of molecules and rates using open-source microscope-based cytometry. *Nature methods 4*, 2 (2007), 175–181.

19. Gutenkunst, R., Waterfall, J., Casey, F., Brown, K., Myers, C., and Sethna, J. Universally sloppy parameter sensitivities in systems biology models. *PLoS Computational Biology 3*, 10 (10 2007), e189.

20. Haario, H., Saksman, E., and Tamminen, J. An adaptive Metropolis algorithm. *Bernoulli 7*, 2 (2001), 223–242.

21. Hansen, A. S., and O'Shea, E. K. Promoter decoding of transcription factor dynamics involves a trade-off between noise and control of gene expression. *Molecular Systems Biology 9*, 1 (2013), 704.

22. Hao, N., Budnik, B. A., Gunawardena, J., and O'Shea, E. K. Tunable signal processing through modular control of transcription factor translocation. *Science 339*, 6118 (2013), 460–464.

23. Iba, Y. Population Monte Carlo algorithms. *Transactions of the Japanese Society for Artificial Intelligence 16* (2001), 279–286.

24. Jasra, A., Stephens, D., and Holmes, C. On population-based simulation for static inference. *Statistics and Computing 17* (2007), 263–279.

25. Kass, R. E., and Raftery, A. E. Bayes factors. *Journal of the American Statistical Association 90*, 430 (1995), 773–795.

26. Liu, J. S., and Chen, R. Sequential Monte Carlo methods for dynamic systems. *Journal of the American Statistical Association 93*, 443 (1998), pp. 1032–1044.

27. McAdams, H. H., and Arkin, A. Stochastic mechanisms in gene expression. *Proceedings of the National Academy of Sciences 94*, 3 (1997), 814–819.

28. McLachlan, G., and Peel, D. *Finite mixture models*, vol. 299. Wiley-Interscience, 2000.

29. Milias-Argeitis, A. *Computational methods for simulation, identification and model selection in systems biology*. PhD thesis, Doctoral dissertation, ETH Zürich, Nr. 21076, 2013, 2013.

30. Miller, C., Schwalb, B., Maier, K., Schulz, D., Dmcke, S., Zacher, B., Mayer, A., Sydow, J., Marcinowski, L., Dlken, L., Martin, D. E., Tresch, A., and Cramer, P. Dynamic transcriptome analysis measures rates of mRNA synthesis and decay in yeast. *Molecular Systems Biology 7*, 1 (2011).

31. Mitchell, T. J., and Beauchamp, J. J. Bayesian variable selection in linear regression. *Journal of the American Statistical Association 83*, 404 (1988), 1023–1032.

32. Moon, T. The expectation-maximization algorithm. *IEEE Signal Processing Magazine 13*, 6 (1996), 47–60.

33. Neal, R. M. Annealed importance sampling. *Statistics and Computing 11* (2001), 125–139.

34. Pelet, S., Dechant, R., Lee, S. S., van Drogen, F., and Peter, M. An integrated image analysis platform to quantify signal transduction in single cells. *Integrative Biology 4*, 10 (2012), 1274–1282.

35. Pelet, S., Rudolf, F., Nadal-Ribelles, M., de Nadal, E., Posas, F., and Peter, M. Transient Activation of the HOG MAPK Pathway Regulates Bimodal Gene Expression. *Science 332*, 6030 (2011), 732–735.

36. Rai, R., Daugherty, J. R., Tate, J. J., Buford, T. D., and Cooper, T. G. Synergistic operation of four cis-acting elements mediate high level DAL5 transcription in *Saccharomyces cerevisiae*. *FEMS Yeast Research 5*, 1 (2004), 29–41.

37. Robert, C. Bayesian computational methods. In *Handbook of Computational Statistics (Volume I) Concepts and Fundamentals*, J. Gentle, W. Hrdle, and Y. Mori, Eds. Springer-Verlag, 2004.

38. Saltelli, A., Ratto, M., Andres, T., Campolongo, F., Cariboni, J., Gatelli, D., Saisana, M., and Tarantola, S. *Global sensitivity analysis: the primer*. Wiley, 2008.

39. Stanbrough, M., Rowen, D. W., and Magasanik, B. Role of the GATA factors Gln3p and Nil1p of Saccharomyces cerevisiae in the expression of nitrogen-regulated genes. *Proceedings of the National Academy of Sciences 92*, 21 (1995), 9450–9454.

40. ter Schure, E. G., van Riel, N. A., and Verrips, C. The role of ammonia metabolism in nitrogen catabolite repression in *Saccharomyces cerevisiae*. *FEMS Microbiology Reviews 24*, 1 (2000), 67–83.

41. Wang, Y., Liu, C. L., Storey, J. D., Tibshirani, R. J., Herschlag, D., and Brown, P. O. Precision and functional specificity in mRNA decay. *Proceedings of the National Academy of Sciences 99*, 9 (2002), 5860–5865.

42. Wraith, D., Kilbinger, M., Benabed, K., Cappé, O., Cardoso, J.-F., Fort, G., Prunet, S., and Robert, C. P. Estimation of cosmological parameters using adaptive importance sampling. *Phys. Rev. D 80* (2009), 023507.

43. Yewdell, J. W., Lacsina, J. R., Rechsteiner, M. C., and Nicchitta, C. V. Out with the old, in with the new? comparing methods for measuring protein degradation. *Cell Biology International 35*, 5 (2011), 457–462.
