# Supplementary material for: Elucidation of Genetic Interactions in the Yeast GATA-Factor Network Using Bayesian Model Selection
Source: PLoS Comput Biol. 2016 Mar 11;12(3):e1004784. doi: 10.1371/journal.pcbi.1004784 (PMC4788432; doi:10.1371/journal.pcbi.1004784)
Supplement: S2 Text — (PDF) [file pcbi.1004784.s002.pdf]

S2 Text. Detailed experimental procedures.

1. Experimental data for model verification

Strains. The prototrophic *Saccharomyces cerevisiae* haploid strain FY4 MATa was used as reference strain. Single gene deletion strains were used from a collection in FY4 isogenic background with the KanMX cassette replacing the Open Reading Frame as in the BY-deletion collection (see Table "Strains employed in the reporter-GFP analysis"). FY4 cells from glycerol stocks were plated in YPD agar plates. Deletion strains from glycerol stocks were plated in YPD agar plates with 300 mg/L of geneticin (G418).

Strains employed in the reporter-GFP analysis

Strain Name	Genotype	Reference
FY4 (reference strain)	FY4 *MATa*	[1]
Δdal80	FY4 *MATa DAL80/YKR034W::kanMX4*	C. Boone*
Δgat1	FY4 *MATa GAT1/YFL021W::kanMX4*	C.Boone*
Δgln3	FY4 *MATa GLN3/YER040W::kanMX4*	C.Boone*
Δgzf3	FY4 *MATa GZF3/YJL110C::kanMX4*	C.Boone*

* Kindly supplied by Prof. Charlie Boone (University of Toronto, Canada)

Gene synthesis and plasmid construction. Nucleotide sequences containing the promoter region of each of the GATA coding genes (-600 to -1 bp uspstream the beginning of the ORF according to the sequence in SGD) immediately upstream of the yeast enhanced GFP gene (yEGFP3 as in [2]; GFP sequence taken from the sequence of plasmid pKT127 [3]), followed by the native terminator of the yeast CDC28 gene (yCDC28t) were designed (see section 2.Custom-designed sequences below) and synthesized by GeneArt AG (Regensburg, Germany). The custom-designed sequences were cloned into the vector pRS41H [4] using the restriction enzymes NotI and SalI, thereafter generating the plasmids pRS41H_pDAL80-GFP, pRS41H_pGAT1-GFP, pRS41H_pGLN3-GFP and pRS41H_pGZF3-GFP (see Table "Vector and plasmids employed in the reporter-GFP analysis"). The inserted sequences were verified by sequencing. The expected plasmid size was verified by digestion with NdeI, which generated the two expected fragments including one cut inside the inserted sequence.

Vector and plasmids employed in the reporter-GFP analysis

Plasmid	Vector; Insert	Insert description	Reference
pRS41H	pRS41H (features: *CEN*, *HygR*, *AmpR*)	empty vector; for background correction	[4], *
pRS41H_pDAL80-GFP	pRS41H; pDAL80(-600,-1) + yEGFP3 + yCDC28t	*DAL80* native promote, upstream of GFP	This study
pRS41H_pGAT1-GFP	pRS41H; pGAT1(-600,-1) + yEGFP3 + yCDC28t	*GAT1* native promoter, upstream of GFP	This study
pRS41H_pGLN3-GFP	pRS41H; pGLN3(-600,-1) + yEGFP3 + yCDC28t	*GLN3* native promoter, upstream of GFP	This study
pRS41H_pGZF3-GFP	pRS41H; pGZF3(-600,-1) + yEGFP3 + yCDC28t	*GZF3* native promoter, upstream of GFP	This study

* Kindly supplied by Prof. Michael Knop (Heidelberg University, Germany)

Plasmid propagation and yeast transformations. The constructed plasmids were transformed by electroporation into *Escherichia coli* DH5α, transformed cells were selected in LB plates with ampicillin, and plasmids were recovered and purified by standard Mini-Prep kits. After verification by sequencing and digestion, each plasmid was transformed into *S. cerevisiae* using a lithium-acetate transformation protocol, including a 4-6 hour growth in YPD for expression of the Hygromycin resistance gene as described in [4]. Transformed yeast cells were plated into selective YPD agar plates with 300 mg/L hygromycin B.

Media composition. The Yeast Minimal Medium (YMM) used in precultures and main cultures was based on [5], with a few modifications. The basis of the minimal mineral medium, excluding a nitrogen- and a carbon-source, contained the following components per Liter: 5g K_2SO_4, 3g KH_2PO_4, 0.5g $MgSO_4 \cdot 7H_2O$, 15mg EDTA, 4.5mg $ZnSO_4 \cdot 7H_2O$, 0.3mg $CoCl_2 \cdot 6H_2O$, 1.0mg $MnCl_2 \cdot 4H_2O$, 0.3mg $CuSO_4 \cdot 5H_2O$, 4.5mg $CaCl_2 \cdot 2H_2O$, 3.0mg $FeSO_4 \cdot$7H2O, 0.4mg $NaMoO_4 \cdot 2H_2O$, 1.0mg H_3BO_3, 0.1mg KI, , 0.05mg biotin, 1.0mg calcium pantothenate, 1.0mg nicotinic acid, 25mg inositol, 1.0mg pyridoxine, 0.2mg p-aminobenzoic acid, 1.0mg thiamin, and 10 mM potassium hydrogen phthalate buffer (pH 5). All culture media were supplemented with D-glucose to a final concentration of 20 g/L and hygromycin B to a final concentration of 300 mg/L. Nitrogen source supplementation was as followed at the beginning of the cultures: “pre-culture-YMM” was supplemented with 1 g/L of ammonium sulfate; “glutamine-YMM” was supplemented with 0.5 g/L of L-glutamine; and “proline-YMM” was supplemented with 0.78 g/L of L-proline.

Cultivation conditions and induction of dynamic shifts. One single colony from freshly transformed yeast strains was pre-inoculated into 3-mL of “pre-culture-YMM” and grown for 20-24 hours at 30 °C until saturation. Afterwards, 10-20 µL of pre-inoculum was inoculated into 1.5 mL of the appropriate media within each well of a 48-well microtiter Flower plate (m2p labs, Aachen, Germany), thereafter sealed with Gas Permeable Adhesive seals (Thermo Scientific, Surrey, UK). The microtiter plate was incubated into a BioLector device (m2p-labs, Aachen, Germany) [6], set for 30°C temperature, 1000 rpm shaking frequency, 95% humidity (controlled), and reading scattered light (620 nm, Gain: 25) and GFP (excitation: 488 nm/ emission: 520 nm, gain: 100) signals every 5-7 minutes. Biomass and GFP evolution was monitored online via the Biolection software.

For the rapamycin pulse, yeast strains were initially inoculated into “glutamine-YMM”. When cells achieved a mid-exponential growth as estimated from the online monitoring readings, Biolector was paused, the plate removed and a 30x concentrated stock of rapamycin was added to each well to a final concentration of 400 µg/L. Biolector monitoring was resumed within 3 minutes after pausing. For the proline->glutamine upshift, yeast strains were initially inoculated into “proline-YMM”. As before, when cells achieved a mid-exponential growth, a 30x concentrated stock of L-glutamine was added to each well to a final concentration of 0.5 g/L.

2. Custom-designed DNA fragments

Custom-designed DNA fragments containing each of the GATA promoters upstream of a yeast enhanced GFP (yEGFP), followed by the terminator of *CDC28* yeast gene were designed as follows.

Restriction site's enzymes: NotI XmaI BamHI HindIII SalI

> pDAL80(-600,-1) + yEGFP3 + yCDC28t

GTTGTTGCGGCCGCTCCCGGGTGGATCCTCTGGTCTCGAATTTACTCTATCTACTTCTTCAGTCACCGATCTATCTGC
CTGAGGTGGAGCGAAGTGAGTTCCAGACGCTGTTGACACGTTAGACATCACCCTTGTTTATCTATCCTACCTTTTCT
TCTTGCGTACGTGCCTCTCAATGCGTCGTGTGAATTATCAGTGACCGGTCGTGCCTATAATGTCCTGCTAATTTCCC
ACTAAATCTTTCCCCATGGCGTATTCATCGTTATGTTTGTGTCTTTTGTTCAACCCAAAGGGCTGTAGCAATCTTCAC
CCGTTTGTCGTTGATAACGAGTTTCCACCTTATCACTTATCACTAGTGCTAATCAAACAGCAAAGAATGCTTGATAG
AAACCGATCCTGGGCTTATCTCGCTGCATTGTGGCGGCATCCCTGGACTGTAATCAGCAAGTGTTGCTTAGTATAT
ATATACATCCAGCGTCAGCTTGAATTTGGATACAGTTACTGTTTTTTCGATTTTCTCTTGGTTATTCTTTCTGAGACA
GTAGTAATTTTGTATTACTGAGCGGGATATTGTTTATCTGCCGTCATACTATATTACATTATATTATATCATATTATA
TATAAGAGAATGTCTAAAGGTGAAGAATTATTCACTGGTGTTGTCCCAATTTTGGTTGAATTAGATGGTGATGTTA
ATGGTCACAAATTTTCTGTCTCCGGTGAAGGTGAAGGTGATGCTACTTACGGTAAATTGACCTTAAAATTTATTTGT
ACTACTGGTAAATTGCCAGTTCCATGGCCAACCTTAGTCACTACTTTAACTTATGGTGTTCAATGTTTTTCTAGATAC
CCAGATCATATGAAACAACATGACTTTTTCAAGTCTGCCATGCCAGAAGGTTATGTTCAAGAAAGAACTATTTTTTT
CAAAGATGACGGTAACTACAAGACCAGAGCTGAAGTCAAGTTTGAAGGTGATACCTTAGTTAATAGAATCGAATT
AAAAGGTATTGATTTTAAAGAAGATGGTAACATTTTAGGTCACAAATTGGAATACAACTATAACTCTCACAATGTTT
ACATCATGGCTGACAAACAAAAGAATGGTATCAAAGTTAACTTCAAAATTAGACACAACATTGAAGATGGTTCTGT
TCAATTAGCTGACCATTATCAACAAAATACTCCAATTGGTGATGGTCCAGTCTTGTTACCAGACAACCATTACTTAT
CCACTCAATCTGCCTTATCCAAAGATCCAAACGAAAAGAGAGACCACATGGTCTTGTTAGAATTTGTTACTGCTGCT
GGTATTACCCATGGTATGGATGAATTGTACAAATAAAAGCTTGCATTATAATCTATTTCGCTATTATATTACAAATG
CTACTGCACTGTCATTATAGCCTAGTAAAGTATATAGTGAATACAATATACTCAGTTTCAACATTATGATGGGTAAC
TCCATCAGAAAATATATTCATCGTCATATACGGAACATTCAGTTATACGCGAAAGTAAAAGTGAGAGCTTTTCAGG
GGTTAAAAGCTGGGCGTGTTCCATGACGTATTTACCGAGGTCGTATTATCAAAGAAAATGAAAAAAAAAAAAAAA
AAAAAAAAAGCGAAGAGGAAAAAGACAAACGAAAAACAAAACGAAATAACGCATTGTATGCTGGGGCCTCAATT
GAACTTCTTTGACGTCGACAACAAC

> pGAT1(-600,-1) + yEGFP3 + yCDC28t

GTTGTTGCGGCCGCTCCCGGGTGGATCCCCCGCTTTCCGATTTTAGCCGGCGAAGACGTACTTGGCGCCATAATCA
AAACCTAGCTTGCCCAATACTTCTGAGTTCTACGTGGTGCAAAAATATTTTTTTTTTTTGAAAAACCTACCCTATTT
CATTATAGATGCATCCATCAGTATTACGGTGTCCTCACACAACCCTGTCTCTGCACAACGTAATACCTCCTTTTCCCG
TCTGCTAGCTCTCATTTCGCGGTAATCCAACTTCAACCAGCAACCCGGATCTTCTATACGCAGTCCGGTGTGTGGGT
GCATGACTGATTGGTCCGGCCGATAACAGGTGTGCTTGCACCCAGTGCCCAACGTCAACAAAGCAGGAACAACGG
GCTGATAAGGGAGAAGATAAGATAAGATAAGATAACAAATCATTGCGTCCGACCACAGGCCGACACATAGCAGA
ACGATGTGAAGCAGCGCAGCATAGTGTTAGTGCCGGTGCAGCTACCGCTGGTATTAACAGCCACCACAATACAGA
GCAACAATAATAACAGCACTATGAGTCGCACACTTGCGGTGCCCGGCCCAGCCACATATATATAGGTGTGTGCCAC
TCCCGGCCCCGGTATTAGCATGTCTAAAGGTGAAGAATTATTCACTGGTGTTGTCCCAATTTTGGTTGAATTAGATG
GTGATGTTAATGGTCACAAATTTTCTGTCTCCGGTGAAGGTGAAGGTGATGCTACTTACGGTAAATTGACCTTAAA
ATTTATTTGTACTACTGGTAAATTGCCAGTTCCATGGCCAACCTTAGTCACTACTTTAACTTATGGTGTTCAATGTTT
TTCTAGATACCCAGATCATATGAAACAACATGACTTTTTCAAGTCTGCCATGCCAGAAGGTTATGTTCAAGAAAGA
ACTATTTTTTTCAAAGATGACGGTAACTACAAGACCAGAGCTGAAGTCAAGTTTGAAGGTGATACCTTAGTTAATA
GAATCGAATTAAAAGGTATTGATTTTAAAGAAGATGGTAACATTTTAGGTCACAAATTGGAATACAACTATAACTC
TCACAATGTTTACATCATGGCTGACAAACAAAAGAATGGTATCAAAGTTAACTTCAAAATTAGACACAACATTGAA
GATGGTTCTGTTCAATTAGCTGACCATTATCAACAAAATACTCCAATTGGTGATGGTCCAGTCTTGTTACCAGACAA
CCATTACTTATCCACTCAATCTGCCTTATCCAAAGATCCAAACGAAAAGAGAGACCACATGGTCTTGTTAGAATTTG
TTACTGCTGCTGGTATTACCCATGGTATGGATGAATTGTACAAATAAAAGCTTGCATTATAATCTATTTCGCTATTAT
ATTACAAATGCTACTGCACTGTCATTATAGCCTAGTAAAGTATATAGTGAATACAATATACTCAGTTTCAACATTAT
GATGGGTAACTCCATCAGAAAATATATTCATCGTCATATACGGAACATTCAGTTATACGCGAAAGTAAAAGTGAGA
GCTTTTCAGGGGTTAAAAGCTGGGCGTGTTCCATGACGTATTTACCGAGGTCGTATTATCAAAGAAAATGAAAAA
AAAAAAAAAAAAAAAAAAAGCGAAGAGGAAAAAGACAAACGAAAAACAAAACGAAATAACGCATTGTATGCTG
GGGCCTCAATTGAACTTCTTTGACGTCGACAACAAC

> GLN3(-600,-1) + yEGFP3 + yCDC28t

GTTGTTGCGGCCGCTCCCGGGTGGATCCGTATAAATCATCAATACGAGCAGCAAAGAAATTGGAAACCAGTTTTTT
ACATCTGTCCTGTTCAAAGATCAAAAATTAGCAACGCCTACAATTCGTAGGATACATAGCGTCACAGTGCACACCA
GTGATTGTACAAACAACATCACAAAGTTCATGTTAAAGTTGTCCAGGTTAACCACGAATTTGTTCGTTACTGTCATC
AAAATCGAGGACGCGCAGTAAGATAAGATTGAAGCCGGCCCAGAGTTGGCCACTGATTCCGTCCATTCATGCTTA
TGCTTGCTCATAATTACCACACCTTCTTGATCTCTTTACAGCTTTTCAACCTTCCATTCTTGTACTCTATCTCTACCTGG
CCCTTTAAACATTCTTAATATGATATATTCACATTTTTTGCTCTATTACCCGGCGGACAGGTTCCCGAAAGAAAGTG
ACATGGCAATGCTGAGAGAGTGGAAAGAGTCATCTTGCAAGACAGAGAAAGATGTTCAAGAGTGGTAAGCTAAT
GTCAGCGCAGTAGCCCATCCCACAATAACAGAGTGTGTAAGAAAGAGAGACGAGAGAGAGCACAGGGCCCCCTT
TTCCCCCACCAACAAACAAATGTCTAAAGGTGAAGAATTATTCACTGGTGTTGTCCCAATTTTGGTTGAATTAGATG
GTGATGTTAATGGTCACAAATTTTCTGTCTCCGGTGAAGGTGAAGGTGATGCTACTTACGGTAAATTGACCTTAAA
ATTTATTTGTACTACTGGTAAATTGCCAGTTCCATGGCCAACCTTAGTCACTACTTTAACTTATGGTGTTCAATGTTT

TTCTAGATACCCAGATCATATGAAACAACATGACTTTTTCAAGTCTGCCATGCCAGAAGGTTATGTTCAAGAAAGA
ACTATTTTTTTCAAAGATGACGGTAACTACAAGACCAGAGCTGAAGTCAAGTTTGAAGGTGATACCTTAGTTAATA
GAATCGAATTAAAAGGTATTGATTTTAAAGAAGATGGTAACATTTTAGGTCACAAATTGGAATACAACTATAACTC
TCACAATGTTTACATCATGGCTGACAAACAAAAGAATGGTATCAAAGTTAACTTCAAAATTAGACACAACATTGAA
GATGGTTCTGTTCAATTAGCTGACCATTATCAACAAAATACTCCAATTGGTGATGGTCCAGTCTTGTTACCAGACAA
CCATTACTTATCCACTCAATCTGCCTTATCCAAAGATCCAAACGAAAAGAGAGACCACATGGTCTTGTTAGAATTTG
TTACTGCTGCTGGTATTACCCATGGTATGGATGAATTGTACAAATAAAAGCTTGCATTATAATCTATTTCGCTATTAT
ATTACAAATGCTACTGCACTGTCATTATAGCCTAGTAAAGTATATAGTGAATACAATATACTCAGTTTCAACATTAT
GATGGGTAACTCCATCAGAAAATATATTCATCGTCATATACGGAACATTCAGTTATACGCGAAAGTAAAAGTGAGA
GCTTTTCAGGGGTTAAAAGCTGGGCGTGTTCCATGACGTATTTACCGAGGTCGTATTATCAAAGAAAATGAAAAA
AAAAAAAAAAAAAAAAAAAAGCGAAGAGGAAAAAGACAAACGAAAAACAAAACGAAATAACGCATTGTATGCTG
GGGCCTCAATTGAACTTCTTTGACGTCGACAACAAC

> pGZF3(-600,-1) + yEGFP3 + yCDC28t

GTTGTTGCGGCCGCTCCCGGGTGGATCCGAAGTTGATATACTCAAAGATAGGTGAAAGTTGGTTAGTTCTATTGCC
ACAGCTCGTTCCCGTCATCGCTGAGTTATTGGAAGATGATGATGAAGAGATCGAACGTGAAGTCAGGACCGGTTT
GGTCAAGGTTGTTGAAAACGTTTTAGGGGAACCTTTTGATAGGTATTTAGATTAGAAAAAAAAGGTGAAGTATTA
TGTAAATTTTTGTAAAGTAAAACACTATGCTGTTGAACGAAATCTTTCATTGAAAATATTGTTATTCATTCGTGATA
GCTGCCCCTTTCTGAGTTTGAACTTAATATTTCAATTACGCTACTTCAAGTTTCAATGAGATATTATTCTGTCATCTTT
CTCGTCGTTCCTAGTGATTAACGTTACTAAAATTACTGATCCTAAATAGCGGGCGAACAGAGTGAAAATTTTCTTAT
CTTCGCTTATCTGCGCTTATCAATCCTAATCAGTGAAAAATAAGATATAGGCTTGATAATAAGGTAGTTTGAAAGA
GAACATATTGCAAGCGGTTGAAGCTATAATACTAGATATACGAATATCATTTCGGGTATTTGTACTGTGCTCTACAA
TTCTACTGGTAATATTATGTCTAAAGGTGAAGAATTATTCACTGGTGTTGTCCCAATTTTGGTTGAATTAGATGGTG
ATGTTAATGGTCACAAATTTTCTGTCTCCGGTGAAGGTGAAGGTGATGCTACTTACGGTAAATTGACCTTAAAATTT
ATTTGTACTACTGGTAAATTGCCAGTTCCATGGCCAACCTTAGTCACTACTTTAACTTATGGTGTTCAATGTTTTTCT
AGATACCCAGATCATATGAAACAACATGACTTTTTCAAGTCTGCCATGCCAGAAGGTTATGTTCAAGAAAGAACTA
TTTTTTTCAAAGATGACGGTAACTACAAGACCAGAGCTGAAGTCAAGTTTGAAGGTGATACCTTAGTTAATAGAAT
CGAATTAAAAGGTATTGATTTTAAAGAAGATGGTAACATTTTAGGTCACAAATTGGAATACAACTATAACTCTCAC
AATGTTTACATCATGGCTGACAAACAAAAGAATGGTATCAAAGTTAACTTCAAAATTAGACACAACATTGAAGATG
GTTCTGTTCAATTAGCTGACCATTATCAACAAAATACTCCAATTGGTGATGGTCCAGTCTTGTTACCAGACAACCAT
TACTTATCCACTCAATCTGCCTTATCCAAAGATCCAAACGAAAAGAGAGACCACATGGTCTTGTTAGAATTTGTTAC
TGCTGCTGGTATTACCCATGGTATGGATGAATTGTACAAATAAAAGCTTGCATTATAATCTATTTCGCTATTATATT
ACAAATGCTACTGCACTGTCATTATAGCCTAGTAAAGTATATAGTGAATACAATATACTCAGTTTCAACATTATGAT
GGGTAACTCCATCAGAAAATATATTCATCGTCATATACGGAACATTCAGTTATACGCGAAAGTAAAAGTGAGAGCT
TTTCAGGGGTTAAAAGCTGGGCGTGTTCCATGACGTATTTACCGAGGTCGTATTATCAAAGAAAATGAAAAAAAA
AAAAAAAAAAAAAAAAAGCGAAGAGGAAAAAGACAAACGAAAAACAAAACGAAATAACGCATTGTATGCTGGGG
CCTCAATTGAACTTCTTTGACGTCGACAACAAC

References for Text S2

1. Winston F, Dollard C, Ricupero-Hovasse SL (1995) Construction of a set of convenient Saccharomyces cerevisiae strains that are isogenic to S288C. Yeast 11: 53-55.
2. Cormack BP, Bertram G, Egerton M, Gow NA, Falkow S, et al. (1997) Yeast-enhanced green fluorescent protein (yEGFP): a reporter of gene expression in Candida albicans. Microbiology 143 (Pt 2): 303-311.
3. Sheff MA, Thorn KS (2004) Optimized cassettes for fluorescent protein tagging in Saccharomyces cerevisiae. Yeast 21: 661-670.
4. Taxis C, Knop M (2006) System of centromeric, episomal, and integrative vectors based on drug resistance markers for Saccharomyces cerevisiae. Biotechniques 40: 73-78.
5. Blank LM, Sauer U (2004) TCA cycle activity in Saccharomyces cerevisiae is a function of the environmentally determined specific growth and glucose uptake rates. Microbiology 150: 1085-1093.
6. Samorski M, Muller-Newen G, Buchs J (2005) Quasi-continuous combined scattered light and fluorescence measurements: a novel measurement technique for shaken microtiter plates. Biotechnol Bioeng 92: 61-68.
