# Supplementary material for: Elucidation of Genetic Interactions in the Yeast GATA-Factor Network Using Bayesian Model Selection
Source: PLoS Comput Biol. 2016 Mar 11;12(3):e1004784. doi: 10.1371/journal.pcbi.1004784 (PMC4788432; doi:10.1371/journal.pcbi.1004784)
Supplement: S1 File — (ZIP) [file pcbi.1004784.s004.zip › SBML_model.zip!/readme.docx]

**Running the SBML model**

To simulate the SBML version of our model, the user will first have to import the SBML file into simulation software that allows the definition of time-varying reaction rates. Then, the activation rates of Gln3 and Gat1 (parameters $k_{1w}$ and $k_{1x}$) will need to be assigned the functional form given in the Methods section of the main text, depending on the shift that is simulated. Moreover, the Gln3 data (given below) will need to be interpolated over time, to be used on the right-hand side of the GLN3 protein production ODE.

**Gln3 data, Pro 🡪 Gln shift**:

Time (min.): [0 3 7 10 14 24 56 80]

mRNA (fold change): [1 0.8397 1.0933 1.2902 1.422 2.1757 2.2612 1.1216]

**Gln3 data, Gln+Rap shift**:

Time (min.): [0 3 7 10 14 24 56 80]

mRNA (fold change): [1 0.9411 0.9462 0.7593 0.7259 0.9239 1.0359 0.8232]
